# Chlorination of Amines by a Vanadium-Dependent Chloroperoxidase

**DOI:** 10.1021/acscatal.6c00816

**Published:** 2026-03-23

**Authors:** Elizabeth J. Gross, Sophia G. Barthel, Carter U. Brzezinski, Logan Z. Hessefort, John Bacsa, Kyle F. Biegasiewicz

**Affiliations:** † Department of Chemistry, 1371 Emory University, Atlanta, Georgia 30322, United States; ‡ School of Molecular Sciences, 7864 Arizona State University, Tempe, Arizona 85281, United States

**Keywords:** biocatalysis, chemoenzymatic, chloramine, vanadium-dependent chloroperoxidase

## Abstract

Organic chloramines are an important class of compounds containing a covalent nitrogen–chlorine bond. Despite the growing interest in their applications in small molecule synthesis and polymer science, selective catalyst systems for their preparation have remained elusive. We recently discovered that the vanadium-dependent chloroperoxidase from *Curvularia inaequalis* (*Ci*VCPO) is an effective biocatalyst for selective chlorination of a broad range of structurally diverse amines to give the corresponding chloramines and chlorimines. The catalyst system is readily scalable and applied to chemoenzymatic nitrile and amide synthesis. Finally, halide divergent reactivity is demonstrated through chloride-selective chlorimine formation and bromide-selective aldehyde formation using the same biocatalyst.

## Graphical abstract

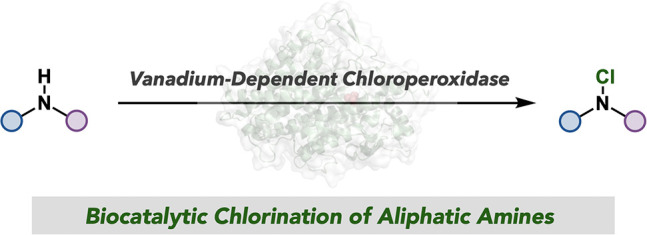

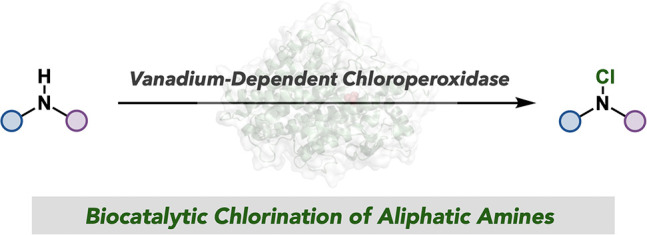

Organic chloramines are a class of compounds containing a covalent nitrogen–chlorine (N–Cl) bond and have been established as valuable targets and reagents across a wide range of chemical industries.^1,2^ Their broad-spectrum antimicrobial activity has made them particularly attractive as additives and functional polymers for healthcare and food safety applications.^3–6^ In society, there is a prevailing interest in accessing nitrogen-containing compounds as pharmaceuticals, agrochemicals, and materials.^7–13^ Traditional methods for their synthesis have included direct alkylation,^14,15^ reductive amination,^16–18^ and cross-coupling reactions,^19–21^ all of which involve the functionalization of amines by exploiting nucleophilic nitrogen reactivity. In contrast, the polarity-reversed nature of the N–Cl bond in chloramines makes them powerful precursors for accessing electrophilic or radical reactivity at the nitrogen center, dramatically expanding the potential scope of amines in chemical synthesis to umpolung (polarity-reversed) oxidation, cross-coupling, cyclization, and rearrangement reaction types.^2,22–24^ Despite the increased interest in using chloramines, current methods for their preparation involve treatment of an amine-containing precursor with stoichiometric quantities of electrophilic chlorinating agents (Cl_2_, TCCA) that suffer from air and moisture sensitivity, generate stoichiometric waste products, and are bio-incompatible, leaving a sustainable catalytic method for amine chlorination highly desired (Figure 1a).

**Figure 1:**
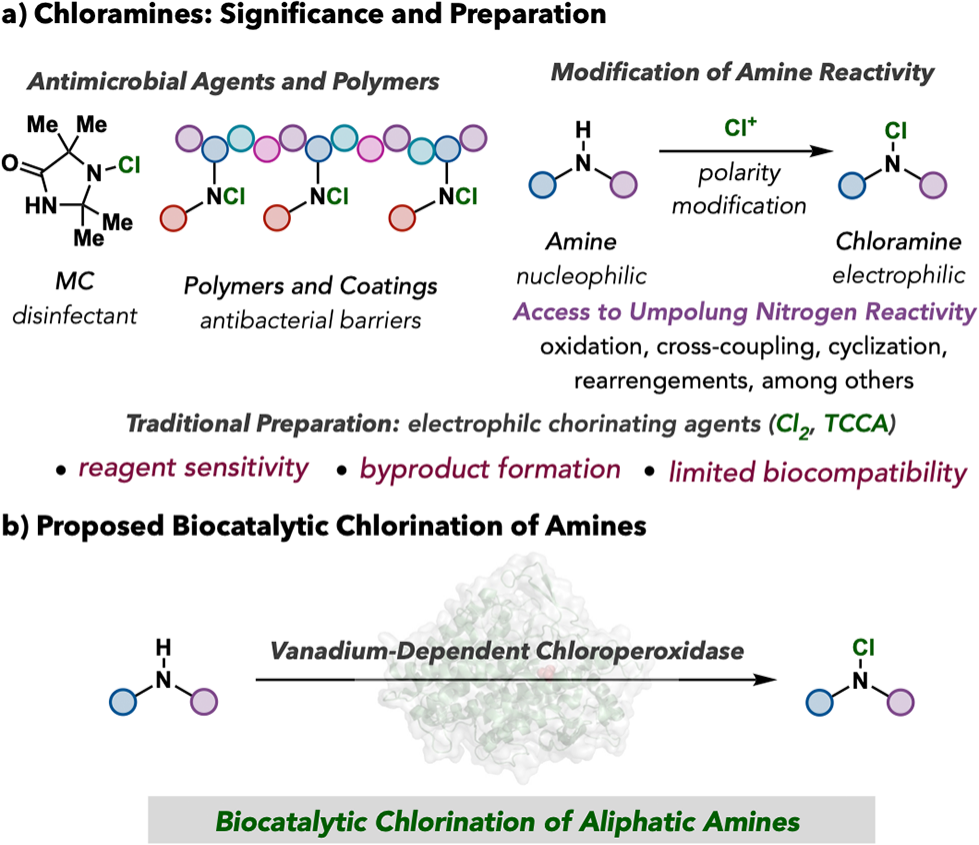
Chloramine significance and proposed biocatalytic chlorination of amines.

Enzymes are an attractive option for catalytic amine chlorination because of their inherent selectivity and sustainability parameters.^25–27^ Despite the intrigue of using enzymes for amine halogenation, nature is devoid of enzymes that have conclusively performed the halogenation of aliphatic amines. While there are select examples of a proposed bromination event in the context of oxidative decarboxylation of amino acids by a vanadium-dependent haloperoxidase (VHPO),^28,29^ there is a significant gap in accessing the comparatively stable class of chloramines for unnatural biosynthesis. Among the growing list of enzymes capable of performing halogenation on organic substrates,^30–33^ the VHPO class of enzymes has emerged as an attractive platform for chemical synthesis because of its broad reaction condition tolerance and operational functionality without the need for an additional cofactor turnover system.^34–37^ We recently discovered that VHPOs are effective catalysts for the chlorination of benzamides and acylbenzamides in a halogenation-mediated synthesis of 1,2,4-oxadiazoles,^38^ but the application of this technology to amines has remained elusive. Recent studies have suggested the formation of a transient chlorinated lysine residue as the selective halogenating agent in bacterial VHPOs from napyradiomycin biosynthesis, highlighting the possibility of using VHPOs for amine chlorination.^39^ Herein, we report that VHPOs are effective catalysts for the chlorination of amines (Figure 1b).

Our investigation began by screening a structurally diverse collection of VHPOs for the conversion of 2-phenylethan-1-amine (**1**) to *N*,*N*-dichloro-2-phenylethan-1-amine (**2**). The chloroperoxidase from *Curvularia inaequalis* (*Ci*VCPO) was chosen as a starting point because of its documented application in a wide range of synthetic applications.^40–46^ Subjection of **1** to *Ci*VCPO (0.0125 mol %), sodium orthovanadate (Na_3_VO_4_, 1 mM), potassium chloride (KCl, 2.2 equiv), and hydrogen peroxide (H_2_O_2_, 2.2 equiv) in potassium phosphate buffer (KPi, 200 mM, pH = 6.5) and acetonitrile (MeCN) as cosolvent (20% v/v) provided dichloramine **2** in 44% yield in 4 h (Figure 2, Entry 1). For comparison to *Ci*VCPO, additional structurally diverse vanadium-dependent haloperoxidases including chloroperoxidases from naphterpin and marinone biosynthesis (MarH1 and MarH3)^47^ and bromoperoxidases from *Corallina officinalis* (*Co*VBPO),^48^ *Corallina pilulifera* (*Cp*VBPO),^49^ and *Acaryochloris marina* (*Am*VBPO)^35^ were interrogated under the same conditions, but this resulted in no detectable product formation (Figure 2, Entries 2–6). Upon simply increasing the KCl loading to 8.0 equiv with *Ci*VCPO, the reaction proceeded in 99% yield (Figure 1, Entry 7). Control reactions were run removing each of the reaction components, including the enzyme, Na_3_VO_4_, KCl, and H_2_O_2_, in turn, resulting in no reactivity and confirming the necessity of all reaction components (Figure 2, Entries 8–11). Some other notable features of the reaction include optimal performance with H_2_O_2_ loadings from 2 to 3 equiv (Figure S4), KCl loadings up to 16 equiv (Figure S5), and KPi buffer in a range of pH 6–6.5 (Figure S6) at 200 mM (Figure S7). The reaction performs well in a broad range of solvents (Figure S8), with an optimal MeCN loading of 20% (v/v) (Figure S9). Finally, the reaction tolerates alternative halide sources (NaCl, LiCl, and NH_4_Cl), but with a notable decrease in performance with NH_4_Cl (Figure S10).

**Figure 2:**
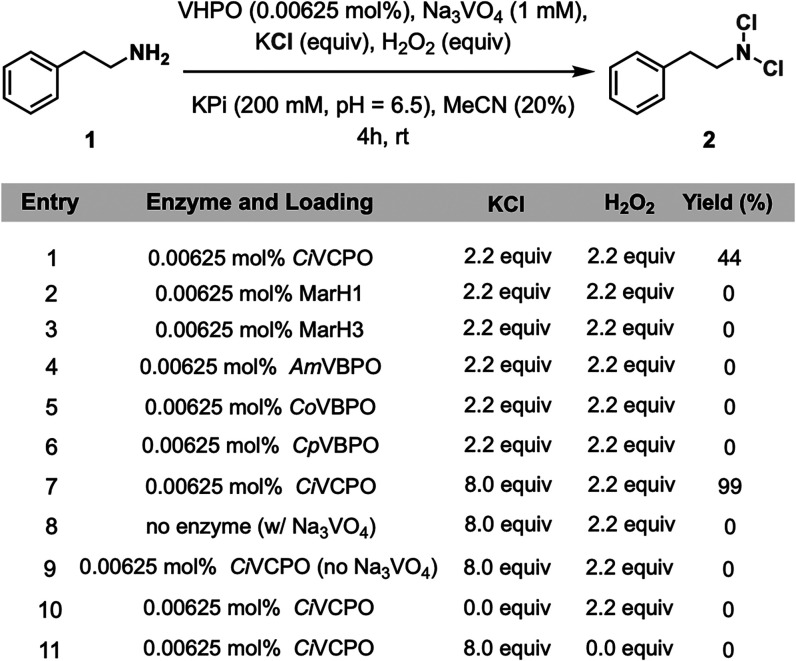
Optimization experiments for biocatalytic chlorination of amines. Reaction conditions: **1** (8.0 μmol, 0.6 mg), VHPO (0.00625 mol %), Na_3_VO_4_ (1 mM), KCl (2.2–8.0 equiv), H_2_O_2_ (2.2 equiv), KPi (200 mM, pH = 6.5, 200 μL), MeCN (200 μL), 1 mL total reaction volume, 4 h, rt. Yields determined by HPLC based on a calibration curve. See Supporting Information for details.

With optimized conditions in hand, a selection of amines were investigated for their performance in VCPO-mediated chlorination. Phenethylamine derivatives with arene substitution at the *para*-position including electron-donating methyl- and methoxy- groups as well as electron-withdrawing chloro- and fluoro- groups were well tolerated with 93–97% yield and with total turnover number (TTN) values of 14848–15520 (Figure 3, **3**–**6**). Aliphatic amines with *meta*-substituents including methoxy-, bromo-, chloro-, and fluoro- groups are accommodated in 89–97% yield and TTNs of 14240–15520 (Figure 3, **7**–**10**). Phenethylamines with *ortho*-substitution including methoxy-, bromo-, and chloro- groups resulted in 91–96% yield and TTNs of 14560–15360 (Figure 3, **11**–**13**). Notably, all phenethylamine derivatives were generated in excellent purity (>99%) without the need for chromatographic purification. To test the effect of chain length, the reaction was performed on 3-phenylpropan-1-amine to give the corresponding dichloramine in 95% yield and a TTN of 15200 (Figure 3, **14**). To test for chemoselectivity over other oxidizable groups, the reaction was successfully performed on 2-aminoalcohol to give the corresponding dichloramine in 74% yield and a TTN of 11840 with no observation of overoxidized products (Figure 3, **15**). A carboxylic acid was also tolerated in the conversion of 4-aminobutanoic acid to the corresponding dichloramine after 24 h in 80% yield and a TTN of 12800 but required acidic workup to isolate as more easily isolable nitrile (Figure 3, **16**). The catalyst system was also effective in the production of monochloramines derived from *N*-methyl-2-phenylethan-1-amine, dibenzylamine, and the anticancer agent, niraparib, in 82–99% yield and 13120–15840 TTN (Figure 3, **17**–**19**) with no byproducts observed. Under slightly modified conditions, monosubstituted benzylamine substrates undergo conversion to the corresponding chlorimines.^46^ This system is effective for the conversion of benzylamine and 4-substituted benzylamines with bromo-, chloro-, and fluoro- substituents to the respective chlorimines in 66–94% yield and TTNs of 10560–15040 (Figure 3, **20**–**23**). Gratifyingly, the TTNs of N-chlorination have been comparable to recent reports using *Ci*VCPO for other halogenation or halogenation-mediated processes.^40,44,50,51^ A comparison of sustainability metrics when compared to chemical methods using TCCA^52^ and Oxone-NaCl^53^ can be found in Supplementary Figure S11.

**Figure 3:**
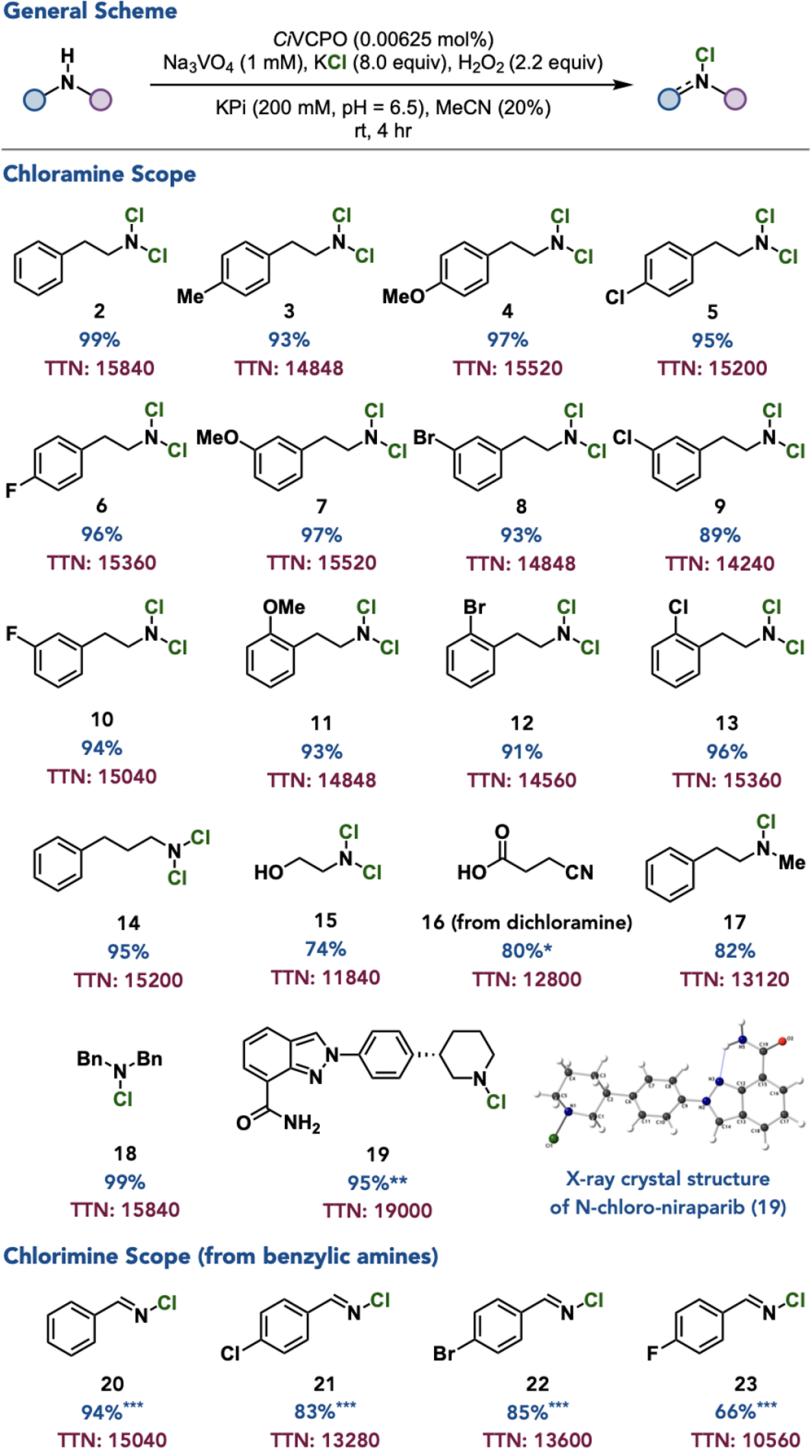
Substrate scope for biocatalytic N-halogenation of amines. Standard reaction conditions: **substrate** (0.8 mmol), *Ci*VCPO (0.00625 mol %), Na_3_VO_4_ (1 mM), KCl (8.0 equiv), H_2_O_2_ (2.0 equiv), KPi (200 mM, pH = 6.5), MeCN (20% v/v), 4 h, rt. *run for 24 h **substrate (0.1 mmol), *Ci*VCPO (0.005 mol %), Na_3_VO_4_ (1 mM), KCl (1.1 equiv), H_2_O_2_ (2.0 equiv), citrate (50 mM, pH = 5), THF (20% v/v), 16 h, rt. ***substrate (0.4 mmol), *Ci*VCPO (0.00625 mol %), Na_3_VO_4_ (1 mM), KCl (2.2 equiv), H_2_O_2_ (2.2 equiv), citrate (100 mM, pH = 5), MeCN (30% v/v), 48 h, rt. Yields determined by isolation. TTNs were determined by dividing the quantity of the resulting product by the concentration of the enzyme used. See the Supporting Information for details.

Intrigued by the production of chlorimines when starting from benzylamine substrates, we were interested in gaining insight into the chloride elimination event. We started by subjecting independently synthesized *N*,*N*-dichlorbenzylamine (**24**) to our standard reaction conditions and controls (no enzyme, no Na_3_VO_4_, no H_2_O_2_, and no KCl). Interestingly, the corresponding chlorimine (**20**) was produced in all reaction conditions in a yield range of 55–95%, suggesting that the elimination event happens in solution rather than through an enzyme-mediated process (Figure 4a). A subsequent time course experiment indicated that, starting from benzylamine (**25**), the corresponding dichloramine (**24**) was produced in a maximum of 70% over the course of 4 h, with subsequent elimination to chlorimine **20** starting in 30 min. Upon maximum generation of **24**, the remainder of the reaction time is dedicated to elimination of chloride in solution to generate **20** in high yield over 48 h (Figure 4b).

**Figure 4:**
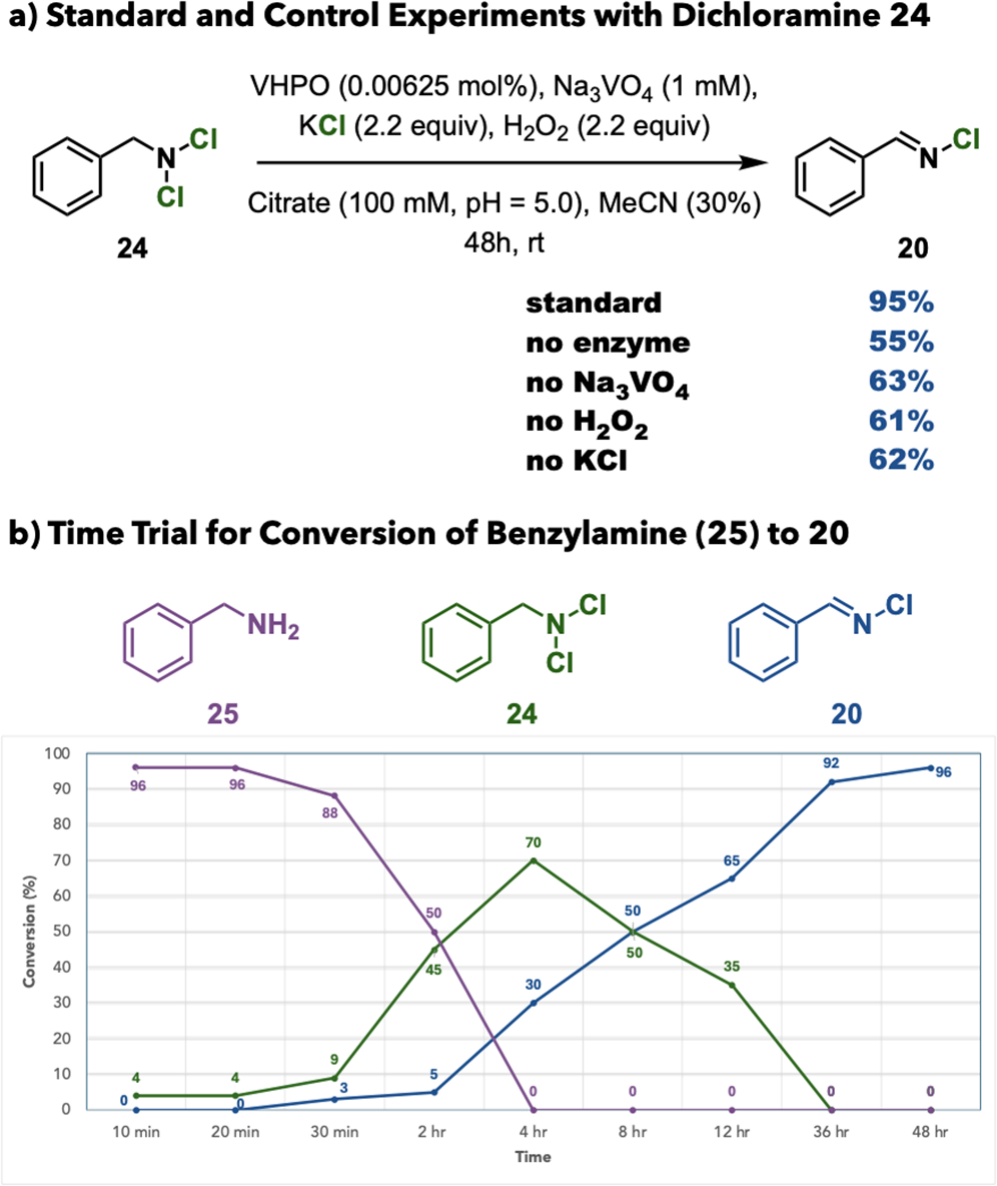
Mechanistic experiments. (a) Standard and control experiments with dichloramine **24**. (b) Time trial for conversion of benzylamine (**25**) to chlorimine **20**.

A proposed mechanism for VCPO-catalyzed chlorination is outlined in Figure 5. Consistent with previously proposed mechanisms for VHPOs,^30–33^ starting from the vanadate cofactor bound to a histidine side chain (**I**), exposure to H_2_O_2_ leads to the displacement of two water molecules to generate peroxovanadium intermediate **II**. Subsequent chloride-mediated opening of peroxovanadium **II** generates a vanadium-bound hypohalite (**III**) that can either participate directly in a halogenation event or be released from the coordination sphere as a hypochlorous acid for a chlorination event. We propose that one of these pathways is responsible for the chlorination of a starting 2° or 1° amine (**IV** or **V**) to give the corresponding monochloramine or dichloramine (**VI** or **VII**), respectively. In the case of benzylic substrates, the elimination of hydrogen chloride (HCl) from dichloramine **IV** results in the formation of the corresponding chlorimine (**VIII**).

**Figure 5:**
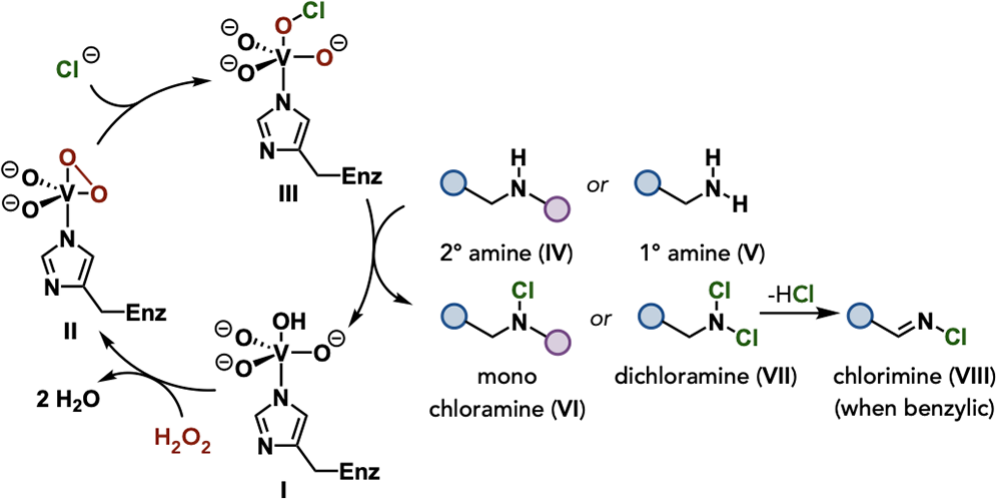
Proposed mechanism for VCPO-mediated chlorination of amines.

Upon completion of the reaction development, the synthetic utility of VCPO-catalyzed chlorination of amines was explored. Amine dichlorination was readily performed on gram-scale with amine **1**, producing dichloramine **2** in 98% yield and a TTN of 15680 (Figure 6a). For further synthetic utility, this protocol was used in the chemoenzymatic synthesis of the corresponding nitrile (**26**) in 99% yield over two steps (Figure 6b).^54^ A similar chemoenzymatic reaction sequence using 1,8-diazabicyclo(5.4.0)­undec-7-ene (DBU) as the base was readily translated to the conversion of benzylamine (**25**) to benzonitrile (**27**) through the intermediacy of chlorimine **20** (Figure 6c). The developed monochlorination conditions were also applicable to the chemoenzymatic synthesis of amides through biocatalytic chlorination of dibenzylamine (**28**) to give monochloramine **18** and subsequent Fe-mediated oxidative coupling to benzaldehyde to provide dibenzylamide **29** in 64% yield over two steps (Figure 6d).^55^ Finally, capitalizing on the ability of VCPOs to oxidize bromide ions,^30–33^ halide divergent activity was explored. Using the same enzyme (*Ci*VCPO), subjection of naphthyl benzylamine **30** to conditions using chloride as the halide provided the corresponding chlorimine **31** in 74% yield and 11840 TTN, while conditions using increased catalyst loading and bromide as the halide provided 2-napthaldehyde **32** in 75% yield and 6000 TTN. Notably, we currently attribute this reaction divergence to the divergent reactivity of the halogenating agent generated (hypochlorous acid or hypobromous acid) rather than a divergent enzyme-mediated process. To the best of our knowledge, this is the first demonstration of halide divergent reactivity by a VHPO, and we envision that these reaction types, in addition to the direct halogenation of amines, can be further enhanced through protein engineering in future works.^56^

**Figure 6:**
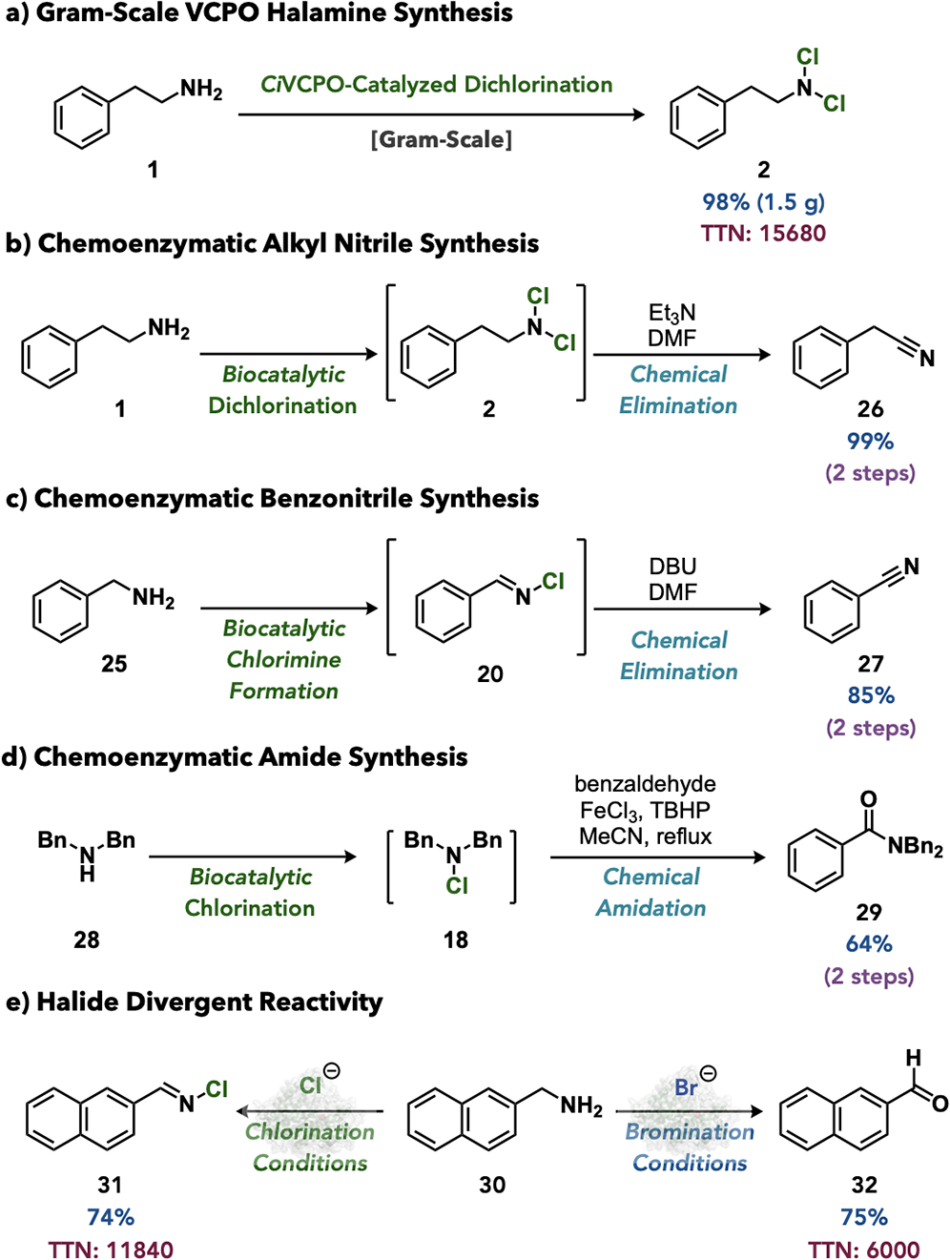
Synthetic application of VCPO-catalyzed halogenation.

In conclusion, *Ci*VCPO is an effective catalyst for the monohalogenation and dihalogenation of amines. These protocols are readily scalable and have been demonstrated in the context of chemoenzymatic syntheses of nitriles and amides. This platform of reactivity can also be used for halide divergent reactivity to produce chlorimines and aldehydes depending on the halide salt employed. These studies not only demonstrate the ability of VHPO enzymes to activate amines but also expand the synthetic application of VHPOs in chemoenzymatic synthesis.

## Supplementary Material



## Notes

Detailed experimental methods, supplemental figures, and characterization data (PDF)
