# Supplementary material for: Chlorination of Amines by a Vanadium-Dependent Chloroperoxidase
Source: ACS Catal. 2026 Mar 23;16(7):6241–6. doi: 10.1021/acscatal.6c00816 (PMC13054786; doi:10.1021/acscatal.6c00816)
Supplement: Supplementary file 1 [file cs6c00816_si_001.pdf]

Supplementary Materials for

Chlorination of Amines by a Vanadium-Dependent Chloroperoxidase

Elizabeth J. Gross[1], Sophia G. Barthel[1], Carter U. Brzezinski[1], Logan Z. Hessefort[2], John Bacsa[1], Kyle F. Biegasiewicz[1,2]*

[1] Department of Chemistry, Emory University, Atlanta, Georgia 30322, USA
[2] School of Molecular Sciences, Arizona State University, Tempe, Arizona 85281, USA

Corresponding author e-mail: kbiegas@emory.edu

This PDF file includes:

Materials and Methods
Product Characterization
Supplementary Text
Figs. S1-S10
Spectral Data
References

Table of Contents

General Experimental Information……………………………………………………..….3
Sequence Information for *Ci*VCPO (PDB: 1VNC)…………………………..…………8
Sequence Information for MarH1……………..………………………..…...……..10
Sequence Information for MarH3……………..………………………..…...……..12
General Procedures for VCPO-Catalyzed N-Chlorination…………………………..….14
Product Characterization for *N,N*-Dichloramines……………………………..…….17
Product Characterization for *N*-Chlorimines……………………………….…………25
Synthetic Utility Protocols………………………………………….………………28
Optimization Data……………………………………………..……………………37
Atom Economy Calculations………………….…...……………………………41
References……………………………………………………………….……….42
Spectroscopic Data………………………………...………………………..…43
Crystallography Information……………………………………………………....71

General Experimental Information

General: Unless specified, all reagents and solvents used in this study were purchased from commercial suppliers and used as received (Combi-Blocks, Sigma-Aldrich, Oakwood Chemicals, Fischer Scientific, VWR). All nonaqueous reactions were performed using glassware that was flame-dried and capped with a rubber septum under nitrogen atmosphere using an inlet and outlet needle connected to a mineral oil bubbler. All aqueous reactions were conducted using glassware without flame-drying prior to experimental set up and without nitrogen atmosphere. Dried or degassed solvent was obtained from a solvent purification system from Pure Process Technology. Unless otherwise indicated, deionized water (H_2O) was used in any experiments where H_2O is included in the procedure. Ultrapure Milli-Q water (Milli-Q H_2O) was accessed through a Milli-Q® EQ 7000 Ultrapure Water Purification System.

Chromatography: Flash chromatography was performed on SiliaFlash® P60 (230-400 mesh, particle size 0.040-0.063 mm) using the solvent systems provided in each procedure. Thin-layer chromatography (TLC) was performed using Uniplate HLF 250 micron F254 precoated glass plates and preparative TLC was performed on Uniplate GF 1000 micron F254 precoated glass plates. For TLC analysis, a short-wave UV lamp and/or plate staining was used.

Spectroscopy and HRMS Anaylsis: ^{1}H- and ^{13}C-NMR spectra were obtained on a Bruker AVIII or Bruker NEO (400 and 101 MHz, respectively). Chemical shifts are reported in ppm (δ) downfield from tetramethylsilane and are internally referenced to the internal deuterated solvent indicated. ^{1}H-NMR data is reported as follows: Chemical Shifts: [multiplicity, coupling constant (Hz), number of hydrogens]. Multiplicities are reported as follows: s (singlet), b (broad signal), d (doublet), dd (doublet of doublets), ddd (doublet of doublet of doublets), t (triplet), dt (doublet of triplets), tt (triplet of triplets), q (quartet), dq (doublet of quartets), p (pentet), m (multiplet). Infrared (IR) spectra were acquired on a Thermo-Fisher Nicolet iS50 spectrometer taken neat, and peaks are reported in frequency of absorption (cm^{-1}). High-resolution mass spectra (HRMS) were obtained on a Thermo Finnigan LTQ-FTMS spectrometer using APCI with an orbitrap mass analyzer.

Analytical: Analytical high-performance liquid chromatography (HPLC) was carried out using a Shimadzu LCMS-2020 System with a Kromasil EternityXT-2.5-C18 column (Dimensions: 4.6x50mm, Batch/Serial: 0000016627/A, Part No. XH2CLA05).

Protein Expression and Purification: All protein expression and purification were performed using previously reported procedures,[1] with the following modified procedure for *Ci*VCPO:

The gene encoding the vanadium-dependent haloperoxidase (VHPO) from *Curvularia inaequalis* (*Ci*VCPO, PDB: 1VNC) was codon-optimized for *E. coli* using the codon optimization tool from Twist Bioscience.[2] The Twist Bioscience platform was then used to insert the gene sequence into a pET-28a(+) expression vector using the insertion sites NdeI and XhoI, which led to the inclusion of an N-terminal 6xHis tag on our *Ci*VCPO enzyme.

The lyophilized plasmid was resuspended in a sterile 10 mM Tris-HCl (pH 8) buffer to a concentration of 20 ng/µL and transformed into BL21(DE3) cells from New England Biolabs using the high-efficiency heat shock transformation method provided by NEB [[https://www.neb.com/en-us/protocols/0001/01/01/transformation-protocol-for-bl21-de3-competent-cells-c2527?srsltid=AfmBOops3k4vBIPbasy5Aej7TycYZLtYkPZ4T7RWJ-fA1rPB8ucpDfnQ]]. A sample of 100 µL of the transformed cells was plated onto an LB agar plate containing 50 mg/L of kanamycin antibiotic and grown overnight (~ 16-18 hours) at 37 °C. A single colony was then picked and used to grow a primary culture (~ 6 mL) in Turbo BrothTM prepared using Milli-Q sterile water and containing 50 mg/L kanamycin in a sterile 10 mL culture tube overnight (~16-18 hours) at 26 °C and 260 rpm in an Infors HT Minitron incubator shaker.

At this stage, 500 µL of the primary culture was used to prepare a 25% glycerol stock (comprising a 1:1 mixture of cell culture and sterile 50% glycerol in water), which was flash-frozen in liquid nitrogen and stored at -80 °C. This cell stock was used to inoculate primary cell cultures in subsequent protein expression and purification batches.

The primary cultures were used to inoculate Kan-Turbo Broth™ 1 L secondary cultures in 3L Fernbach flasks (6 mL of primary culture per 1 L of secondary culture, final kanamycin concentration of 50 mg/L) and the flasks were incubated in an Infors HT Multitron incubator shaker at 37 °C and 200 rpm until the culture reached an optical density (OD600) between 0.8 and 1.0. After reaching the appropriate optical density, the flask was shocked in an ice bath and treated with 100 µL of a 1 M IPTG stock [final concentration of 0.1 mM IPTG]. The liter culture was then allowed to shake overnight (~18-20 hours) at 18 °C, shaken at 200 rpm in an Infors HT Multitron incubator shaker.

The cell cultures were pelleted using centrifugation at 3.5 krpm at 10 °C for 20 minutes in a Beckman Coulter Avanti JXN-26 centrifuge, and the broth supernatant was discarded. The cell pellets from two 1-L cultures were combined into a single sterile 50 mL conical tube, which was then centrifuged at 13,000 rpm at 10 °C for 20 minutes in a Thermo Scientific Sorvall ST Plus Series centrifuge to remove any excess cell broth. These 2-L pellets were stored at -80 °C until further use.

The cell pellets were thawed in lukewarm water and suspended in Nickel-Nitrilotriacetic acid (Ni-NTA) binding buffer containing 50 mM pH 8 Tris-base/H_2SO_4, 50 mM imidazole, 100 mM Na_2SO_4, and 100 mM NaCl. For a 2-L cell pellet in a 50 mL conical tube, the pellet was suspended until a combined volume of 45 mL was reached in the conical tube, and the cells were vortexed until fully homogenized. Once the cells were fully suspended, a 200 mM phenylmethylsulfonyl fluoride (PMSF) stock was added to the suspended pellet solution for a final concentration of 1 mM. The suspended cells were then lysed on ice using a Branson SFX250 Sonifier with a ½ inch probe in 15 second pulses at 32% amplitude, 30 W in 10 bursts with 55 seconds between bursts. The lysed cell solution was then centrifuged at 13,000 rpm at 10 °C for 20 minutes in a Thermo Scientific Sorvall ST Plus Series centrifuge.

After centrifugation, the clarified cell lysate was decanted into a 100 mL glass bottle. To reduce the viscosity and the DNA content of the solution, bovine DNase I was added to the lysate. These DNase stocks were prepared by adding 5.2 mg of DNase and ~17 mg of $MgSO_4$ to 10 mL of 5% glycerol and stored at -20 °C. After thawing at room temperature, 500 µL of this DNase stock was added per ~30 mL of cell lysate. The lysate solution was then placed in an ice bath for 10 minutes.

Ni-NTA chromatography purification was performed on the clarified lysate using a 10 mL (2 x 5 mL linked) Cytiva Life Sciences Ni-NTA column with the GE AKTA Start FPLC system. The Ni- NTA method involved 5 column volume (CV) equilibration with binding buffer, loading of the supernatant onto the column, 13 CV of binding buffer (wash), and then a gradient elution from 0 to 100 % elution buffer containing 50 mM pH 7.8 Tris-base/H_2SO_4 and 500 mM imidazole over 6 CVs. The flow rate was set to 4 mL/min. Fractions were pooled based on the 280 nm absorbance peak they were associated with.

The pooled fractions were prepared for gel electrophoresis, in addition to samples of diluted lysed cell pellet, lysed supernatant, and Ni-NTA column flow-through. The lysed cell pellet was prepared by mixing a portion of centrifuged pellet with 200 µL of BugBuster® Protein Extraction Reagent, vortexing the mixture, centrifuging for 1 minute, and adding 5 µL of the mixture to a clean microcentrifuge tube. The remaining samples were prepared by adding 5 µL of the lysed supernatant, column flowthrough, or pooled fractions to separate microcentrifuge tubes. To each tube was added 5 µL of 4X Laemmli Buffer and 10 µL of 2-mercaptoethanol. These samples were then shaken in an IKA Matrix orbital at 95 ºC and 300 rpm for 15 minutes. Gel electrophoresis was performed on these samples using 10% sodium dodecyl sulfate Mini-PROTEAN TGX Stain-Free hand casted gels (Bio-Rad Laboratories), with an example gel shown in Figure S1. After this visual confirmation via gel electrophoresis, peak fractions containing protein of approximately the same weight as the Expasy Protparam predicted molecular weight (69726.07 g/mol) for *Ci*VCPO were pooled.[3]

The pooled peak fractions containing *Ci*VCPO were desalted using Cytiva desalting (DS) columns on an AKTA Start, loading 1.25 mL sample per 5 mL of DS resin. The desalting method involved 3 column-volume equilibration with *Ci*VCPO's storage buffer (25 mM pH 6 Citrate, 25 mM Na_2SO_4, 2.5 mM $Ca(NO_3)_2$). After fractionation was complete, the fractions having high absorbance at 280 nm, determined from the AKTA chromatogram, were pooled. The absorbances of these combined fractions at 280 nm were determined using a storage buffer-blanked Nanodrop One C. The protein solution was then diluted with storage buffer to a concentration of 10 µM as calculated via the Beer-Lambert law using the Expasy Protparam extinction coefficient.[3] The diluted enzyme was then aliquoted, flash frozen using liquid nitrogen, and stored at -80 °C until further use.

This procedure was repeated for MarH1 (59568.81 g/mol)[3] and MarH3 (58816.17 g/mol)[3] and their respective sequences.

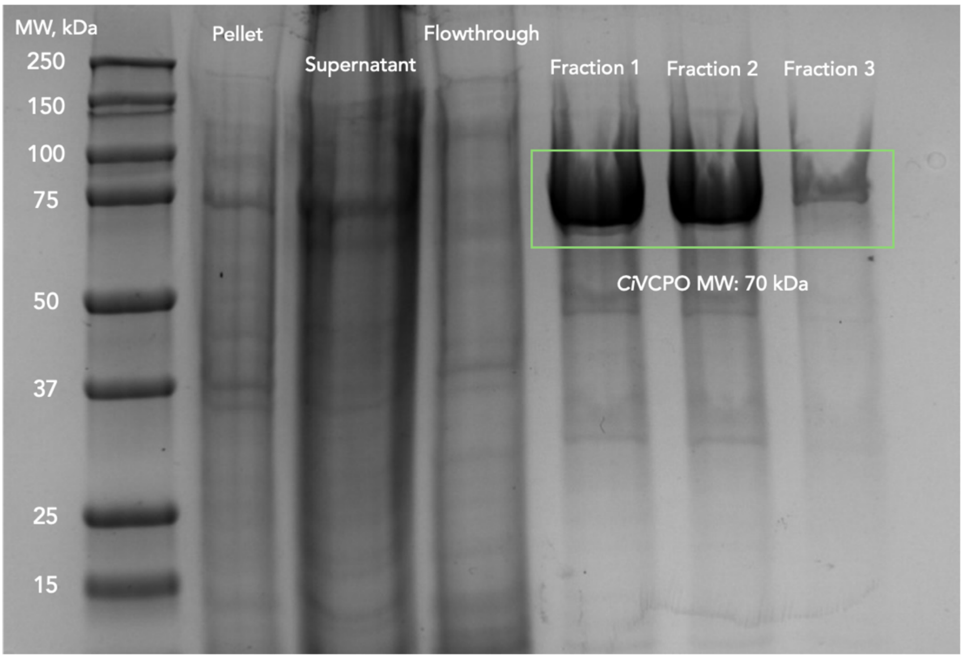

Figure S1. Representative Mini-PROTEAN TGX stain-free hand casted gel after *Ci*VCPO purification (each well contains 10 µL of a four-times diluted sample)

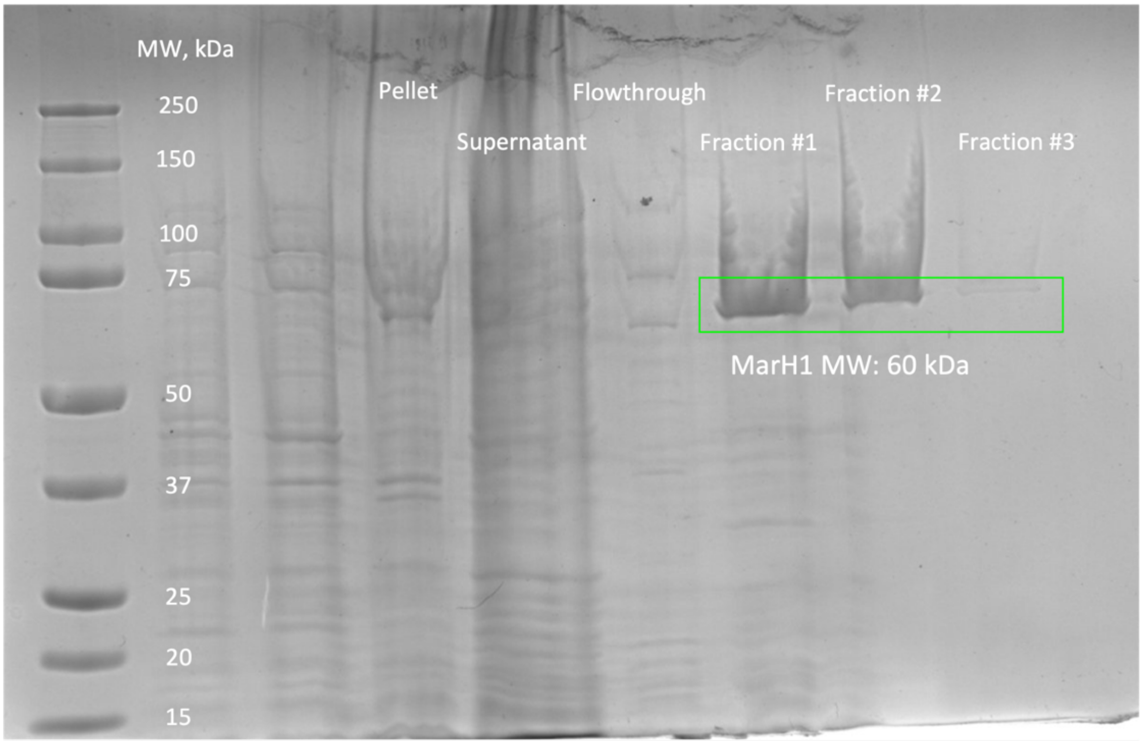

Figure S2. Representative Mini-PROTEAN TGX stain-free hand casted gel after MarH1 purification (each well contains 10 µL of a four-times diluted sample)

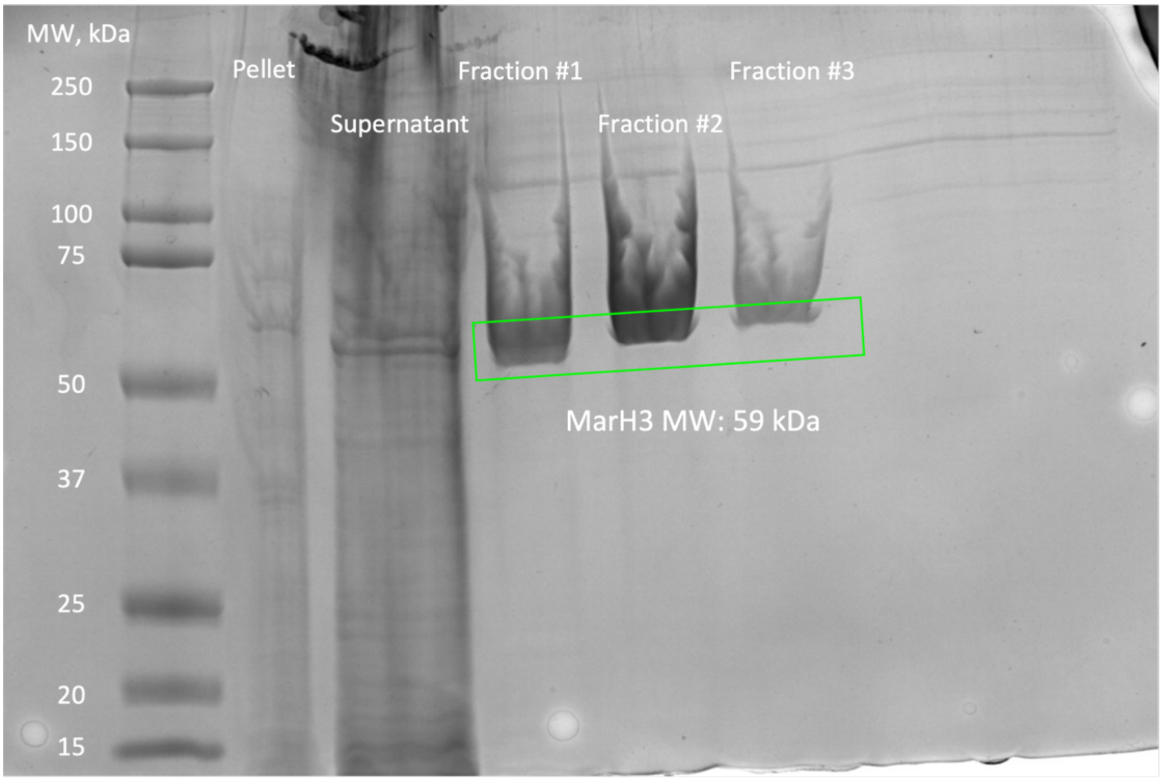

Figure S3. Representative Mini-PROTEAN TGX stain-free hand casted gel after MarH3 purification (each well contains 10 µL of a four-times diluted sample)

<u>Sequence Information for *Ci*VCPO (PDB: 1VNC)</u>

<u>Optimized DNA insert sequence for *Ci*VCPO:</u>

ATGGGGAGCGTGACGCCCATTCCACTGCCGAAAATTGATGAACCGGAAGAATAT
AACACAAACTATATTCTTTTCTGGAACCATGTCGGTTTGGAACTTAACCGAGTCA
CTCATACAGTTGGTGGGCCTCTGACGGGTCCCCCACTCTCGGCTAGAGCCTTAGG
CATGCTGCATCTTGCAATTCATGATGCGTATTTCTCCATTTGCCCACCGACCGATT
TTACCACCTTTCTGAGCCCTGATACCGAAAACGCTGCATATAGACTCCCGTCCCC
GAACGGGGCAAACGATGCGCGGCAGGCTGTTGCGGGCGCTGCTCTGAAAATGTT
GAGTTCGCTTTATATGAAACCGGTTGAACAGCCGAACCCTAACCCCGGGGCTAA
CATTTCTGATAACGCGTATGCCCAGTTGGGCTTAGTTTTAGATCGGTCAGTACTG
GAAGCCCCGGGCGGAGTAGATCGTGAATCGGCGTCTTTTATGTTTGGCGAAGAT
GTCGCCGATGTTTTCTTTGCCCTGCTGAACGATCCTAGAGGGGCGTCCCAGGAAG
GATATCATCCTACGCCGGGGCGCTATAAATTTGATGATGAACCGACGCATCCTGT
TGTGCTGATCCCCGTTGATCCCAACAACCCAAACGGCCCGAAAATGCCATTTCGC
CAGTATCATGCGCCATTTTATGGTAAAACCACCAAACGATTTGCCACACAGTCAG
AACATTTTCTGGCAGACCCGCCGGGCCTTCGTTCGAACGCCGATGAAACGGCTGA
ATATGATGATGCCGTACGCGTAGCGATAGCCATGGGCGGTGCTCAGGCGTTAAA
CAGCACGAAACGGTCTCCATGGCAGACGGCACAGGGACTTTATTGGGCCTATGA
TGGAAGCAACCTGATTGGCACACCACCGCGCTTTTATAACCAGATTGTGCGTCGC
ATTGCAGTCACTTATAAGAAAGAGGAAGATCTGGCCAACTCCGAAGTGAACAAC
GCAGATTTTGCTAGATTGTTTGCGCTGGTGGATGTGGCATGCACCGATGCTGGCA
TATTTAGCTGGAAAGAAAAGTGGGAATTTGAATTTTGGCGCCCTCTGTCCGGGGT
CCGTGATGATGGACGCCCGGATCATGGGGATCCATTTTGGCTGACCCTGGGAGCT
CCGGCGACCAACACTAACGATATTCCGTTTAAACCACCATTTCCGGCTTATCCTA
GTGGTCATGCGACCTTTGGCGGTGCCGTGTTTCAGATGGTGAGACGGTATTATAA
CGGCCGCGTTGGAACATGGAAAGATGATGAACCGGATAACATTGCTATTGATAT
GATGATCTCAGAAGAACTGAACGGAGTGAACCGCGATCTTAGACAGCCATATGA
TCCGACGGCACCGATTGAAGATCAGCCTGGTATTGTCCGGACGAGAATTGTAAG
ACATTTTGATAGTGCTTGGGAACTCATGTTTGAAAACGCAATTAGCCGCATCTTT
CTGGGAGTTCATTGGCGGTTTGATGCGGCAGCTGCGCGCGATATTCTTATTCCGA
CTACTACCAAAGATGTGTATGCGGTAGATAACAACGGCGCTACCGTCTTTCAGAA
CGTTGAAGATATTCGCTATACTACGCGCGGCACTCGCGAAGATGAGGAAGGCCT
CTTTCCTATAGGCGGCGTTCCGCTTGGAATTGAAATTGCAGATGAAATCTTTAAC
AACGGACTGAAACCAACACCGCCTGAAATCCAGCCCATGCCTCAGGAAACCCCG
GTCCAGAAACCGGTCGGACAGCAGCCAGTAAAAGGTATGTGGGAAGAAGAACA
GGCCCCAGTCGTTAAGGAAGCGCCATGA

<u>Insertion Site Name:</u> NdeI_XhoI

<u>Vector Name:</u> pET-28a(+)

Amino Acid Sequence for *Ci*VCPO:

MGSVTPIPLPKIDEPEEYNTNYILFWNHVGLELNRVTHTVGGPLTGPPLSARALGML
HLAIHDAYFSICPPTDFTTFLSPDTENAAYRLPSPNGANDARQAVAGAALKMLSSLY
MKPVEQPNPNPGANISDNAYAQLGLVLDRSVLEAPGGVDRESASFMFGEDVADVFF
ALLNDPRGASQEGYHPTPGRYKFDDEPTHPVVLIPVDPNNPNGPKMPFRQYHAPFYG
KTTKRFATQSEHFLADPPGLRSNADETAEYDDAVRVAIAMGGAQALNSTKRSPWQT
AQGLYWAYDGSNLIGTPPRFYNQIVRRIAVTYKKEEDLANSEVNNADFARLFALVD
VACTDAGIFSWKEKWEFEFWRPLSGVRDDGRPDHGDPFWLTLGAPATNTNDIPFKP
PFPAYPSGHATFGGAVFQMVRRYYNGRVGTWKDDEPDNIAIDMMISEELNGVNRDL
RQPYDPTAPIEDQPGIVRTRIVRHFDSAWELMFENAISRIFLGVHWRFDAAAARDILIP
TTTKDVYAVDNNGATVFQNVEDIRYTTRGTREDEEGLFPIGGVPLGIEIADEIFNNGL
KPTPPEIQPMPQETPVQKPVGQQPVKGMWEEEQAPVVKEAP

Amino Acid Sequence with N-Terminal Tag for *Ci*VCPO:

MGSSHHHHHHSSGLVPRGSHMGSVTPIPLPKIDEPEEYNTNYILFWNHVGLELNRV
THTVGGPLTGPPLSARALGMLHLAIHDAYFSICPPTDFTTFLSPDTENAAYRLPSPNG
ANDARQAVAGAALKMLSSLYMKPVEQPNPNPGANISDNAYAQLGLVLDRSVLEAP
GGVDRESASFMFGEDVADVFFALLNDPRGASQEGYHPTPGRYKFDDEPTHPVVLIPV
DPNNPNGPKMPFRQYHAPFYGKTTKRFATQSEHFLADPPGLRSNADETAEYDDAVR
VAIAMGGAQALNSTKRSPWQTAQGLYWAYDGSNLIGTPPRFYNQIVRRIAVTYKKE
EDLANSEVNNADFARLFALVDVACTDAGIFSWKEKWEFEFWRPLSGVRDDGRPDH
GDPFWLTLGAPATNTNDIPFKPPFPAYPSGHATFGGAVFQMVRRYYNGRVGTWKDD
EPDNIAIDMMISEELNGVNRDLRQPYDPTAPIEDQPGIVRTRIVRHFDSAWELMFENA
ISRIFLGVHWRFDAAAARDILIPTTTKDVYAVDNNGATVFQNVEDIRYTTRGTREDEE
GLFPIGGVPLGIEIADEIFNNGLKPTPPEIQPMPQETPVQKPVGQQPVKGMWEEEQAP
VVKEAP

General Information

Molecular Weight (with N-terminal tag): 69726.07 g/mol

Extinction Coefficient (with N-terminal tag):[3] 91915 M^{-1} cm^{-1}

Protein Storage Buffer: 25 mM Citrate (pH = 6), 25 mM Na_2SO_4, 2.5 mM $Ca(NO_3)_2$

Sequence Information for MarH1:

<u>Optimized DNA insert sequence for MarH1:</u>

ATGGGCAGCAGCCATCATCATCATCATCACAGCAGCGGCCTGGTGCCGCGCGGC
AGCCATATGACGACCGGACACTCTCCTGTCTCAGGCTTCTCCCCGCGGCGCAGGT
CGCTGCTCATCGGCGGTGCCTCCGCCGCCGCGCTGCTCCCCCTGGGTCACGCCGG
CACGGCCGCCGCCGCCGAGGGCGGCAAGGCGGCCCAGGCAGAGTTCGACCTTGA
CAAGGACAACTACATCGAGTGGTTCCAGCCCGAGGACGACGGCGCCGGGATCTC
CCCCTCGTCGGAGATCTTCGGCCCGATGGACGTCACGGTGTTCCTCTGGATCAAC
CACCTGACGGGTCTCGGGTGGTTCGACGCGGTGGCGCCCTACCATGAGACCGCG
GTCGGTGTGCACTCCCGGATTCCCCGCCGTCCGTCCAGCGAGTCCGCCACCAACC
GGAACATGAACATCGCCTGTATCTACTCCCAGTACCAGTTGGTCAAGCAGGTGAT
CCCGAGCCGGGTCAAGCCCATGCGGGACCTGCTGACCAGCATCGGCCTGGACCC
CGACGACGACTCGATGGATCCGGCCGACCCGGTCGGTGTCGGTAACATCGCCGG
CAAGTCCGTCTTCGAGGCCCTCAAGAACGACGGCATGAACTTCCTCGGTCACGAC
GGCGGCCGCAAGTACAACCCCAGGCCCTGGGCGGACTACACCGGCTACCGGCCC
GTGAACACCGCCTTCGACGTCGTCAACCCCTCGCGCTGGCAGCCGCAGCTCCAGG
CCCACAACGGCCGCCGCGTCGGCGGCGGTCCCGGCGACCTGGGCATCTGGGTGG
CCCAGCACTTCGTCACCCCGCAGATGCGGATGGTGAAGCCCCACATCTACGCCG
ACCCGCGCGAGTTCACCGTCCCGCCGCCCAAGCACGTCGACCACACCCGGCCCC
GGGACTTCAAGCGCTCCGCGGACGAGGTCCTGGAGGCGTCGGCCGCGCTCACCG
ACGAGCAGAAGGCCATCGCCGAGGTCATGGACAACAAGATCTGGGGAATCGGCC
ACTCGGCGCTGGTCATCGCGCGGAAGCACGACCAGAACGGCGAGCTGGGCGTGC
AGGGCTGGGCGCACTTCATCCTGGAGCACCTGCTGGCGACGTTCGACCCGCTGAT
CGCCGTCTGGAACGAGAAGACCAAGTACGACGCGGCGCGGCCGGTCACGGTGAT
CCAGCACGTCTACGGCAAGAAGAAGGTGACCTCCTGGGGCGGCCCCGGCATGGG
GACGGTCGACGACATGCCCGCCGGGGAATGGTCCAGCTATCTCCCGGTGGGCGA
CCACCCGGAGTACCCGTCGGGCTCCACGACGCTGTGCTCCGCGGCGTCCCAGTGC
GCGCGGCGCTACTTCGGCTCCGACGAGCTGGACTGGAAGTTCACGTTCCCGGCA
GGCTCGACGCGGACGGAGCCCGGCGTCGTCCCCGCGAAGGACATCGAACTGCAC
TTCCCCACCTGGACCGACTTCACGCAGAAGTGCGGCGCCAGCCGCGTGTGGGGC
GGGGTGCACTTCCGCAAGACGGTCGAGACGTCCATCGCCTTCGGGGAGCAGTTC
GGCGACATGGCCCACGAGTTCGTGCAGAAGCACATCAAGGGTGAGGTCGAGGAC
TGA

<u>Insertion Site Name:</u> NdeI_HindIII

<u>Vector Name:</u> pET-28a(+)

Amino Acid Sequence for MarH1:

MTTGHSPVSGFSPRRRSLLIGGASAAALLPLGHAGTAAAAEGGKAAQAEFDLDKDN
YIEWFQPEDDGAGISPSSEIFGPMDVTVFLWINHLTGLGWFDAVAPYHETAVGVHSR
IPRRPSSESATNRNMNIACIYSQYQLVKQVIPSRVKPMRDLLTSIGLDPDDDSMDPAD
PVGVGNIAGKSVFEALKNDGMNFLGHDGGRKYNPRPWADYTGYRPVNTAFDVVNP
SRWQPQLQAHNGRRVGGGPGDLGIWVAQHFVTPQMRMVKPHIYADPREFTVPPPK
HVDHTRPRDFKRSADEVLEASAALTDEQKAIAEVMDNKIWGIGHSALVIARKHDQN
GELGVQGWAHFILEHLLATFDPLIAVWNEKTKYDAARPVTVIQHVYGKKKVTSWG
GPGMGTVDDMPAGEWSSYLPVGDHPEYPSGSTTLCSAASQCARRYFGSDELDWKFT
FPAGSTRTEPGVVPAKDIELHFPTWTDFTQKCGASRVWGGVHFRKTVETSIAFGEQF
GDMAHEFVQKHIKGEVED

Amino Acid Sequence with N-Terminal Tag for MarH1:

MGSSHHHHHHSSGLVPRGSHMTTGHSPVSGFSPRRRSLLIGGASAAALLPLGHAGT
AAAAEGGKAAQAEFDLDKDNYIEWFQPEDDGAGISPSSEIFGPMDVTVFLWINHLTG
LGWFDAVAPYHETAVGVHSRIPRRPSSESATNRNMNIACIYSQYQLVKQVIPSRVKP
MRDLLTSIGLDPDDDSMDPADPVGVGNIAGKSVFEALKNDGMNFLGHDGGRKYNP
RPWADYTGYRPVNTAFDVVNPSRWQPQLQAHNGRRVGGGPGDLGIWVAQHFVTP
QMRMVKPHIYADPREFTVPPPKHVDHTRPRDFKRSADEVLEASAALTDEQKAIAEV
MDNKIWGIGHSALVIARKHDQNGELGVQGWAHFILEHLLATFDPLIAVWNEKTKYD
AARPVTVIQHVYGKKKVTSWGGPGMGTVDDMPAGEWSSYLPVGDHPEYPSGSTTL
CSAASQCARRYFGSDELDWKFTFPAGSTRTEPGVVPAKDIELHFPTWTDFTQKCGAS
RVWGGVHFRKTVETSIAFGEQFGDMAHEFVQKHIKGEVED

General Information

Molecular Weight (with N-terminal tag): 59568.81 g/mol

Extinction Coefficient (with N-terminal tag):[3] 96620 M^{-1} cm^{-1}

Protein Storage Buffer: 25 mM HEPES, pH 7.6, 25 mM Na_2SO_4, 2.5 mM $Ca(NO_3)_2$

Sequence Information for MarH3:

Optimized DNA insert sequence for MarH3:

ATGGGCAGCAGCCATCATCATCATCATCACAGCAGCGGCCTGGTGCCGCGCGGC
AGCCATATGACGTCCGGAAACTCCTCCTCCGCTGGCTTCTCCCCAGCTCGCAGGT
CGCTGCTGCTCGGCGGCGCCTCGACCGCCGCGCTGGCGACCCTGGGGACCGGCA
CCGCGGCCGCCGCCGGCCAGGGCACGGGACCGGCGGCGAAGCCGGCCGCCGCC
GCCGAATTCGACTTCGACACCGGCAACTTCCACCGGGACCTGCTCAGCACGGCG
GCCAACCCGTCCGAGGAGCCGCTCGGCCCGATGGACGCGACGGTCCTCGTCTAC
CTCACCCATCTCACGATGACCGCGTGGTTCGACGCGCTGGCGCCCTACCACCCGA
CCGCCGTCGGCCGGCACAGCCGCATACCGCGCCGTCCCGCCAGCGAGTCGAACA
CCAACCGGAACAAGAACGTCGCCGCGTTCCACGCCGCGAACGTGGTGTTCCAGT
CCGTCTTCAAGGAGCGGGTGGCGGCCTTCCGGCAGCTCATGACCACGCTCGGCCT
GGACCCCGACGACCAGTCGACGGATCCCACCAGCCCGGTGGGCATCGGCAACCT
GGCCGCCAGGGGCGTCCTCAAGGCCAAGGCGCGTGACGGCATGAACCTCTTCGG
CGACGAGGGCCGGAAGTACCACGGGCAGCCCTACGAGGACTACACCGGCTTCGA
GCCGGTGAACACCGCCTACAAGCTGGTCAACCCGTCGAAGTGGCAGCCGGCCAG
GCACCCGCACCGCCGCCGGGTGGGCGGCGGCCCGGGCGACAAGGGCATCTTCGT
CGTCCAGCACATGGTCACCCCGCAGATGGCGCTGGTGAAGCCGGACACCTACCG
GCACCCGAGCCAGTTCCCGCTGGCCCCGCCGGACCACCTCGACCACACCGACCC
GCGCCGCTACAAGCGCTCGGTGGACGAGATCCTGGAGGCGTCCGCCACGCTCGA
CGACGAGCAGAAGGTGAAGGCCGAGTTCTTCGACAACAAGTTCCTGGGCATCGG
CCAGTCGACGAAGGCCGCGGGGATAGCCCACGACCTGGAGCTGGACGACTGGGT
CCACCTGATCTACACGAGTTCGCTGGCACAGGTCGAGGATCTCATCGCGGCCTGG
CACTATAAGGTCAAGTACCAGGCGCCGCGGCCGTTCTCCGCCATCCGGCACGTGT
ACGGCAAGAAGAAGGTGTCCGCCTGGGGCGGGCCCGGCATCGGGACCGTGCACG
ACATGCCCGCCGACGAGTGGGCCAGCTTCCTCCCCGTGGGCGACCACCCCGACT
ACCCGTCGGGATCCACGACGCTCTGCGCCGCCGAGGCGCAGGCCGCCCGGCGCT
TCCTCGGCGACGACCGCCTGGACTGGACCTGGCCCATCCCCGCCGGCTGGACGCT
GACGGAGCCCGGGATCACCCCCGCCAGGGACATGGAGCTGCACTACGGCACCTG
GACCGAGTTCGTGAAGGACTGCGGCTACAGCCGGCTCTGGGCCGGGGTGCACTT
CACGACCACGGTCGAGCGGTCGATGGAGTTCGGCACCCAGTTCGGCGACCTCGC
GTACGAGTTCGCCCAGAAGTACATCAAGGGCGACGTGGAGGACTGA

Insertion Site Name: NdeI_HindIII

Vector Name: pET-28a(+)

Amino Acid Sequence for MarH3:

MTSGNSSSAGFSPARRSLLLGGASTAALATLGTGTAAAAGQGTGPAAKPAAAAEFD
FDTGNFHRDLLSTAANPSEEPLGPMDATVLVYLTHLTMTAWFDALAPYHPTAVGRH
SRIPRRPASESNTNRNKNVAAFHAANVVFQSVFKERVAAFRQLMTTLGLDPDDQST
DPTSPVGIGNLAARGVLKAKARDGMNLFGDEGRKYHGQPYEDYTGFEPVNTAYKL
VNPSKWQPARHPHRRRVGGGPGDKGIFVVQHMVTPQMALVKPDTYRHPSQFPLAPP
DHLDHTDPRRYKRSVDEILEASATLDDEQKVKAEFFDNKFLGIGQSTKAAGIAHDLE
LDDWVHLIYTSSLAQVEDLIAAWHYKVKYQAPRPFSAIRHVYGKKKVSAWGGPGIG
TVHDMPADEWASFLPVGDHPDYPSGSTTLCAAEAQAARRFLGDDRLDWTWPIPAG
WTLTEPGITPARDMELHYGTWTEFVKDCGYSRLWAGVHFTTTVERSMEFGTQFGDL
AYEFAQKYIKGDVED

Amino Acid Sequence with N-Terminal Tag for MarH3:

MGSSHHHHHHSSGLVPRGSHMTSGNSSSAGFSPARRSLLLGGASTAALATLGTGT
AAAAGQGTGPAAKPAAAAEFDFDTGNFHRDLLSTAANPSEEPLGPMDATVLVYLTH
LTMTAWFDALAPYHPTAVGRHSRIPRRPASESNTNRNKNVAAFHAANVVFQSVFKE
RVAAFRQLMTTLGLDPDDQSTDPTSPVGIGNLAARGVLKAKARDGMNLFGDEGRK
YHGQPYEDYTGFEPVNTAYKLVNPSKWQPARHPHRRRVGGGPGDKGIFVVQHMVT
PQMALVKPDTYRHPSQFPLAPPDHLDHTDPRRYKRSVDEILEASATLDDEQKVKAEF
FDNKFLGIGQSTKAAGIAHDLELDDWVHLIYTSSLAQVEDLIAAWHYKVKYQAPRPF
SAIRHVYGKKKVSAWGGPGIGTVHDMPADEWASFLPVGDHPDYPSGSTTLCAAEAQ
AARRFLGDDRLDWTWPIPAGWTLTEPGITPARDMELHYGTWTEFVKDCGYSRLWA
GVHFTTTVERSMEFGTQFGDLAYEFAQKYIKGDVED

General Information

Molecular Weight (with N-terminal tag): 58816.17 g/mol

Extinction Coefficient (with N-terminal tag):[3] 85955 M^{-1} cm^{-1}

Protein Storage Buffer: 25 mM HEPES, pH 7.6, 25 mM Na_2SO_4, 2.5 mM $Ca(NO_3)_2$

General Procedures for VCPO-Catalyzed Chlorination of Amines

General Analytical Procedure for the Synthesis of Chloramines (General Procedure A):

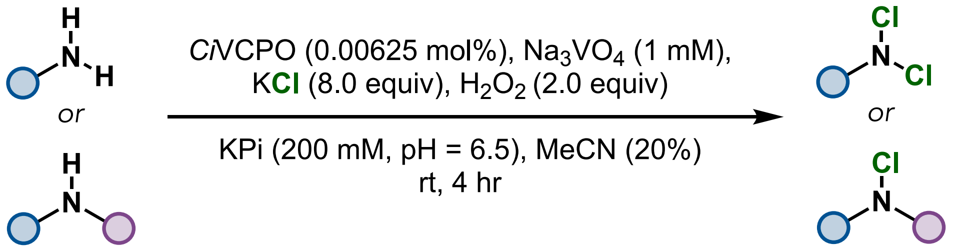

An enzyme aliquot of purified VHPO from *Curvularia inaequalis* (*Ci*VCPO, 10 µM, 50 µL) is removed from a -80 °C freezer and allowed to warm to room temperature for 5 minutes. Once thawed, the enzyme is subjected to a 10-second centrifugation using a Chemglass Life Sciences MLX-108-CLS Mini Centrifuge to combine the thawed enzyme solution to the bottom of the PCR tube. An aqueous solution of 250 mM Na_3VO_4 (4 uL) is added to the aliquot and the resulting solution is microcentrifuged again for 10 seconds. This enzyme-vanadate aliquot is allowed to sit at room temperature for a 20-minute incubation period. A 1-dram vial is then charged with purified Milli-Q H_2O (476 µL) and followed by 1 M pH 6.5 KPi buffer (200 µL) and 1 M KCl (64 µL, 8.0 equiv). A 40 mM solution of the starting amine in MeCN (200 µL, 1 equiv, 0.008 mmol substrate) is then added. The aliquot containing the diluted *Ci*VCPO (0.00625 mol%, 0.5 µM) and Na_3VO_4 (0.125 equiv) is added to the reaction mixture. Finally, a 10% stock of H_2O_2 (5.8 µL, 2.2 equiv) is added to the reaction mixture. The vial is capped and placed on a Heidolph Multi Reax shaker set to 820 rpm at room temperature for 4 hours. After this time, the reaction mixture is diluted with MeCN (650 µL), transferred to an Eppendorf tube, and spun down in a Benchmark MC-24TM Touch Centrifuge at 12,500 rpm for 5 min. After centrifugation, 500 µL of the top layer of the reaction mixture is transferred to an LC-MS vial and then placed on an LC-MS for analysis. Where applicable, a standard curve is used with 1,3,5-tribromobenzene as the internal standard (8 mg/mL) to determine yields.

General Preparative Procedure for the Synthesis of *N,N*-Dichloramines (General Procedure B):

A Falcon® tube containing purified VHPO from *Curvularia inaequalis* (*Ci*VCPO, 10 µM, 5 mL) is removed from a -80 °C freezer and allowed to warm to room temperature over 10 min. An aqueous solution of 250 mM Na_3VO_4 (400 µL) is added to the thawed enzyme, and the enzyme-vanadate solution is allowed to sit at room temperature for a 20-minute incubation period. A 250 mL round bottom flask is charged with 0.8 mmol of the starting substrate. After the addition of a teflon stir bar, MeCN (20 mL) is added. Next, Milli-Q H_2O (18 mL), 1 M pH 6.5 KPi buffer (20 mL), and 176 mM KCl (36.4 mL, 8.0 equiv) are added to the MeCN solution. After the 20-minute incubation of the enzyme-vanadate solution at room temperature, the Falcon® tube containing *Ci*VCPO (0.00625 mol%, 0.5 µM) and Na_3VO_4 (0.125 equiv) is added to the reaction mixture. A 30% stock of H_2O_2 (180 µL, 2.2 equiv) is added to the reaction

mixture and the resulting solution is allowed to stir at room temperature for 4 hours unless otherwise noted. After this time, the reaction mixture is quenched with ethyl acetate (50 mL) and transferred to a 250 mL separatory funnel. The aqueous layer is extracted with ethyl acetate (2 x 125 mL) and the combined organic layers are washed with H_2O (2 x 10 mL), brine (2 x 5 mL) and dried over sodium sulfate (Na_2SO_4) for 10 minutes. The dried organic layer was then decanted into a round bottom flask. To ensure adequate transfer, the Na_2SO_4 was rinsed with ethyl acetate and decanted into the round bottom flask three times. The product was then concentrated down without need for further purification unless otherwise stated.

General Analytical Procedure for the Synthesis of Chlorimines (General Procedure C):

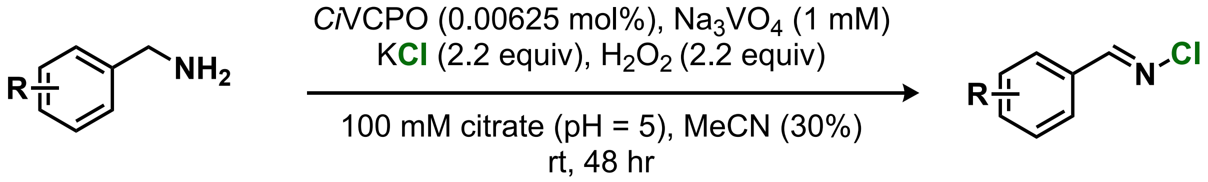

An enzyme aliquot of purified VHPO from *Curvularia inaequalis* (*Ci*VCPO, 10 µM, 25 µL) is removed from a -80 °C freezer and allowed to warm to room temperature for 5 minutes. Once thawed, it is subjected to a 10-second centrifugation using a Chemglass Life Sciences MLX-108-CLS Mini Centrifuge to combine the thawed enzyme solution to the bottom of the PCR tube. An aqueous solution of 250 mM Na_3VO_4 (4 uL) is added to the aliquot and the resulting solution is spun again in the mini microcentrifuge for 10 seconds. This enzyme-vanadate aliquot is allowed to sit at room temperature for a 20-minute incubation period, allowing the enzyme and cofactor to mix in solution. A 1-dram vial is then charged with Milli-Q H_2O (532 µL) and followed by 1 M pH 5 citrate buffer (100 µL), 176 mM KCl (50 µL, 2.2 equiv), and MeCN (200 µL). A 40 mM solution of the starting amine in MeCN (100 µL, 1 equiv, 0.004 mmol substrate) is then added. The aliquot containing the diluted *Ci*VCPO (0.00625 mol%, 0.25 µM) and Na_3VO_4 (0.25 equiv) is added to the reaction mixture. Finally, a 10% stock of H_2O_2 (2.9 µL, 2.2 equiv) is added to the reaction mixture. The vial is capped and placed on a Heidolph Multi Reax shaker set to 820 rpm at room temperature for 4 hours. After this time, the reaction mixture is diluted with MeCN (650 µL), transferred to an Eppendorf tube, and spun down in a Benchmark MC-24TM Touch Centrifuge at 12,500 rpm for 5 min. After centrifugation, 500 µL of the top layer of the reaction mixture is transferred to an LC-MS vial and then placed on an LC-MS for analysis. Where applicable, a standard curve is used with 1,3,5-tribromobenzene as the internal standard (8 mg/mL) to determine yields.

General Preparative Procedure for the Synthesis of Chlorimines (General Procedure D):

A Falcon® tube containing purified VHPO from *Curvularia inaequalis* (*Ci*VCPO, 10 µM, 2.5 mL) is removed from a -80 °C freezer and allowed to warm to room temperature over 10 min. An aqueous solution of 250 mM Na_3VO_4 (400 µL) is added to the thawed enzyme, and the enzyme-vanadate solution is allowed to sit at room temperature for a 20-minute incubation period. A 250 mL round bottom flask is charged with 0.4 mmol of the starting substrate. After addition of a teflon stir bar, MeCN (30 mL) is added. Next, Milli-Q H_2O (52 mL), 1 M pH 5

citrate buffer (10 mL), and 176 mM KCl (5 mL, 2.2 equiv) are added to the MeCN solution. After the 20-minute incubation of the enzyme-vanadate solution at room temperature, the Falcon® tube containing the *Ci*VCPO (0.00625 mol%, 0.25 μM) and Na_3VO_4 (0.25 equiv) is added to the reaction mixture. A 30% stock of H_2O_2 (90 μL, 2.2 equiv) is then added to the reaction mixture and the reaction is allowed to stir at room temperature for 48 hours. After this time, the reaction mixture is quenched with ethyl acetate (50 mL) and transferred to a 250 mL separatory funnel. The aqueous layer is extracted with ethyl acetate (3 x 125 mL) and the combined organic layers are then washed with H_2O (1 x 10 mL), brine (1 x 5 mL) and dried over Na_2SO_4 for 10 minutes. To ensure adequate transfer, the Na_2SO_4 was rinsed with ethyl acetate and decanted into the round bottom flask three times. The product was then concentrated down and purified via column chromatography.

Product Characterization for *N,N*-Dichloramines

N,N-dichloro-2-phenylethan-1-amine (2)

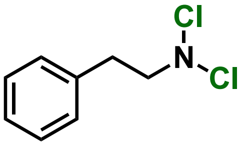

Synthesized from commercially available phenethylamine (CAS 64-04-0) following General Procedure B.

Yield: 99% (yellow oil)

^{1}H NMR (400 MHz, $CDCl_3$) δ 7.40 – 7.16 (m, 5H), 4.01 – 3.78 (m, 2H), 3.07 (t, J = 7.5 Hz, 2H).

^{13}C NMR (126 MHz, $CDCl_3$) δ 137.19, 129.00, 128.84, 126.94, 76.71, 34.93.

These spectra are consistent with previously reported values.[4]

Standard Curve for Analytical Run

Procedure for using all standard curves is as follows: 1,3,5-tribromobenzene (8 mg/mL solution, 100 µL) is added to 600 µL of the reaction mixture and yield is determined by LCMS analysis based on the below standard curve. LCMS conditions: 2.5 µL injection volume, 0.5 mL/min mobile phase rate, 10-98% solvent B over 6.25 min. Mobile Phase: Solvent A – H2O w/ 0.1% formic acid, Solvent B – MeCN w/ 0.1% formic acid.

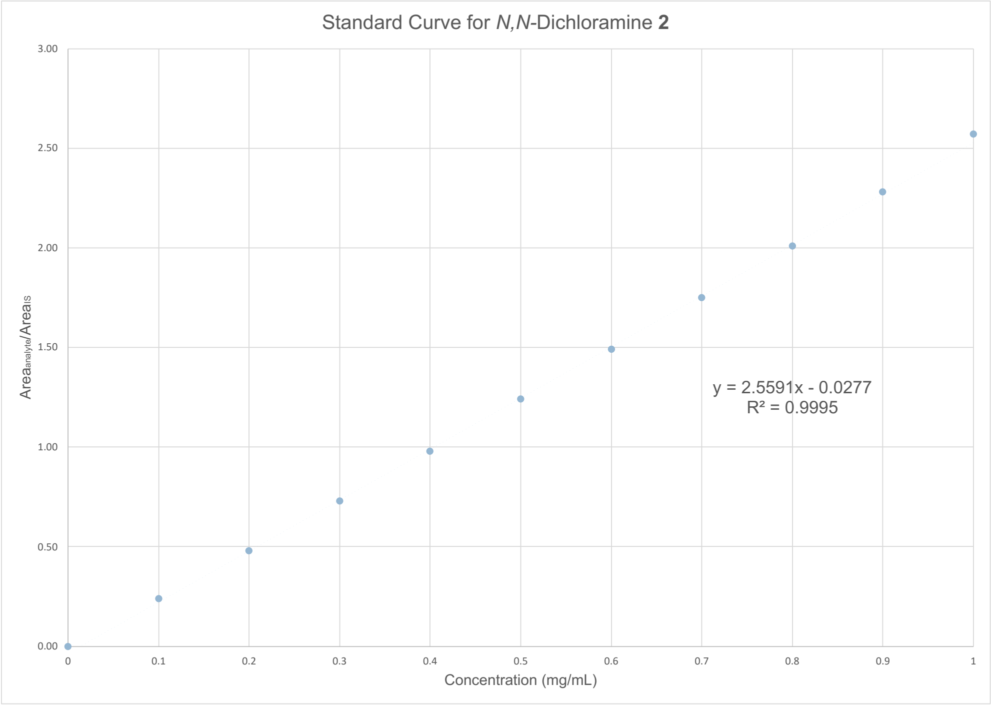

***N,N*-dichloro-2-(p-tolyl)ethan-1-amine (3)**

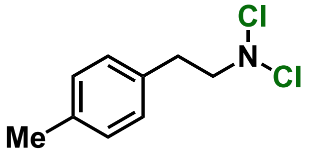

Synthesized from commercially available 4-methylphenethylamine (CAS: 3261-62-9) following General Procedure B.

Yield: 93% (yellow oil)

^{1}H NMR (500 MHz, $CDCl_3$) δ 7.12 (d, J = 3.4 Hz, 4H), 3.91 – 3.75 (m, 2H), 3.08 – 2.94 (m, 2H), 2.34 (s, 3H).

^{13}C NMR (126 MHz, $CDCl_3$) δ 136.52, 134.05, 129.52, 128.87, 76.87, 34.50, 21.19.

HRMS (APCI) m/z: calculated for $C_9H_{12}NCl_2$ $[M+H^+]^+$: 204.0341. Found $[M]^+$: 204.0343.

IR: (cm^{-1}) 2919.49, 1513.92, 808.30

***N,N*-dichloro-2-(4-methoxyphenyl)ethan-1-amine (4)**

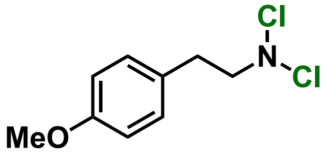

Synthesized from commercially available 2-(4-methoxyphenyl)ethylamine (CAS: 55-81-2) following General Procedure B.

Yield: 97% (yellow oil)

^{1}H NMR (500 MHz, $CDCl_3$) δ 7.14 (d, J = 8.7 Hz, 2H), 6.86 (d, J = 8.6 Hz, 2H), 3.92 – 3.75 (m, 5H), 3.00 (t, J = 7.5 Hz, 2H).

^{13}C NMR (126 MHz, $CDCl_3$) δ 158.58, 129.97, 129.14, 114.24, 76.99, 55.41, 34.08.

HRMS (APCI) m/z: calculated for $C_9H_{12}ONCl_2$ $[M+H^+]^+$: 220.0291. Found $[M+H^+]^+$: 220.0293.

IR: (cm^{-1}) 2932.20, 2833.79, 1510.49, 1243.07, 822.20

_N,N_-dichloro-2-(4-chlorophenyl)ethan-1- (5)

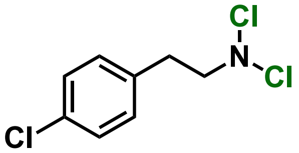

Synthesized from commercially available 2-(4-chlorophenyl)ethylamine (CAS: 156-41-2) following General Procedure B.

Yield: 95% (yellow oil)

^{1}H NMR (500 MHz, $CDCl_3$) δ 7.29 (d, J = 8.4 Hz, 2H), 7.15 (d, J = 8.4 Hz, 2H), 4.01 – 3.69 (m, 2H), 3.25 – 2.90 (m, 2H).

^{13}C NMR (101 MHz, $CDCl_3$) δ 135.64, 132.79, 130.34, 128.96, 76.31, 34.24.

These spectra are consistent with previously reported values.[5]

_N,N_-dichloro-2-(4-fluorophenyl)ethan-1-amine (6)

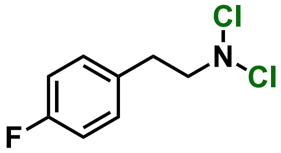

Synthesized from commercially available 2-(4-fluoro-phenyl)-ethylamine (CAS: 1583-88-6) following General Procedure B.

Yield: 96% (yellow oil)

^{1}H NMR (500 MHz, $CDCl_3$) δ 7.22 – 7.13 (m, 2H), 7.00 (t, J = 8.7 Hz, 2H), 3.83 (t, J = 7.4 Hz, 2H), 3.03 (t, J = 7.4 Hz, 2H).

^{13}C NMR (101 MHz, $CDCl_3$) δ 161.91 (d, J = 245.0 Hz), 132.86, 130.45 (d, J = 7.7 Hz), 115.67 (d, J = 21.6 Hz), 76.61, 34.11.

HRMS (APCI) m/z: calculated for $C_9H_9NCl_2F$ $[M+H^+]^+$: 208.0091. Found $[M+H^+]^+$: 208.0094.

IR: (cm^{-1}) 2919.73, 1507.99, 1220.67, 824.65

N,N-dichloro-2-(3-methoxyphenyl)ethan-1-amine (7)

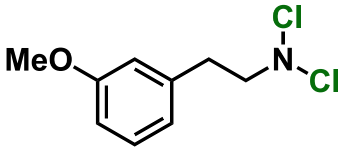

Synthesized from commercially available 2-(3-methoxyphenyl)ethylamine (CAS: 2039-67-0) following General Procedure B.

Yield: 97% (yellow oil)

^{1}H NMR (400 MHz, $CDCl_3$) δ 7.23 (d, J = 8.1 Hz, 1H), 6.79 (dd, J = 16.3, 8.1 Hz, 3H), 3.90 – 3.82 (m, 2H), 3.81 (s, 3H), 3.11 – 2.99 (m, 2H).

^{13}C NMR (126 MHz, $CDCl_3$) δ 159.92, 138.73, 129.83, 121.29, 114.81, 112.14, 76.59, 55.33, 34.94.

HRMS (APCI) m/z: calculated for $C_9H_{12}ONCl_2$ $[M+H^+]^+$: 220.0291. Found $[M+H^+]^+$: 220.0293.

IR: (cm^{-1}) 2936.94, 2833.78, 1225.80, 1165.96, 776.47, 692.90

2-(3-bromophenyl)-N,N-dichloroethan-1-amine (8)

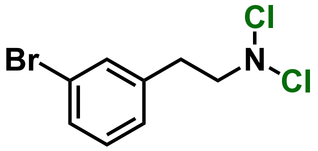

Synthesized from commercially available 2-(3-bromo-phenyl)-ethylamine (CAS: 58971-11-2) following General Procedure B.

Yield: 93% (yellow oil)

^{1}H NMR (500 MHz, $CDCl_3$) δ 7.42 – 7.34 (m, 2H), 7.22 – 7.13 (m, 2H), 4.03 – 3.71 (m, 2H), 3.21 – 2.89 (m, 2H).

^{13}C NMR (126 MHz, $CDCl_3$) δ 139.49, 132.03, 130.38, 130.12, 127.68, 122.81, 76.13, 34.47.

HRMS: calculated for $C_8H_8NBrCl_2$ $[M]^+$: 266.9223. Found $[M]^+$: 266.9213.

IR: (cm^{-1}) 2919.15, 806.99, 777.38, 699.44

N,N-dichloro-2-(3-chlorophenyl)ethan-1-amine (9)

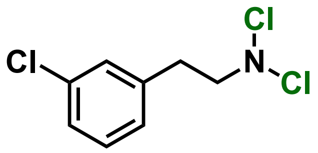

Synthesized from commercially available 3-chlorophenethylamine (CAS: 13078-79-0) following General Procedure B.

Yield: 89% (yellow oil)

^{1}H NMR (500 MHz, $CDCl_3$) δ 7.31 – 7.19 (m, 3H), 7.10 (dt, J = 6.6, 2.1 Hz, 1H), 3.97 – 3.61 (m, 2H), 3.25 – 2.89 (m, 2H).

^{13}C NMR (126 MHz, $CDCl_3$) δ 139.18, 134.56, 130.08, 129.12, 127.20, 127.19, 76.13, 34.50.

HRMS (APCI) m/z: calculated for $C_8H_8NCl_3$ $[M]^+$: 222.9717. Found $[M]^+$: 222.9719.

IR: (cm^{-1}) 2919.25, 779.60, 709.42, 685.84

N,N-dichloro-2-(3-fluorophenyl)ethan-1-amine (10)

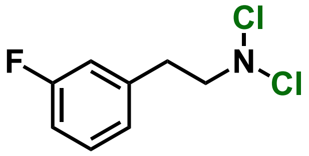

Synthesized from commercially available 2-(3-fluoro-phenyl)-ethylamine (CAS: 404-70-6) following General Procedure B.

Yield: 94% (yellow oil)

^{1}H NMR (400 MHz, $CDCl_3$) δ 7.38 – 7.26 (m, 1H), 6.96 (dd, J = 25.4, 8.0 Hz, 3H), 3.85 (t, J = 7.3 Hz, 2H), 3.06 (t, J = 7.3 Hz, 2H).

^{13}C NMR (101 MHz, $CDCl_3$) δ 163.05 (d, J = 246.1 Hz), 139.69, 130.34, 124.66, 115.83, 114.02, 76.16, 34.59.

HRMS (APCI) m/z: calculated for $C_9H_9NCl_2F$ $[M+H^+]^+$: 208.0091. Found $[M+H^+]^+$: 208.0093.

IR: (cm^{-1}) 2920.58, 780.93, 685.58

N,N-dichloro-2-(2-methoxyphenyl)ethan-1-amine (11)

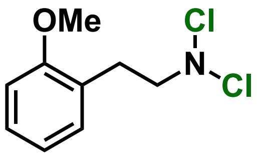

Synthesized from commercially available 2-(2-methoxy-phenyl)-ethylamine (CAS: 2045-79-6) following General Procedure B.

Yield: 93% (yellow oil)

^{1}H NMR (400 MHz, $CDCl_3$) δ 7.25 – 7.12 (m, 2H), 6.95 – 6.82 (m, 2H), 3.84 (t, 5H), 3.08 (t, J = 7.4 Hz, 2H).

^{13}C NMR (126 MHz, $CDCl_3$) δ 157.64, 130.84, 128.34, 125.45, 120.69, 110.44, 75.13, 55.38, 30.15.

HRMS (APCI) m/z: calculated for $C_9H_{12}ONCl_2$ $[M+H^+]^+$: 220.0291. Found $[M+H^+]^+$: 220.0292.

IR: (cm^{-1}) 2936.03, 2834.88, 1241.43, 749.15

2-(2-bromophenyl)-N,N-dichloroethan-1-amine (12)

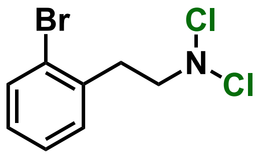

Synthesized from commercially available 2-(2-bromophenyl)ethanamine (CAS: 65165-58-2) following General Procedure B.

Yield: 91% (yellow oil)

^{1}H NMR (500 MHz, $CDCl_3$) δ 7.56 (d, J = 7.6 Hz, 1H), 7.35 – 7.24 (m, 2H), 7.12 (ddd, J = 7.9, 5.8, 3.4 Hz, 1H), 3.96 – 3.82 (m, 2H), 3.35 – 3.16 (m, 2H).

^{13}C NMR (126 MHz, $CDCl_3$) δ 136.64, 133.20, 131.37, 129.43, 128.82, 127.87, 124.62, 74.54, 35.22.

HRMS (APCI) m/z: calculated for $C_8H_9NBrCl_2$ $[M+H^+]^+$: 267.9290. Found $C_8H_8NBrCl_2$ $[M+H^+]^+$: 267.92877.

IR: (cm^{-1}) 2918.53, 1471.02, 1023.67, 747.46

***N,N*-dichloro-2-(2-chlorophenyl)ethan-1-amine (13)**

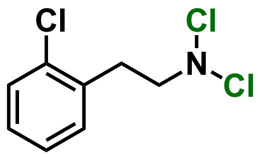

Synthesized from commercially available 2'-chlorophenethylamine (CAS: 13078-80-3) following General Procedure B.

Yield: 96% (yellow oil)

^{1}H NMR (500 MHz, $CDCl_3$) δ 7.41 – 7.33 (m, 1H), 7.33 – 7.12 (m, 3H), 3.96 – 3.76 (m, 2H), 3.26 – 3.14 (m, 2H).

^{13}C NMR (126 MHz, $CDCl_3$) δ 134.90, 134.23, 131.35, 129.86, 128.58, 127.21, 74.46, 32.80.

HRMS (APCI) m/z: calculated for $C_8H_8NCl_3$ $[M]^+$: 222.9717. Found $[M]^+$: 222.9718.

IR: (cm^{-1}) 2919.27, 1474.87, 1053.42, 748.35

***N,N*-dichloro-4-phenylbutan-1-amine (14)**

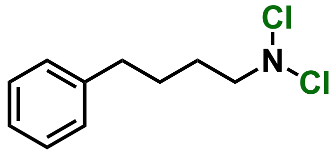

Synthesized from commercially available 4-phenylbutylamine (CAS: 13214-66-9) following General Procedure B.

Yield: 95% (yellow oil)

^{1}H NMR (500 MHz, $CDCl_3$) δ 7.30 (dd, J = 8.7, 6.6 Hz, 2H), 7.19 (d, J = 6.7 Hz, 3H), 3.65 (t, J = 6.8 Hz, 2H), 2.65 (t, J = 7.5 Hz, 2H), 1.97 – 1.58 (m, 4H).

^{13}C NMR (126 MHz, $CDCl_3$) δ 141.81, 128.53, 126.09, 77.36, 75.85, 35.61, 28.28, 27.98.

HRMS (APCI) m/z: calculated for $C_{10}H_{14}NCl_2$ $[M+H^+]^+$: 218.0498. Found $[M+H^+]^+$: 218.04997.

IR: (cm^{-1}) 3025.07, 2938.87, 2857.44, 745.79, 696.74

N-chloro-N-methyl-2-phenylethan-1-amine (17)

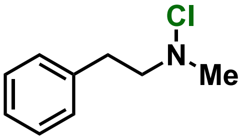

Synthesized from commercially available N-methylphenethylamine (CAS: 589-08-2) following General Procedure B.

Yield: 82% (yellow oil)

^{1}H NMR (500 MHz, $CDCl_3$) δ 7.26 (dd, J = 26.5, 7.4 Hz, 5H), 3.17 – 3.07 (m, 2H), 2.98 (d, J = 6.7 Hz, 5H).

^{13}C NMR (126 MHz, $CDCl_3$) δ 139.15, 128.95, 128.62, 126.47, 67.59, 53.28, 34.82.

HRMS: calculated for $C_9H_{13}NCl$ $[M+H^+]^+$: 170.0731. Found $[M+H^+]^+$: 170.0731.

IR: (cm^{-1}) 3363.13, 3026.84, 2954.47, 747.52, 697.29

N-benzyl-N-chloro-1-phenylmethanamine (18)

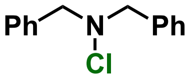

Synthesized from commercially available dibenzylamine (CAS: 103-49-1) following General Procedure B.

Yield: 97% (yellow oil)

^{1}H NMR (400 MHz, $CDCl_3$) δ 7.48 – 7.30 (m, 10H), 4.18 (s, 4H).

^{13}C NMR (101 MHz, $CDCl_3$) δ 137.20, 129.22, 128.52, 128.02, 67.28.

These spectra are consistent with previously reported values.[4]

Product Characterization for *N*-Chlorimines

(*E*)-*N*-chloro-1-phenylmethanimine (20)

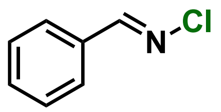

Synthesized from commercially available benzylamine (CAS: 100-46-9) following General Procedure D.

Yield: 94% (clear liquid)

Purification: Eluted in 10% EtOAc in Hexanes (R_f = 0.64)

^{1}H NMR (400 MHz, $CDCl_3$) δ 8.81 (s, 1H), 7.68 (d, *J* = 6.8 Hz, 2H), 7.60 – 7.36 (m, 3H).

^{13}C NMR (101 MHz, $CDCl_3$) δ 172.82, 133.30, 132.25, 129.12, 128.18.

These spectra are consistent with previously reported values.[6]

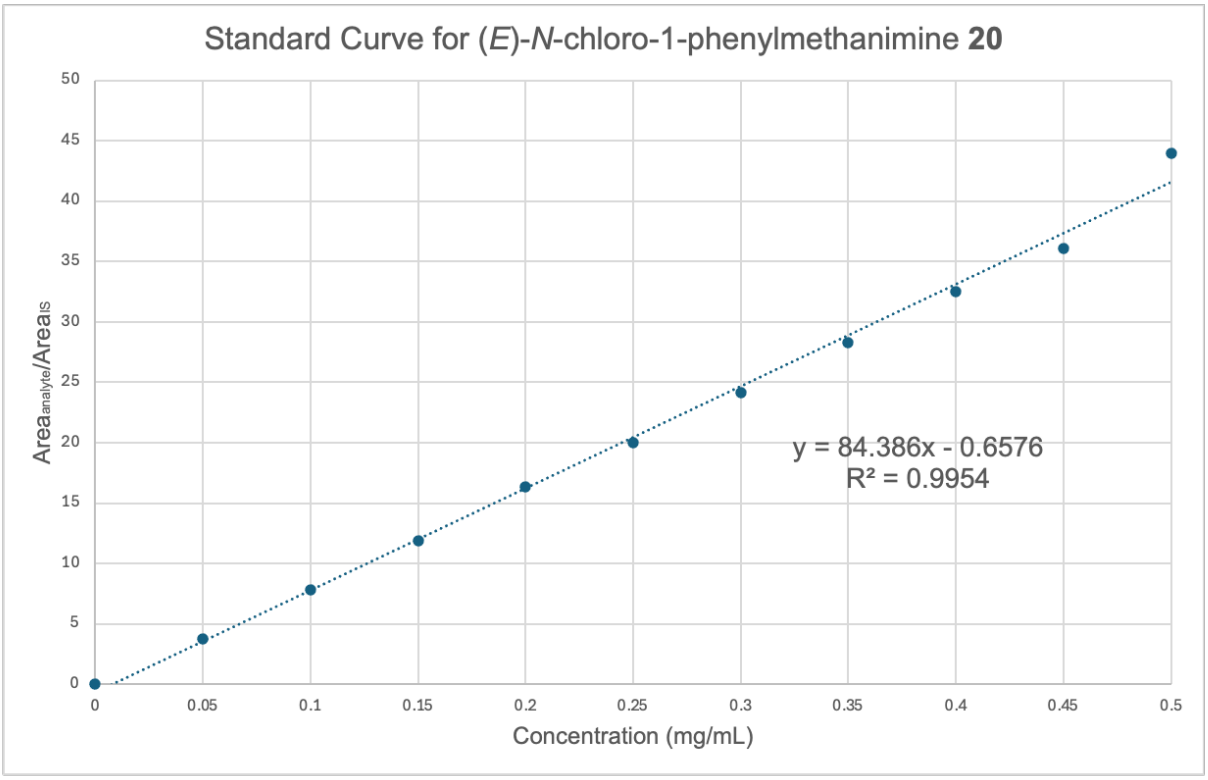

(*E*)-*N*-chloro-1-(4-chlorophenyl)methanimine (21)

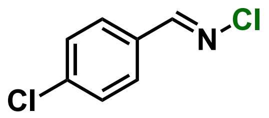

Synthesized from commercially available 4-chlorobenzylamine (CAS:104-86-9) following General Procedure D.

Yield: 83% (white solid)

Purification: Eluted in 10% EtOAc in Hexanes ($R_f = 0.64$)

^{1}H NMR (400 MHz, $CDCl_3$) δ 8.77 (s, 1H), 7.67 – 7.57 (m, 2H), 7.49 – 7.36 (m, 2H).

^{13}C NMR (101 MHz, $CDCl_3$) δ 171.54, 138.48, 131.70, 129.50, 129.34.

HRMS (APCI) m/z: calculated for C_7H_5NCl $[M+H^+]^+$: 173.9871. Found $[M+H^+]^+$: 173.9876.

IR: (cm^{-1}) 1905.88, 1648.38, 860.64, 819.02

(*E*)-1-(4-bromophenyl)-*N*-chloromethanimine (22)

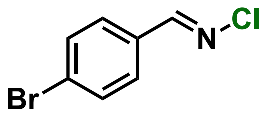

Synthesized from commercially available 4-bromobenzylamine (CAS: 3959-07-7) following General Procedure D.

Yield: 85% (white solid)

Purification: Eluted in 10% EtOAc in Hexanes ($R_f = 0.64$)

^{1}H NMR (400 MHz, $CDCl_3$) δ 8.76 (s, 1H), 7.67 – 7.48 (m, 4H).

^{13}C NMR (101 MHz, $CDCl_3$) δ 171.67, 132.46, 132.11, 129.48, 126.99.

These spectra are consistent with previously reported values. [6]

(*E*)-*N*-chloro-1-(4-fluorophenyl)methanimine (23)

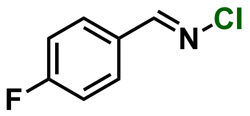

Synthesized from commercially available 4-fluorobenzylamine (CAS: 140-75-0) following General Procedure D.

Yield: 66% (clear crystalline solid)

Purification: Eluted in 10% EtOAc in Hexanes (R_f = 0.6)

^{1}H NMR (400 MHz, $CDCl_3$) δ 8.77 (s, 1H), 7.72 – 7.64 (m, 2H), 7.17 – 7.09 (m, 2H).

^{13}C NMR (101 MHz, $CDCl_3$) δ 171.39, 165.12 (d, $J_{C\text{-}F}$ = 253.8 Hz), 130.31 (d, $J_{C\text{-}F}$ = 8.8 Hz), 129.62 (d, $J_{C\text{-}F}$ = 2.9 Hz), 116.44 (d, $J_{C\text{-}F}$ = 22.4 Hz).

These spectra are consistent with previously reported values. [6]

(*E*)-*N*-chloro-1-(naphthalen-2-yl)methanimine (31)

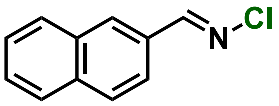

Synthesized from commercially available (1-(2-Naphthyl)methanamine (CAS: 2018-90-8) following General Procedure D.

Yield: 74% (clear crystalline solid)

Purification: Eluted in 5% EtOAc in Hexanes (R_f = 0.38)

^{1}H NMR (400 MHz, $CDCl_3$) δ 8.95 (s, 1H), 7.99 (s, 1H), 7.93 – 7.84 (m, 4H), 7.63 – 7.52 (m, 2H).

^{13}C NMR (101 MHz, $CDCl_3$) δ 172.86, 135.20, 132.93, 130.99, 129.19, 129.00, 128.27, 128.12, 127.14, 122.73.

HRMS (APCI) m/z: calculated for $C_{11}H_9NCl$ $[M+H^+]^+$: 190.0418. Found $[M+H^+]^+$: 190.0420.

IR: (cm^{-1}) 2921.57, 1626.93, 1560.57, 766.46

<u>**Synthetic Utility Protocols**</u>

Gram-Scale Synthesis of *N,N*-dichloro-2-phenylethan-1-amine (2):

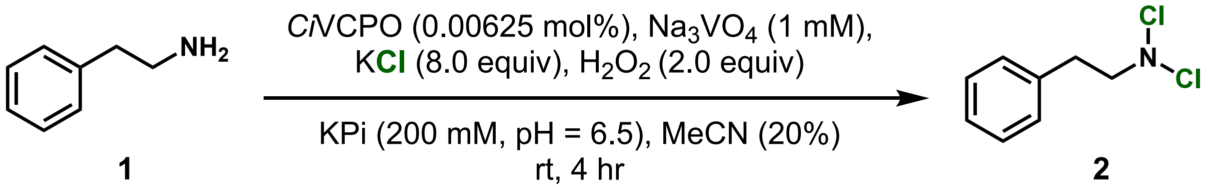

Aliquots of purified VHPO enzyme from *Curvularia inaequalis* (*Ci*VCPO, 10 μM) with a combined volume of 51.5 mL were removed from a -80 °C freezer and allowed to warm to room temperature over 10 min. The enzyme (51.5 mL) was poured into a 150 mL beaker, to which 250 mM Na_3VO_4 (4.12 mL, 0.125 equiv) was added. The combined enzyme and vanadate mixture was allowed to sit at room temperate for 20 minutes. To a 2L Erlenmeyer flask, Milli-Q H_2O (186 mL), 1 M pH 6.5 KPi buffer (206 mL), 176 mM KCl (375 mL, 8.0 equiv), and MeCN (186 mL) were added. Then 2-phenethylamine **1** (1.0 g, 8.25 mmol, 1 equiv) dissolved in MeCN (20 mL, totaling 206 mL MeCN) was added to the reaction mixture. The beaker containing the *Ci*VCPO and Na_3VO_4 was added to the reaction mixture. A 30% stock of H_2O_2 (1.854 mL, 2.2 equiv) was then added to the reaction mixture and the reaction was allowed to shake at room temperature for 4 hours using an Infors HT Multitron Incubator Shaker at 220 rpm. After this time, the reaction mixture was divided equally into four 1L Erlenmeyer flasks and 125 mL of EtOAc was added to each. The quenched mixtures were individually transferred to a 500 mL separatory funnel. Each aqueous layer was extracted with EtOAc (2 x 300 mL) and the combined organic layers were then washed with H_2O (2 x 2.5 mL), brine (2 x 12.5 mL) and dried over Na_2SO_4 for 10 minutes. The dried organic layer was then decanted into a round bottom flask. To ensure adequate transfer, the Na_2SO_4 was rinsed with ethyl acetate and decanted into the round bottom flask three times. The product was then concentrated *in vacuo* without need for further purification to give *N,N*-dichloro-2-phenylethan-1-amine **2** (1.54 g) in 98% yield (yellow oil).

Preparative Procedure for the Synthesis of 2-(dichloroamino)ethan-1-ol *(*15):

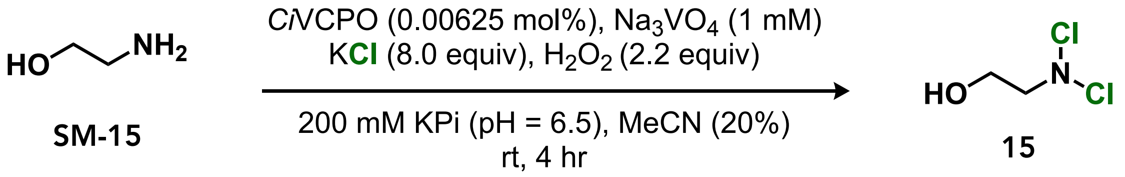

2-(dichloroamino)ethan-1-ol **15** was prepared from 2-aminoethanol **SM-15** (0.8 mmol, 48.9 mg) utilizing *General Procedure B* in 74% yield with a modified workup procedure. The modified workup procedure is as follows. After 4 hours, 1 M HCl is added to the reaction mixture until a pH of 3 is reached (~10 mL). Once the proper pH is met, ethyl acetate (50 mL) is added to the solution and the solution is transferred to a 250 mL separatory funnel. The aqueous layer is extracted with ethyl acetate (2 x 125 mL) and the combined organic layers are washed with H_2O (2 x 10 mL), brine (2 x 5 mL) and dried over sodium sulfate (Na_2SO_4) for 10 minutes. The dried organic layer was then decanted into a round bottom flask. To ensure adequate transfer, the Na_2SO_4 was rinsed with ethyl acetate and decanted into the round bottom flask three times. The product was then concentrated down without need for further purification to give 2-(dichloroamino)ethan-1-ol **15** (77 mg) as a yellow oil in 74% yield.

Compound 15

Yield: 74% (yellow oil)

^{1}H NMR (400 MHz, $CDCl_3$) δ 3.92 – 3.87 (m, 2H), 3.85 – 3.80 (m, 2H).

^{13}C NMR (101 MHz, $CDCl_3$) δ 60.41, 48.96.

HRMS (APCI) m/z: calculated for $C_2H_6ONCl_2$ $[M+H^+]^+$: 129.9821. Found $[M+H^+]^+$: 129.98236

IR: (cm^{-1}) 3330.70, 2926.22, 1063.87

Preparative Procedure for the Synthesis of 3-cyanopropanoic acid (16):

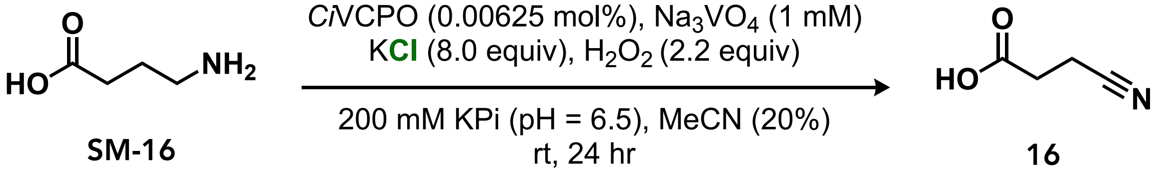

3-cyanopropanoic acid **16** was prepared from 4-aminobutanoic acid **SM-16** (0.8 mmol, 104.1 mg) utilizing a modified version of *General Procedure B* in 80% yield. The modified procedure is as follows. A Falcon® tube containing purified VHPO from *Curvularia inaequalis* (*Ci*VCPO, 10 µM, 5 mL) is removed from a -80 °C freezer and allowed to warm to room temperature over 10 min. An aqueous solution of 250 mM Na_3VO_4 (400 µL) is added to the thawed enzyme, and the enzyme-vanadate solution is allowed to sit at room temperature for a 20-minute incubation period. A 250 mL round bottom flask is charged with 0.8 mmol of the starting substrate. After the addition of a teflon stir bar, MeCN (20 mL) is added. Next, Milli-Q H_2O (18 mL), 1 M pH 6.5 KPi buffer (20 mL), and 176 mM KCl (36.4 mL, 8.0 equiv) are added to the MeCN solution. After the 20-minute incubation of the enzyme-vanadate solution at room temperature, the Falcon® tube containing *Ci*VCPO (0.0125 mol%, 0.5 µM) and Na_3VO_4 (0.125 equiv) is added to the reaction mixture. A 30% stock of H_2O_2 (180 µL, 2.2 equiv) is added to the reaction mixture and the resulting solution is allowed to stir at room temperature for 24 hours unless otherwise noted. After this time, 1 M HCl is added to the reaction mixture until a pH of 3 is reached (~10 mL). Once the proper pH is met, ethyl acetate (50 mL) is added to the solution and the solution is transferred to a 250 mL separatory funnel. The aqueous layer is extracted with ethyl acetate (2 x 125 mL) and the combined organic layers are washed with H_2O (2 x 10 mL), brine (2 x 5 mL) and dried over sodium sulfate (Na_2SO_4) for 10 minutes. The dried organic layer was then decanted into a round bottom flask. To ensure adequate transfer, the Na_2SO_4 was rinsed with ethyl acetate and decanted into the round bottom flask three times. The product was then concentrated down without need for further purification to give 3-cyanopropanoic acid **16** (63 mg) as a clear oil in 80% yield.

Compound 16

Yield: 80% (clear oil)

^{1}H NMR (400 MHz, $CDCl_3$) δ 8.73 (s, 1H), 2.78 (t, *J* = 7.1 Hz, 2H), 2.66 (t, *J* = 7.1 Hz, 2H).

^{13}C NMR (101 MHz, $CDCl_3$) δ 74.95, 118.34, 29.84, 12.90

These spectra are consistent with previously reported values.[7]

Preparative Procedure for the Synthesis of 2-(4-(1-chloropiperidin-3-yl)phenyl)-2H-indazole-7-carboxamide (19):

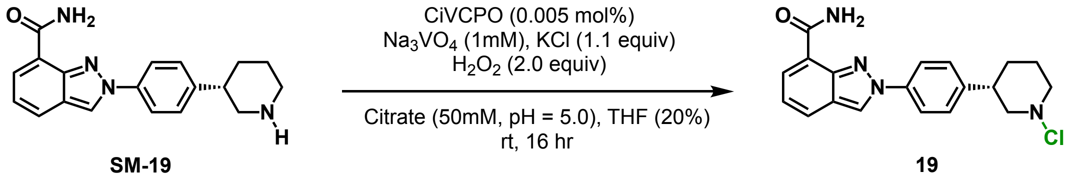

An Eppendorf tube containing purified VHPO from *Curvularia inaequalis* (*Ci*VCPO, 10 µM, 0.50 ml) was removed from a -80 °C freezer and allowed to warm to room temperature over 10 min. An aqueous solution of 250 mM Na_3VO_4 (40 µL) was added to the thawed enzyme, and the enzyme- Na_3VO_4 solution was allowed to sit at room temperature for a 20-minute incubation period. To a 100 mL round bottom flask containing a teflon stir bar, H_2O (5.3 ml), 500mM pH 5 citrate buffer (1.0mL), THF (2.0 ml), and 100mM KCl (1.1 ml, 1.1 eq) was added. Niraparib (**SM-19**, 32.0 mg, 0.100 mmol) was added directly into the reaction mixture, followed by the *Ci*VCPO solution (500 µL, 10 µmolar, 0.005 mol%). A 10% stock solution of H_2O_2 (68 µL, 2.0 equiv) was then added to the reaction mixture and the resulting solution was stirred vigorously for 16 hours at room temperature while covered with aluminum foil. As the reaction proceeded, the solution turned a transparent yellow and a white solid precipitates from the reaction mixture. After 16 hours the reaction was filtered over a Pyrex fritted funnel, leaving behind (*S*)-2-(4-(1-chloropiperidin-3-yl)phenyl)-2H-indazole-7-carboxamide (**19**, 33 mg, 93 µmol, 93 %) as a powdery white solid. This was left to dry by vacuum for an additional 10 minutes to remove additional water before characterization due to its highly hygroscopic nature.

A 400 µl sample of the crude reaction mixture was diluted with 600 µl of acetonitrile, briefly sonicated, and left to slowly evaporate overnight to yield fine, needle-like crystals for crystallography.

Compound 19

Yield: 93% (white solid)

1H NMR (400 MHz, DMSO): δ 9.29 (s, 1H), 8.58 (d, J = 2.9 Hz, 1H), 8.12 – 7.99 (m, 4H), 7.89 (d, J = 2.9 Hz, 1H), 7.58 – 7.50 (m, 2H), 7.28 (dd, J = 8.4, 7.0 Hz, 1H), 3.46 (d, J = 9.1 Hz, 2H), 3.15 – 2.80 (m, 3H), 1.93 – 1.35 (m, 4H).

^{13}C NMR (151 MHz, DMSO): δ 165.61, 146.11, 143.34, 138.02, 129.78, 128.59, 125.42, 123.46, 123.44, 121.96, 121.48, 120.84, 68.38, 62.38, 43.27, 29.59, 26.63.

HRMS (APCI) m/z: calculated for $C_9H_{13}NCl$ $[M+H^+]^+$: 355.13068. Found $[M+H^+]^+$: 355.1312.

Preparative Procedure for the Synthesis of *N,N*-dichloro-1-phenylmethanamine (24):

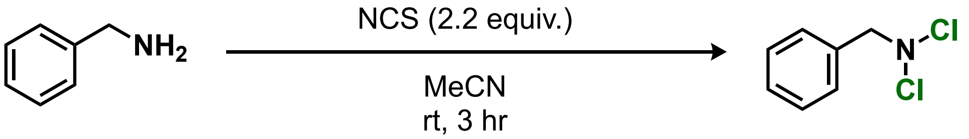

N,N-dichloro-1-phenylmethanamine **24** was prepared from benzylamine **25** (0.93 mmol, 100 mg) in 57% yield. A 10 mL round bottom flask is charged with 0.93 mmol benzylamine and a teflon stir bar is added. After MeCN (4.5 mL) is added to the flask, NCS (2.05 mmol, 274 mg) is added to the solution and allowed to stir at room temperature and monitored by TLC. After completion of the reaction (3 hours), EtOAc (3 mL) was added to the round bottom flask and the solution was transferred to a 60 mL separatory funnel. To the separatory funnel, 5 mL of H_2O added along with an additional 5 mL of EtOAc. The organic layer is collected and the aqueous layer is extracted with ethyl acetate (2 x 5 mL) and the combined organic layers are washed with H_2O (2 x 10 mL), brine (2 x 5 mL) and dried over sodium sulfate (Na_2SO_4) for 10 minutes.The dried organic layer was then decanted into a round bottom flask. To ensure adequate transfer, the Na_2SO_4 was rinsed with ethyl acetate and decanted into the round bottom flask three times. The product was then concentrated down and purified via column chromatography with 5% EtOAc/Hex (R_f = 0.55) to afford *N,N*-dichloro-1-phenylmethanamine **24** (93 mg) as a yellow oil in 57% yield.

Compound 24

Yield: 57% (yellow oil)

^{1}H NMR (400 MHz, $CDCl_3$) δ 7.40 (s, 5H), 4.69 (s, 2H).

^{13}C NMR (101 MHz, $CDCl_3$) δ 135.04, 130.22, 129.40, 128.72, 79.02.

These spectra are consistent with previously reported values.[4]

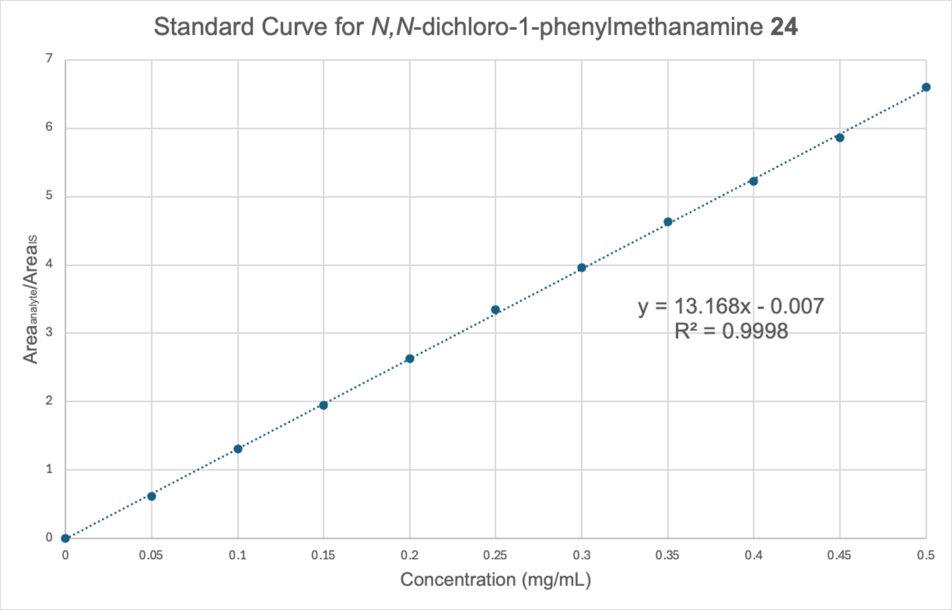

Preparative Procedure for the Synthesis of 2-phenylacetonitrile (26):

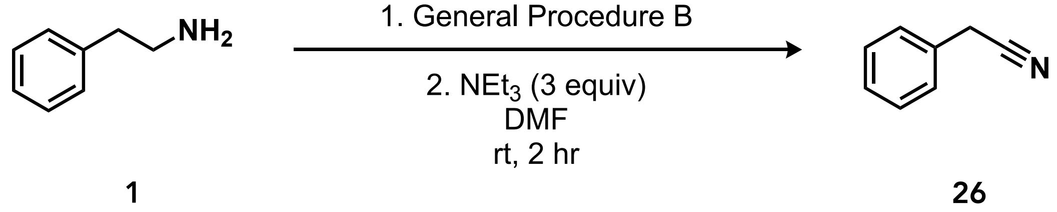

N,N-dichloro-2-phenylethan-1-amine **2** was prepared from **1** (0.8 mmol, 96.9 mg) utilizing *General Procedure B* in 99% yield. To access the corresponding nitrile, the following was adapted according to a literature procedure:[7] To a 10 mL round bottom flask, **2** (0.25 mmol, 47.51 mg) was dissolved in 5 mL DMF. To the mixture, NEt_3 (3 equiv, 75.9 mg) was added to the mixture and allowed to stir at room temperature for 2 hours. The reaction mixture was quenched with H_2O (5 mL) and 1 M HCl (0.2 mL). The reaction mixture was then extracted with Et_2O (3x10 mL), washed with H_2O (5x10 mL), brine (2x5 mL), and dried over Na_2SO_4. The dried organic layer was then decanted into a round bottom flask. To ensure adequate transfer, the Na_2SO_4. was rinsed with Et_2O and decanted into the round bottom flask three times. The product was then concentrated under vacuum to afford 2-phenylacetonitrile **26** (29 mg) as a light yellow liquid in 99% yield over two steps.

Compound 26

^{1}H NMR (400 MHz, $CDCl_3$) δ 7.42 – 7.31 (m, 5H), 3.76 (s, 2H).

^{13}C NMR (101 MHz, $CDCl_3$) δ 130.00, 129.27, 128.18, 128.04, 118.01, 23.75.

These spectra are consistent with previously reported values.[8]

Preparative Procedure for the Synthesis of benzonitrile (27):

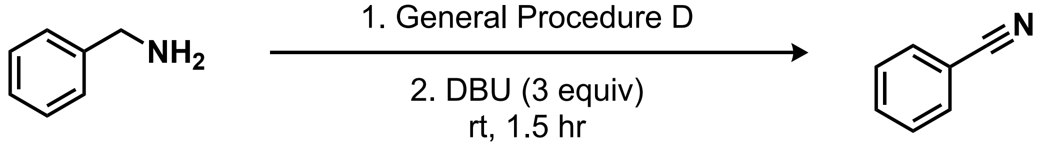

25 27

(E)-N-chloro-1-phenylmethanimine **20** was prepared from **25** (0.4 mmol, 52.5 mg) utilizing *General Procedure D* in 94% yield. To access the corresponding nitrile, **27** (0.256 mmol, 35.7 mg) was added to a 10 mL round bottom flask and dissolved in 5 mL DMF. To the mixture, DBU (3 equiv, 0.767 mmol, 117 mg) was added to the mixture and allowed to stir at room temperature and monitored by TLC. After 1.5 hours, the reaction mixture was quenched with H_2O (5 mL) and 1 M HCl (0.2 mL). The reaction mixture was then extracted with Et_2O (3x10 mL), washed with H_2O (5x10 mL), brine (5x5 mL), and dried over Na_2SO_4. The dried organic layer was then decanted into a round bottom flask. To ensure adequate transfer, the Na_2SO_4 was rinsed with Et_2O and decanted into the round bottom flask three times. The product was purified via column chromatography with 10% EtOAc/Hex (R_f = 0.37) to afford benzonitrile **27** (26 mg) as a clear liquid in 85% yield over two steps.

Compound 27

^1H NMR (400 MHz, $CDCl_3$) δ 7.70 – 7.57 (m, 3H), 7.47 (t, *J* = 7.6 Hz, 2H).

^{13}C NMR (101 MHz, $CDCl_3$) δ 132.89, 132.26, 129.23, 118.97, 112.53.

These spectra are consistent with previously reported values.[9]

Preparative Procedure for the Synthesis of *N,N*-dibenzylbenzamide (26):

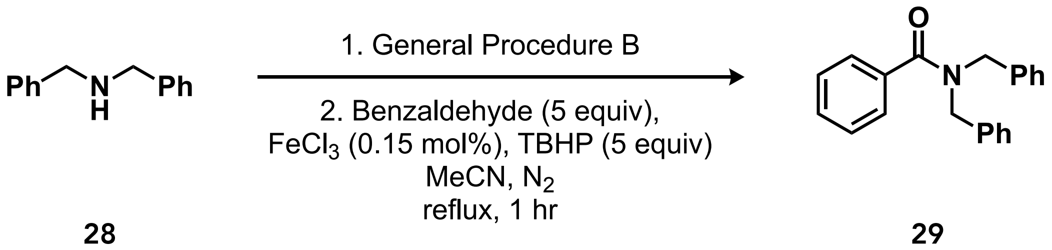

N-benzyl-*N*-chloro-1-phenylmethanamine **18** was prepared from dibenzylamine **28** (0.8 mmol, 157.8 mg) utilizing *General Procedure B* in 97% yield. To access the corresponding amide, the following was adapted according to a literature procedure.[10] To a 50 mL RBF, **18** (0.32 mmol, 74.15 mg) and benzaldehyde (5 equiv, 1.6 mmol, 169.6 mg) were dissolved in MeCN (8 mL) under N_2. To the mixture, 70% TBHP in H_2O (5 equiv, 1.6 mmol, 0.198 mL) was added slowly. Next, $FeCl_3$ (0.15 mol%, 0.048 mmol, 7.78 mg) was dissolved in 2 mL MeCN and added dropwise to the reaction mixture. Once all components were added, the reaction mixture was heated to reflux (85°C) and stirred for 1 hour. The reaction mixture was quenched with 20 mL sat. Na_2SO_3, extracted with 40 mL Et_2O, and dried over Na_2SO_4. The dried organic layer was then decanted into a round bottom flask. To ensure adequate transfer, the Na_2SO_4 was rinsed with Et_2O and decanted into the round bottom flask three times. The product was purified via column chromatography with 25% EtOAc/Hex (R_f = 0.45) to afford *N,N*-dibenzylbenzamide **29** (62 mg) as a white solid in 62% yield over two steps.

Compound 29

^{1}H NMR (400 MHz, $CDCl_3$) δ 7.50 (dt, *J* = 4.6, 2.5 Hz, 2H), 7.41 – 7.27 (m, 11H), 7.14 (d, *J* = 7.2 Hz, 2H), 4.71 (s, 2H), 4.40 (s, 2H).

^{13}C NMR (101 MHz, $CDCl_3$) δ 172.40, 137.06, 136.55, 136.27, 129.79, 128.84, 128.69, 128.54, 127.79, 127.16, 126.84, 51.65, 46.95.

These spectra are consistent with previously reported values.[10]

Preparative Procedure for the Synthesis of 2-naphthaldehyde (29):

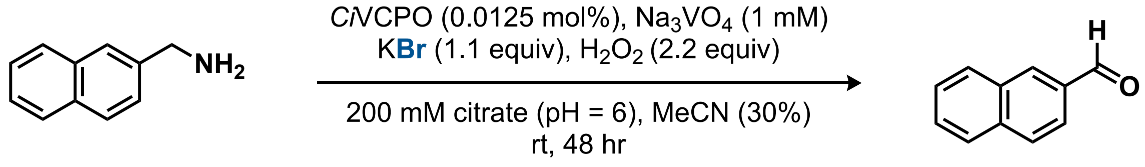

30 32

A Falcon® tube containing purified VHPO from *Curvularia inaequalis* (*Ci*VCPO, 10 µM, 5 mL) was removed from a -80 °C freezer and allowed to warm to room temperature over 10 min. An aqueous solution of 250 mM Na_3VO_4 (400 µL) was added to the thawed enzyme, and the enzyme-vanadate solution was allowed to sit at room temperature for a 20-minute incubation period. A 250 mL round bottom flask was charged with 0.4 mmol of **30**. After addition of a teflon stir bar, MeCN (30 mL) was added. Next, Milli-Q H_2O (44 mL), 1 M pH 6 citrate buffer (20 mL), and 1 M KBr (0.44 mL, 2.2 equiv) were added to the MeCN solution. After the 20-minute incubation of the enzyme-vanadate solution at room temperature, the Falcon® tube containing the *Ci*VCPO (0.0125 mol%, 0.5 µM) and Na_3VO_4 (0.25 equiv) was added to the reaction mixture. A 30% stock of H_2O_2 (90 µL, 2.2 equiv) was then added to the reaction mixture and the reaction was allowed to stir at room temperature for 48 hours. After this time, the reaction mixture was quenched with ethyl acetate (50 mL) and transferred to a 250 mL separatory funnel. The aqueous layer was extracted with EtOAc (3 x 125 mL) and the combined organic layers were then washed with H_2O (2 x 20 mL), brine (1 x 10 mL) and dried over Na_2SO_4 for 10 minutes. To ensure adequate transfer, the Na_2SO_4 was rinsed with ethyl acetate and decanted into the round bottom flask three times. The product was then concentrated *in vacuo* and purified via column chromatography with 10% EtOAc/90% Hexane (R_f = 0.35) to afford 2-naphthaldehyde **32** (47 mg) as a white solid in 75% yield.

Compound 32

^{1}H NMR (400 MHz, $CDCl_3$) δ 10.16 (s, 1H), 8.34 (s, 1H), 8.06 – 7.83 (m, 4H), 7.77 – 7.52 (m, 2H).

^{13}C NMR (101 MHz, $CDCl_3$) δ 192.43, 136.57, 134.73, 134.21, 132.74, 129.65, 129.25, 129.23, 128.21, 127.22, 122.86.

These spectra are consistent with previously reported values.[11]

Optimization Data

All optimization reactions were performed using the General Analytical Procedure for *N*-chlorination with purified *Ci*VCPO (**General Procedure A**), displayed below:

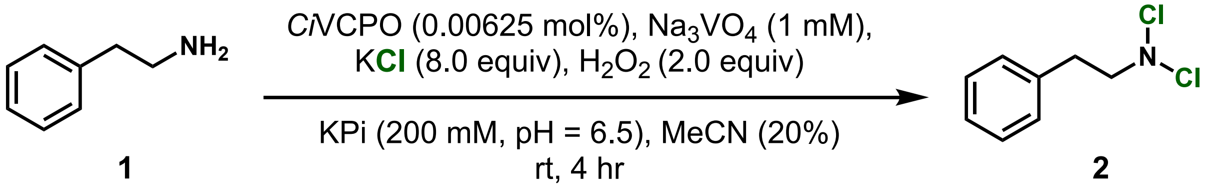

The following figures represent single-variable changes in the standard analytical conditions (**General Procedure A**), with the rest of the variables held constant. Reactions were performed in triplicate, with standard-deviation error bars indicated accordingly. *(Note: 100 μL of a 8 mg/mL solution of 1,3,5-trimethoxybenzene was added as an internal standard for yield confirmation, where applicable)*

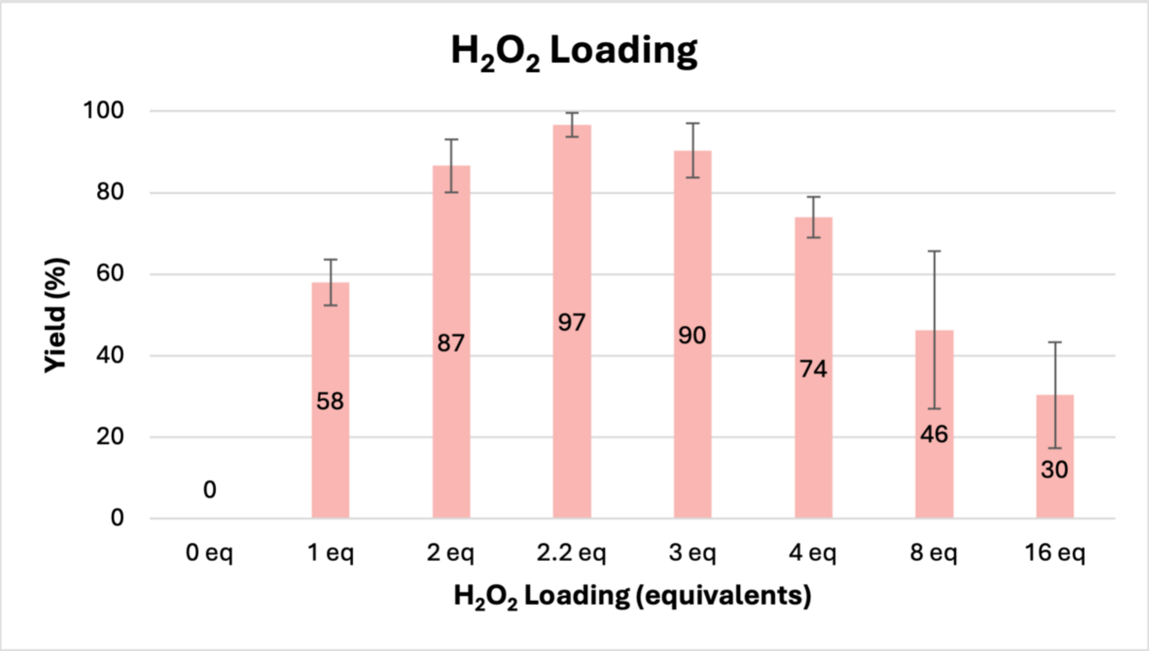

Figure S4. Hydrogen Peroxide (H_2O_2) Loading Screen for Production of 2

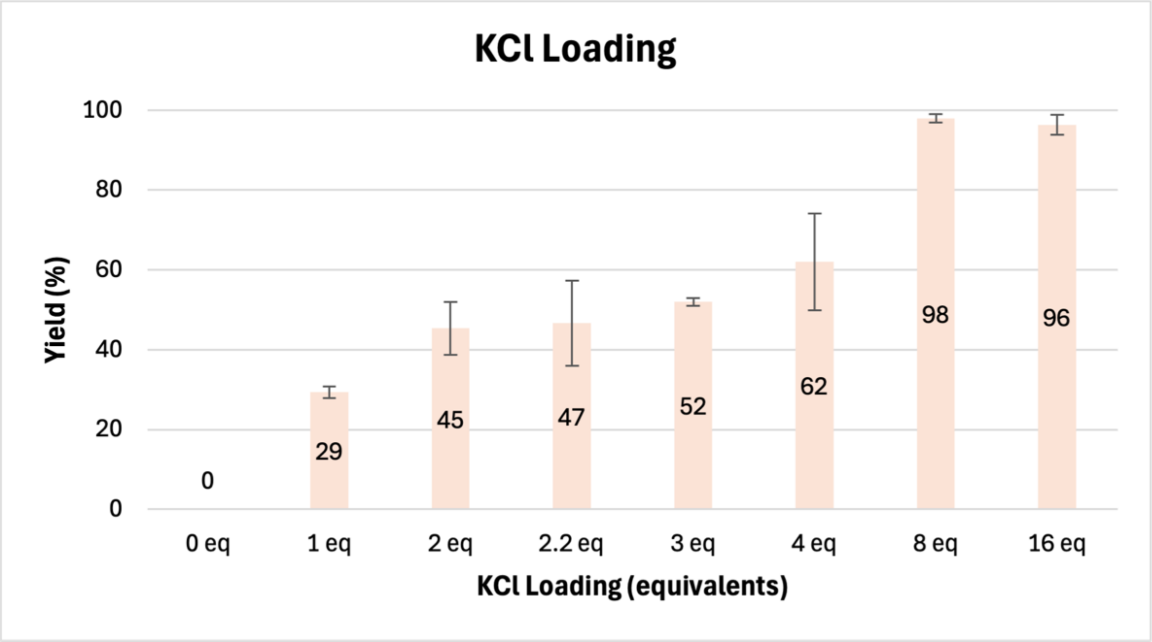

Figure S5. Potassium Chloride (KCl) Loading Screen for Production of 2

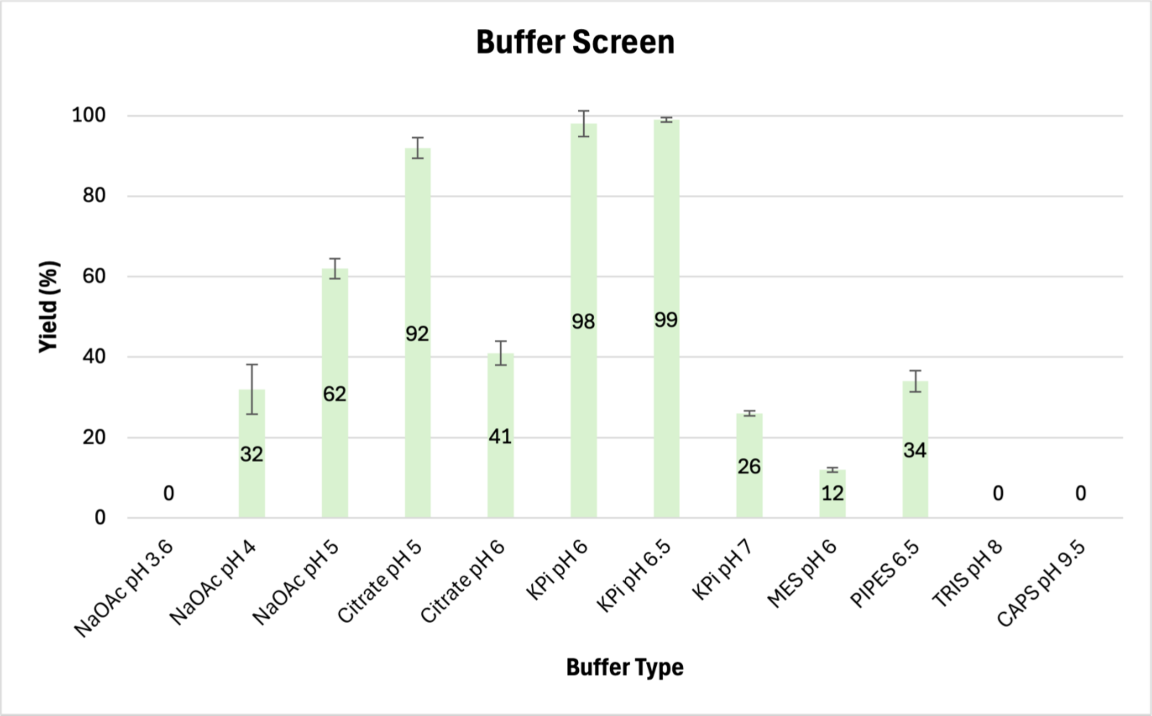

Figure S6. Buffer Type and pH Screen for Production of 2

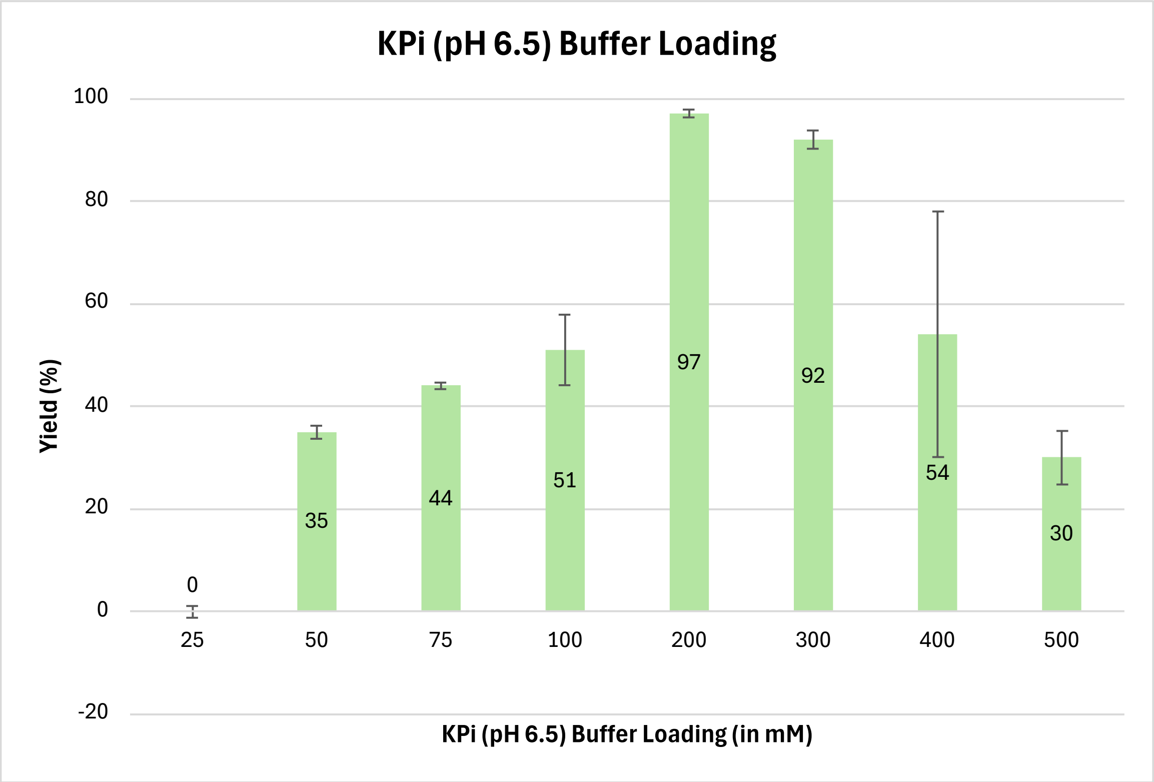

Figure S7. KPi (pH 6.5) Buffer Loading Screen for Production of 2

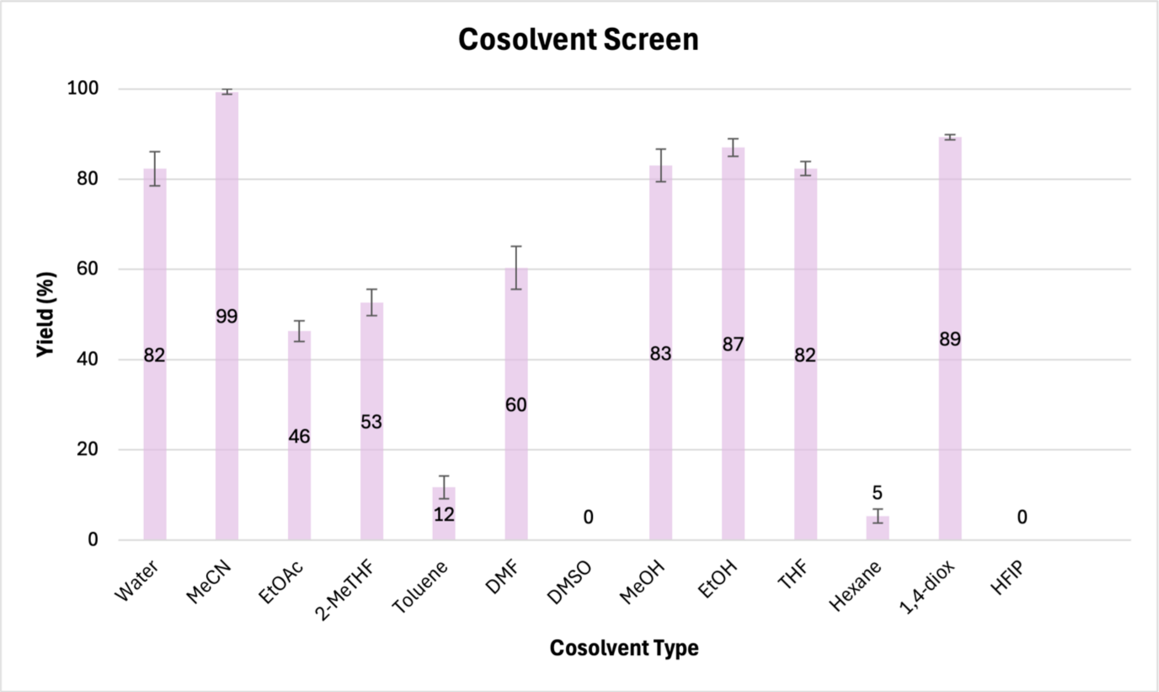

Figure S8. Cosolvent Screen for Production of 2

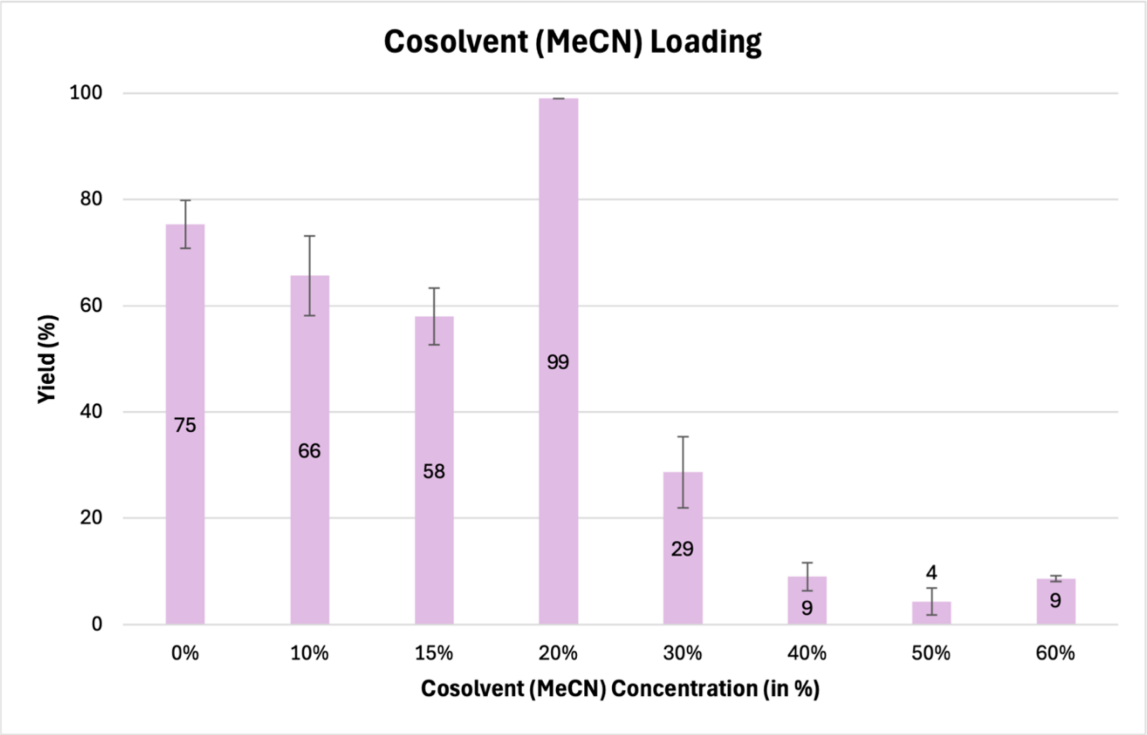

Figure S9. Cosolvent (MeCN) Loading Screen for Production of 2

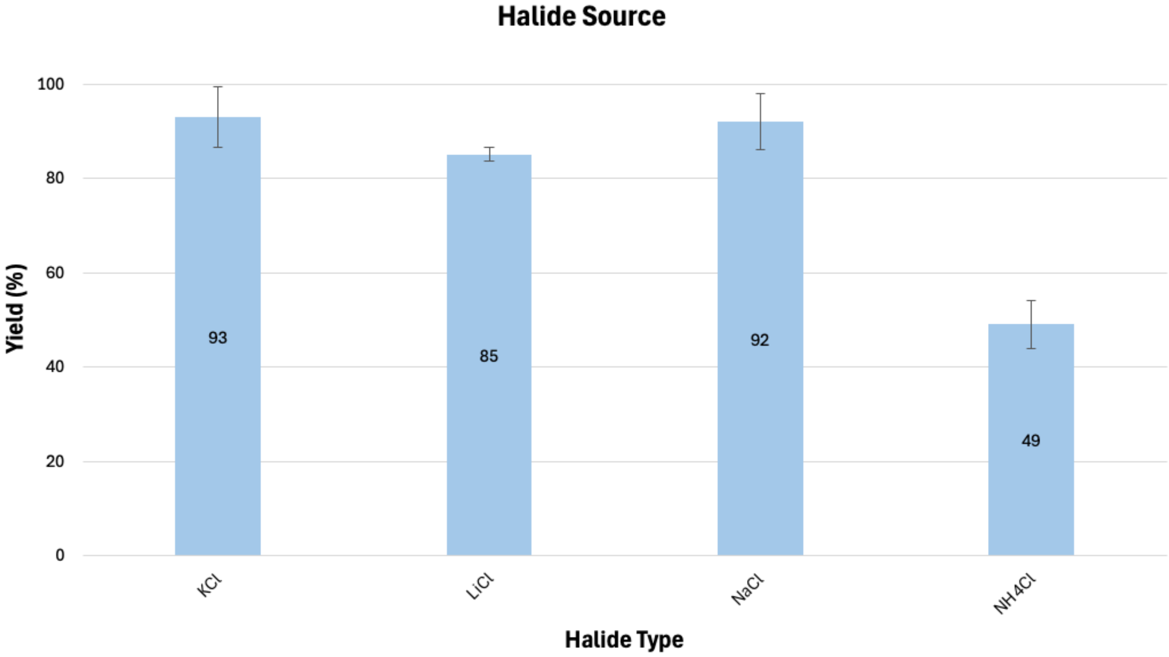

Figure S10. Halide Source Screen for Production of 2

$$\text{atom economy} = \frac{\text{molar mass product}}{\text{molar mass reactants}}$$

This work, *Ci*VCPO:

Reactants	Molecular Weight (g/mol)	Equivalents
**1**	121.2	1
**KCl**	74.55	2
**H_2O_2**	34.01	1

Atom Economy:

$$\frac{190.12}{121.2 + 2 \times 74.55 + 34.01} = 0.62$$

Balanced Equation Used:

$$\underset{\mathbf{1}}{C_8H_{11}N} + 2\ KCl + H_2O_2 \longrightarrow \underset{\mathbf{2}}{C_8H_9NCl_2} + H_2O + 2\ KOH$$

TCCA:

Reactants	Molecular Weight (g/mol)	Equivalents
**1**	121.2	3
**TCCA**	232.41	2

Atom Economy:

$$\frac{3 \times 190.12}{3 \times 121.2 + 2 \times 232.41} = 0.69$$

Balanced Equation Used:

$$3\ \underset{\mathbf{1}}{C_8H_{11}N} + 2\ C_3Cl_3N_3O_3 \longrightarrow 3\ \underset{\mathbf{2}}{C_8H_9NCl_2} + 2\ C_3H_3N_3O_3$$

Oxone-NaCl:

Reactants	Molecular Weight (g/mol)	Equivalents
**1**	121.2	1
**oxone***	614.74	1
**NaCl**	58.44	2

*sum of corresponding molecular weights of 2 $KHSO_5$ + $KHSO_4$ + K_2SO_4

Atom Economy:

$$\frac{190.12}{121.2 + 614.74 + 2 \times 58.44} = 0.22$$

Balanced Equation Used:

$$\underset{\mathbf{1}}{C_8H_{11}N} + 2\ NaCl + 2\ KHSO_5 + KHSO_4 + K_2SO_4 \longrightarrow \underset{\mathbf{2}}{C_8H_9NCl_2} + 3\ KHSO_4 + K_2SO_4 + 2\ NaOH + 1/_2\ O_2$$

Molecular Weight Product: 190.12 g/mol

Figure S11. Atom Economy Metrics for Enzymatic vs Chemical N-Chlorination

References

[1] Wells, C. E.; Ramos, L. P. T.; Harstad, L. J.; Hessefort, L. Z.; Lee, H. J.; Sharma, M.; Biegasiewicz, K. F. Decarboxylative Bromooxidation of Indoles by a Vanadium Haloperoxidase. *ACS Catal.* **2023**, *13*, 4622-4628.

[2] Twist Bioscience. Codon Optimization Tool. https://www.twistbioscience.com/resources/digital-tools/codon-optimization-tool (accessed May 21, 2025).

[3] Gasteiger E.; Hoogland C.; Gattiker A.; Duvaud S.; Wilkins M.R.; Appel R.D.; Bairoch A. Protein Identification and Analysis Tools on the Expasy Server; http://www.springer.com/life+sciences/biochemistry+%26+biophysics/book/978-1-58829-343-5. pp. 571-607 (accessed May 21, 2025).

[4] Sriramoju, V.; Kurva, S.; Madabhushi, S. New Method for the Preparation of *N* -Chloroamines by Oxidative *N* -Halogenation of Amines Using Oxone-KCl. *Synthetic Communications* **2018**, *48*, 699–704.

[5] Yamada, A.; Kitamura, H.; Yamaguchi, K.; Fukuzawa, S.; Kamijima, C.; Yazawa, K.; Kuramoto, M.; Wang, G.-Y.-S.; Fujitani, Y.; Uemura, D. Development of Chemical Substances Regulating Biofilm Formation. *Bulletin of the Chemical Society of Japan* **1997**, *70*, 3061–3069.

[6] Jin, C.; Wang, F.; Sun, B.; Zhuang, X. A Practical Oxidative Conversion of Aldehydes into N-Chloroaldimines. *Journal of Chemical Research* **2018**, *42*, 547–551.

[7] Lunic, D.; Sanosa, N.; Funes-Ardoiz, I.; Teskey, C. J. Mild and Chemoselective Carboxylic Acid Reduction Promoted by Borane Catalysis. *Angew Chem Int Ed* **2022**, *61*, e202207647.

[8] Bag, S.; Dhibar, A.; Moorthy, S.; Ashokan, A.; Sahoo, B. Photocatalytic C–C Bond Azidation and Cyanation of Acyclic Ketones via a Pro-Aromatic Intermediate. *Org. Lett.* **2025**, *27*, 783–788.

[9] Kobayashi, K.; Masuda, K.; Feng, F.; Rashed, Md. N.; Sato, K.; Koumura, N.; Kobayashi, S. A Continuous-Flow Method for the Transformation from Amides to Nitriles Catalyzed by CeO_2 in Acetonitrile. *Adv Synth Catal* **2023**, *365*, 1618–1622.

[10] Porcheddu, A.; Luca, L. D. Iron-Catalyzed Amidation of Aldehydes with *N* -Chloroamines. *Adv Synth Catal* **2012**, *354*, 2949–2953.

[11] Wang, A.; Jiang, H. Palladium-Catalyzed Direct Oxidation of Alkenes with Molecular Oxygen: General and Practical Methods for the Preparation of 1,2-Diols, Aldehydes, and Ketones. *J. Org. Chem.* **2010**, *75*, 2321–2326.

Spectroscopic Data

N,N-dichloro-2-phenylethan-1-amine (2)

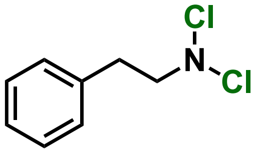

^{1}H-NMR
$CDCl_3$

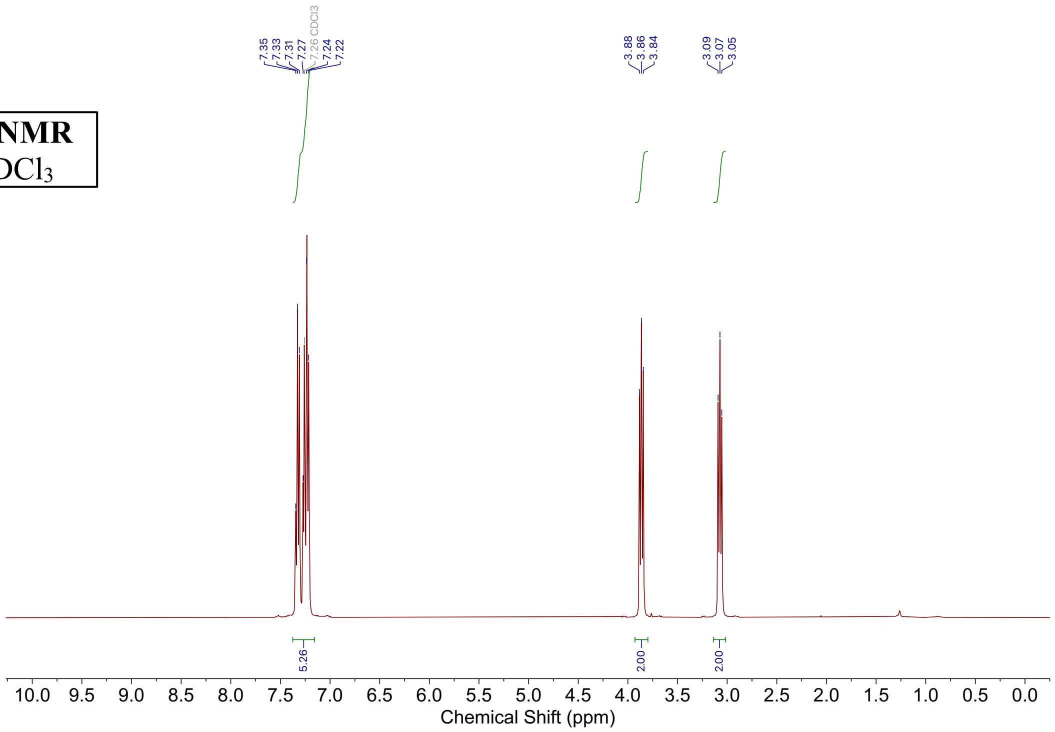

^{13}C-NMR
$CDCl_3$

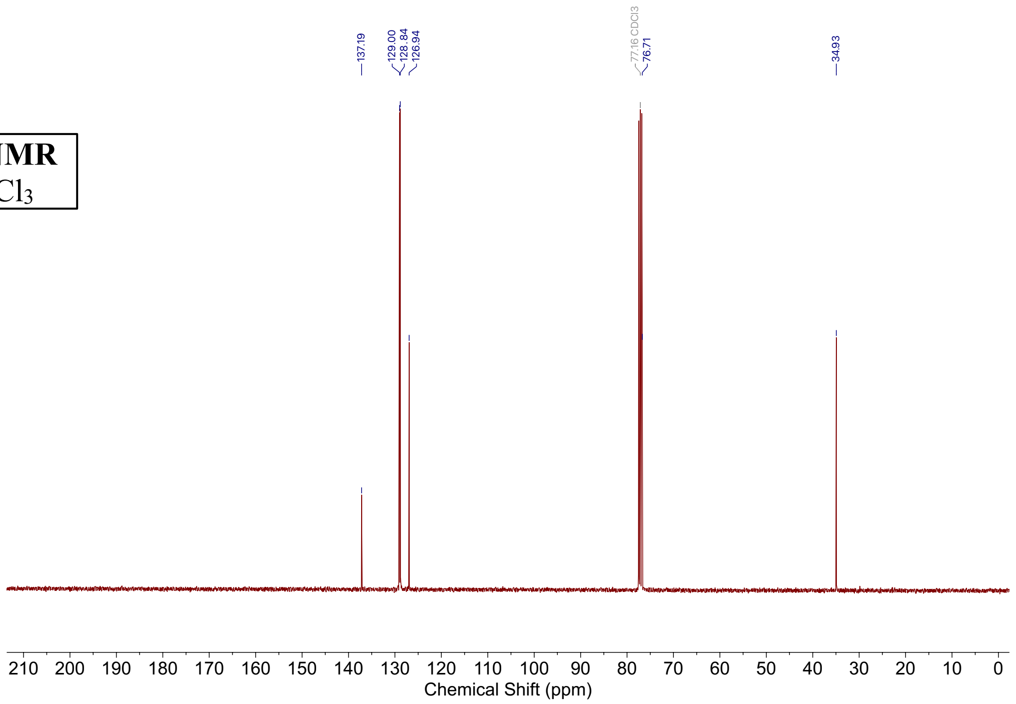

_N,N_-dichloro-2-(p-tolyl)ethan-1-amine (3)

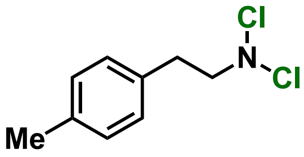

^{1}H-NMR
$CDCl_3$

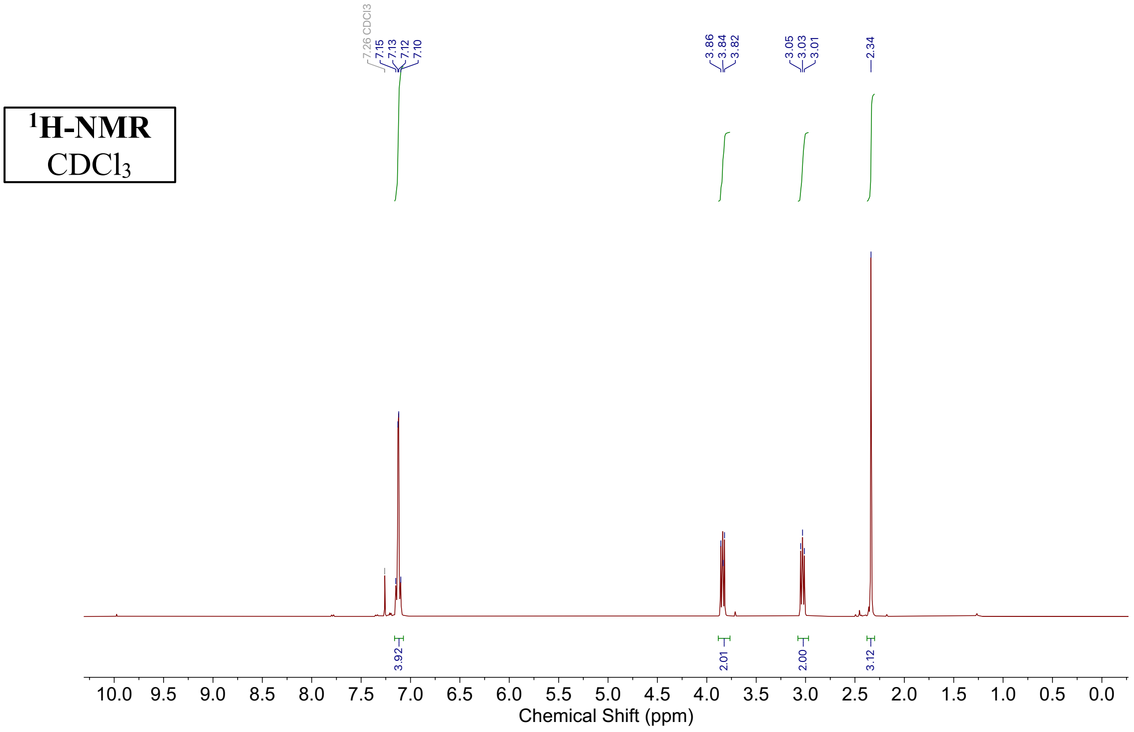

^{13}C-NMR
$CDCl_3$

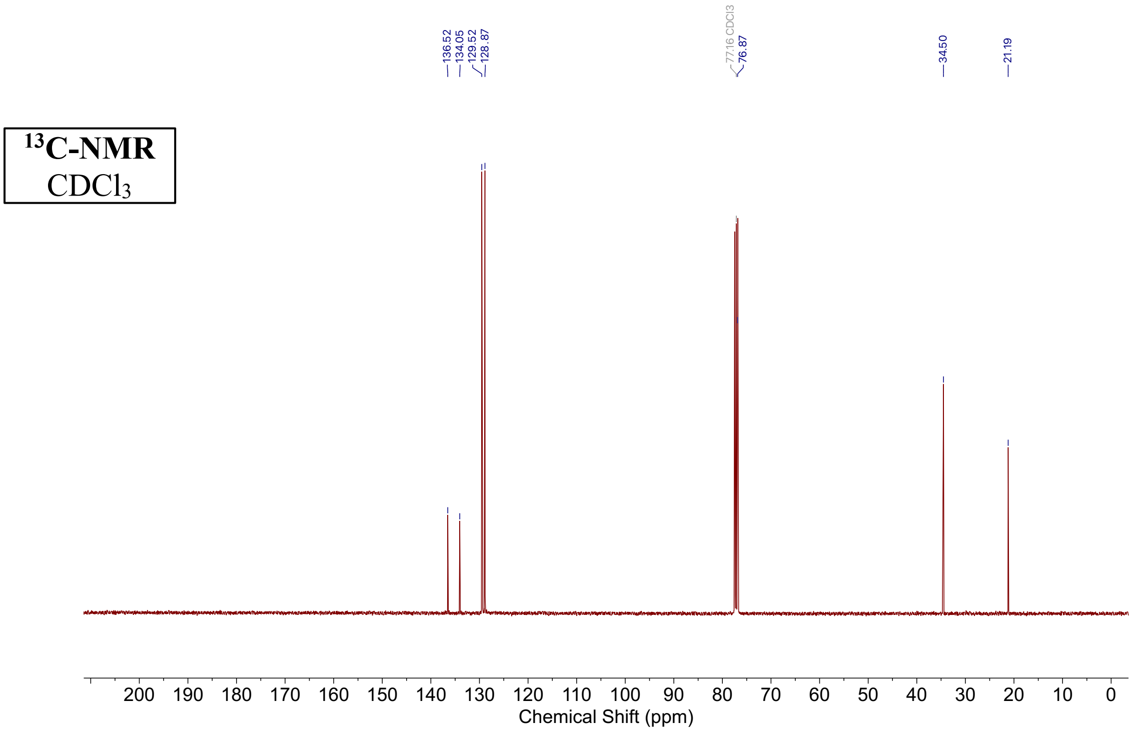

***N,N*-dichloro-2-(4-methoxyphenyl)ethan-1-amine (4)**

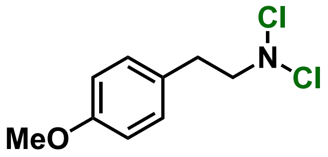

^{1}H-NMR
CDCl3

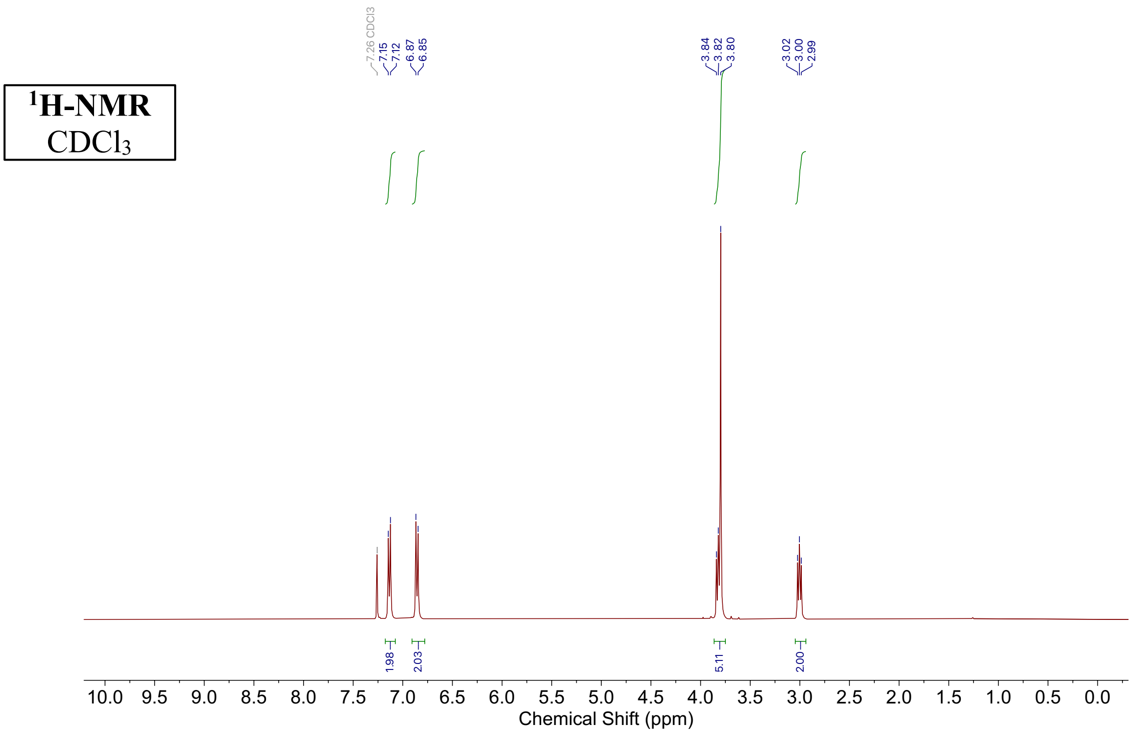

^{13}C-NMR
CDCl3

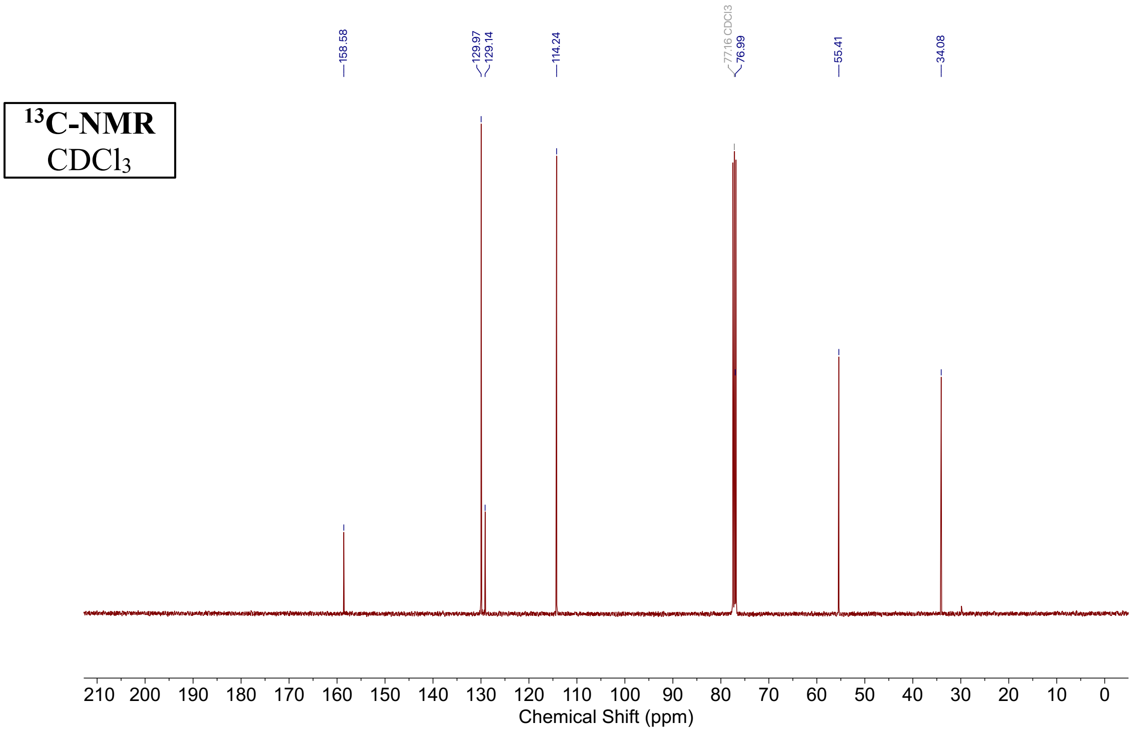

***N,N*-dichloro-2-(4-chlorophenyl)ethan-1- (5)**

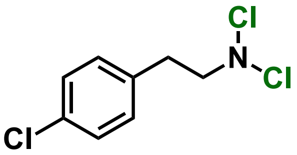

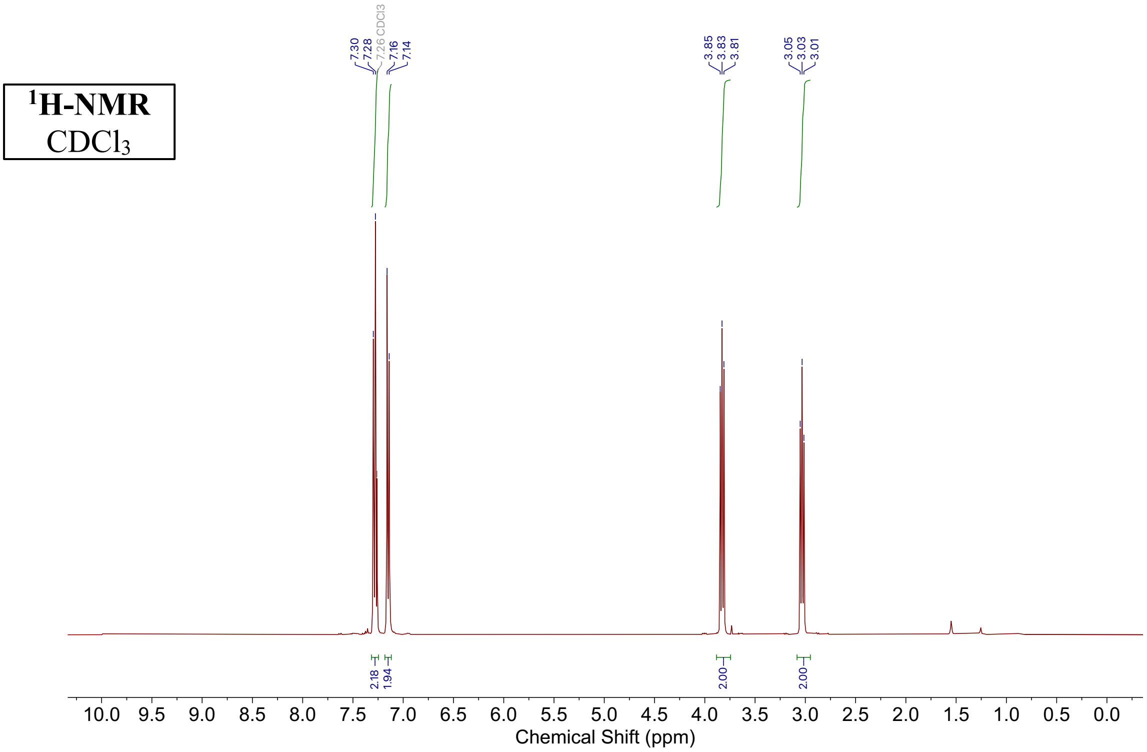

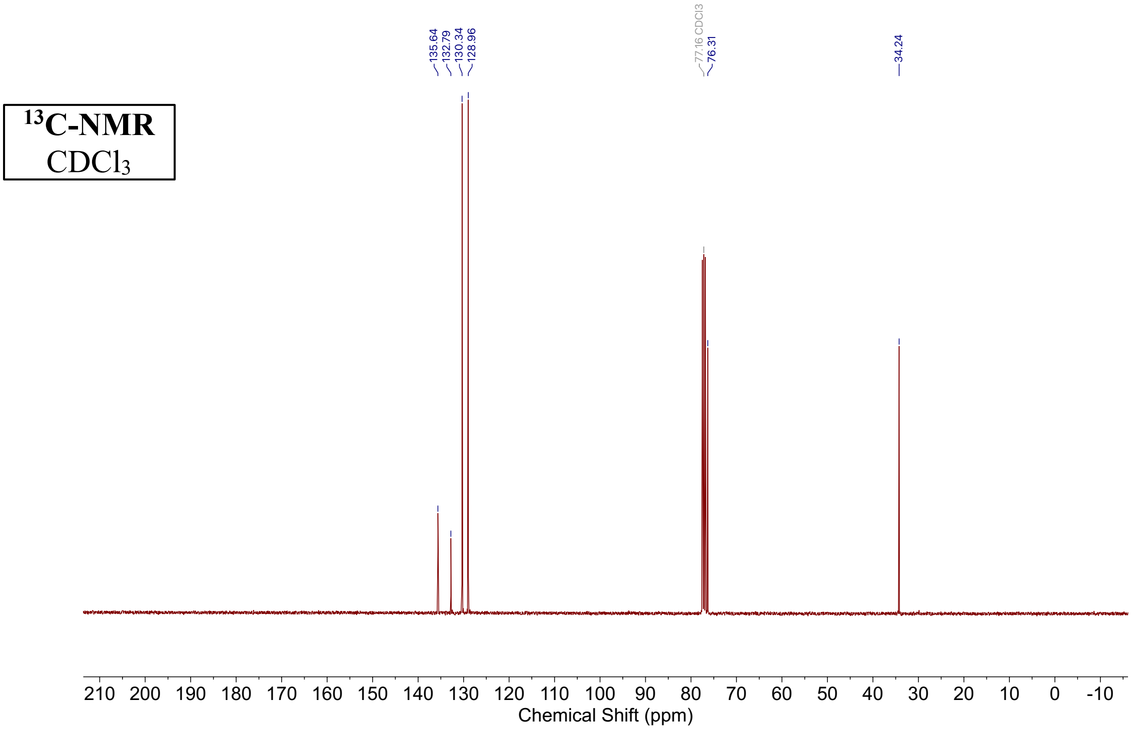

***N,N*-dichloro-2-(4-fluorophenyl)ethan-1-amine (6)**

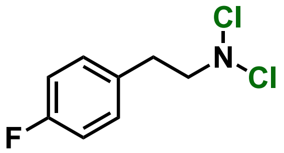

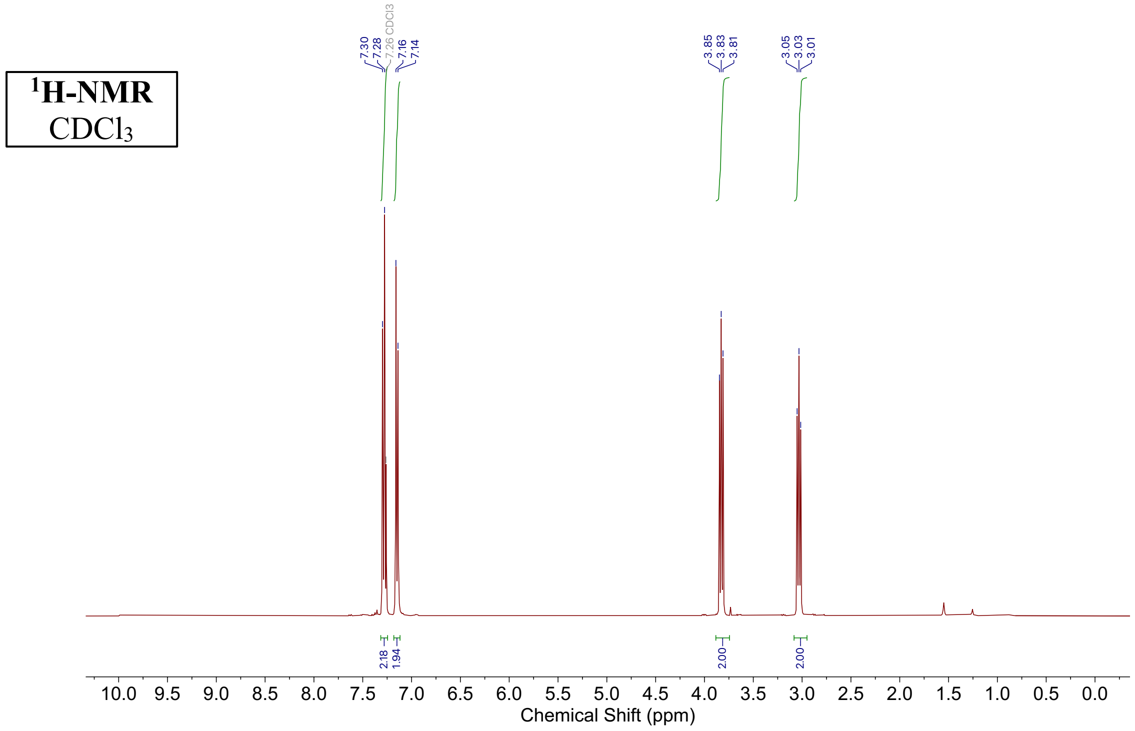

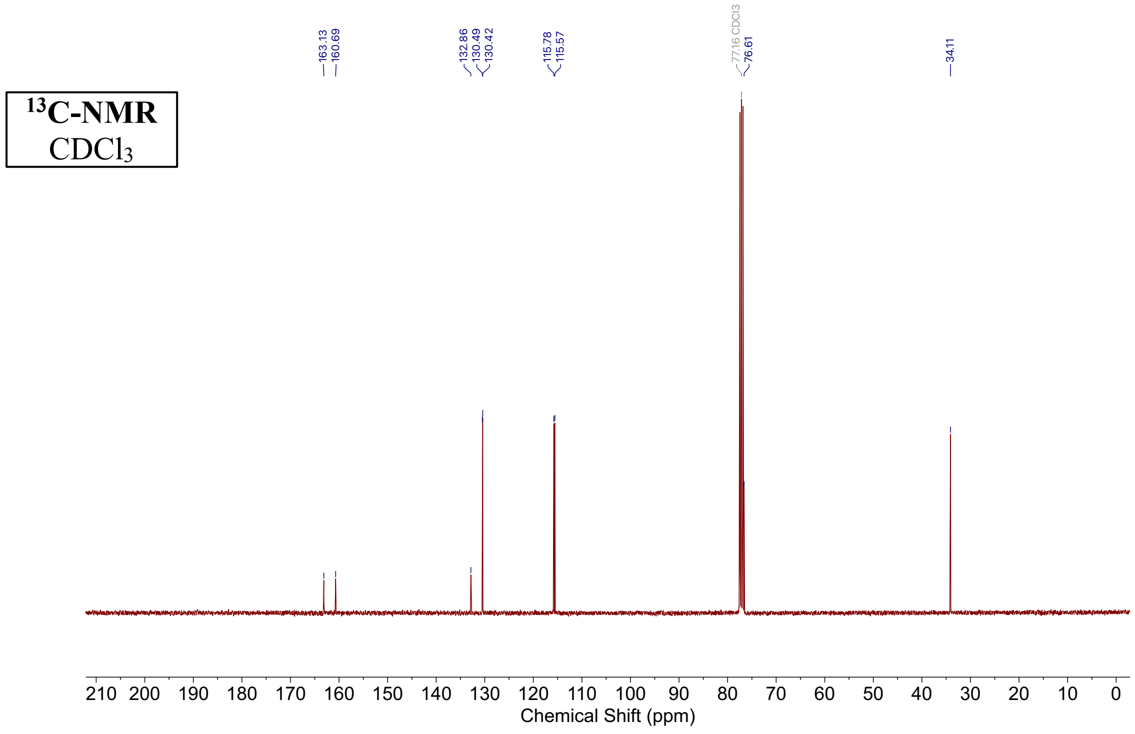

***N,N*-dichloro-2-(3-methoxyphenyl)ethan-1-amine (7)**

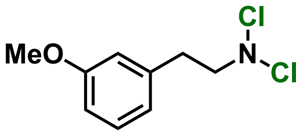

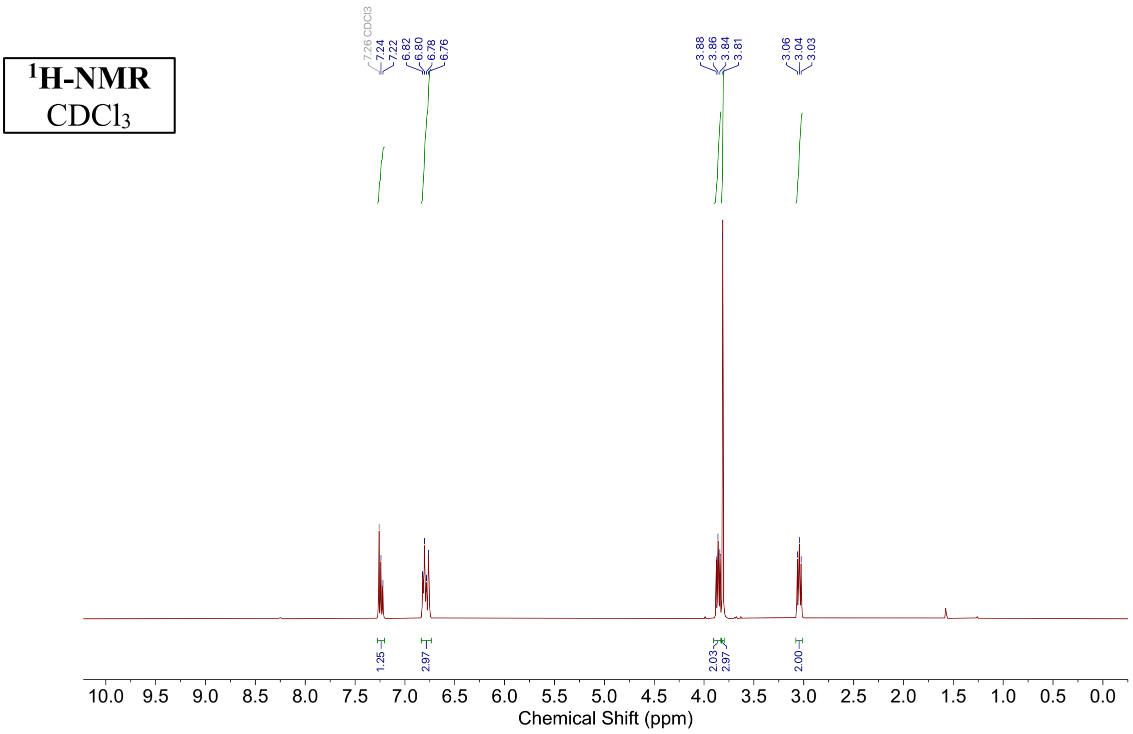

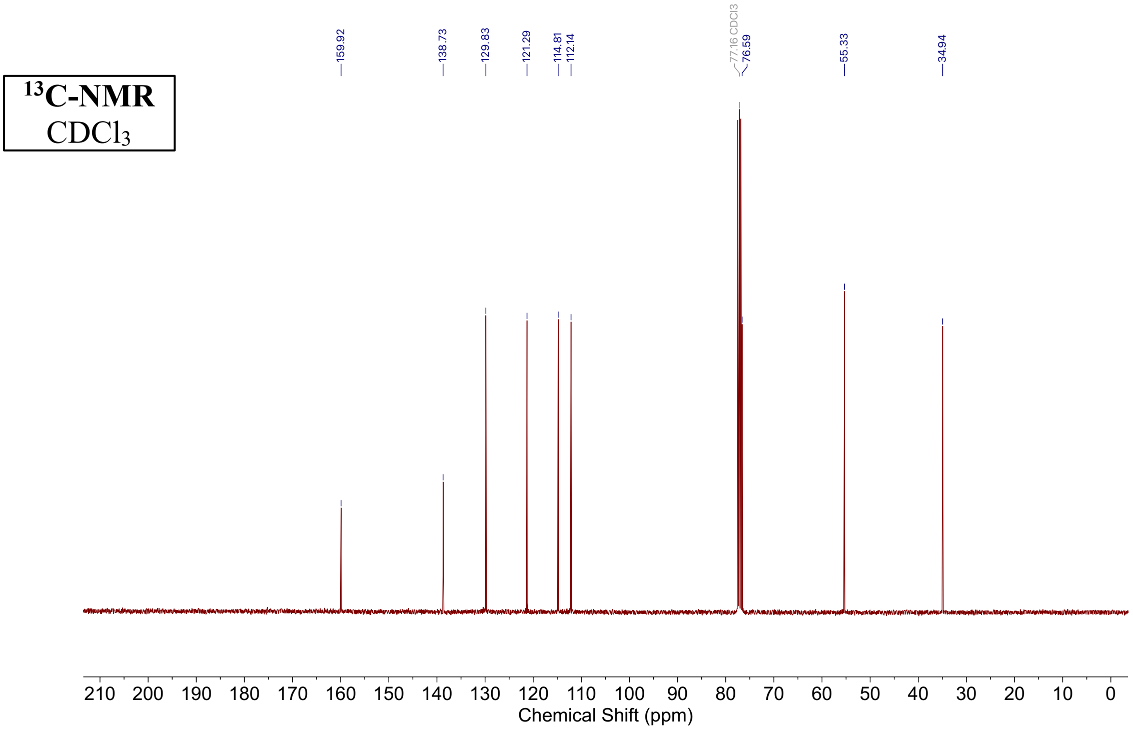

2-(3-bromophenyl)-*N*,*N*-dichloroethan-1-amine (8)

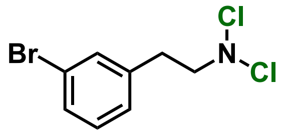

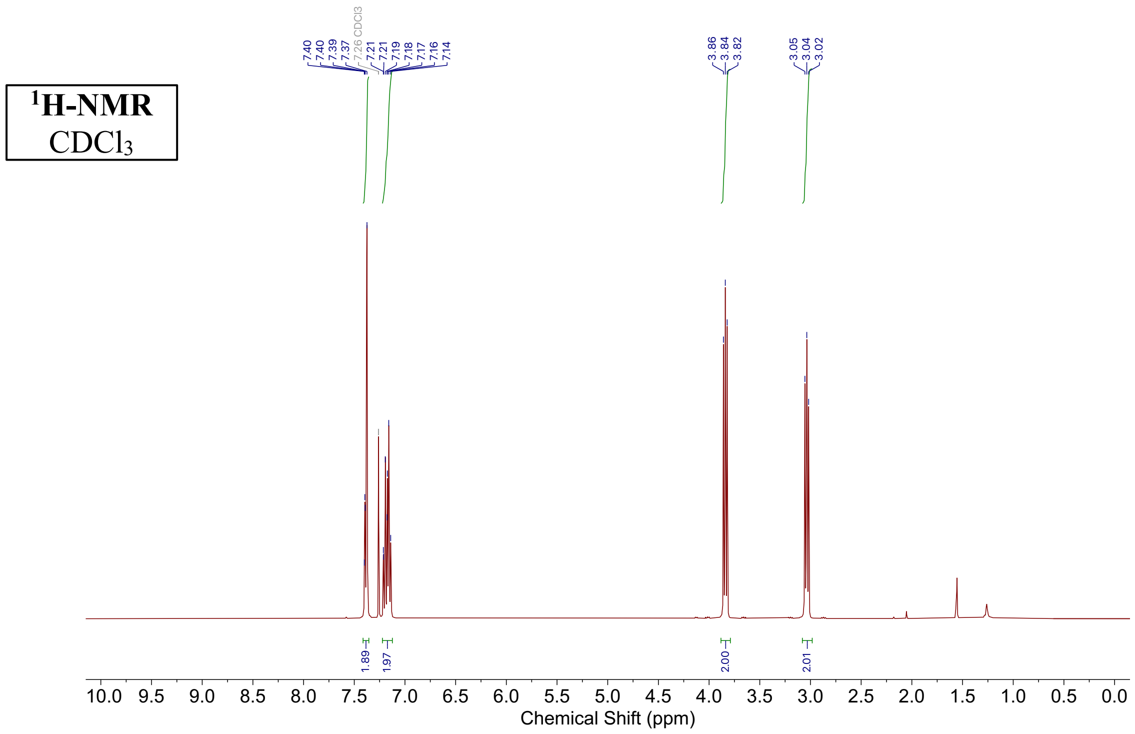

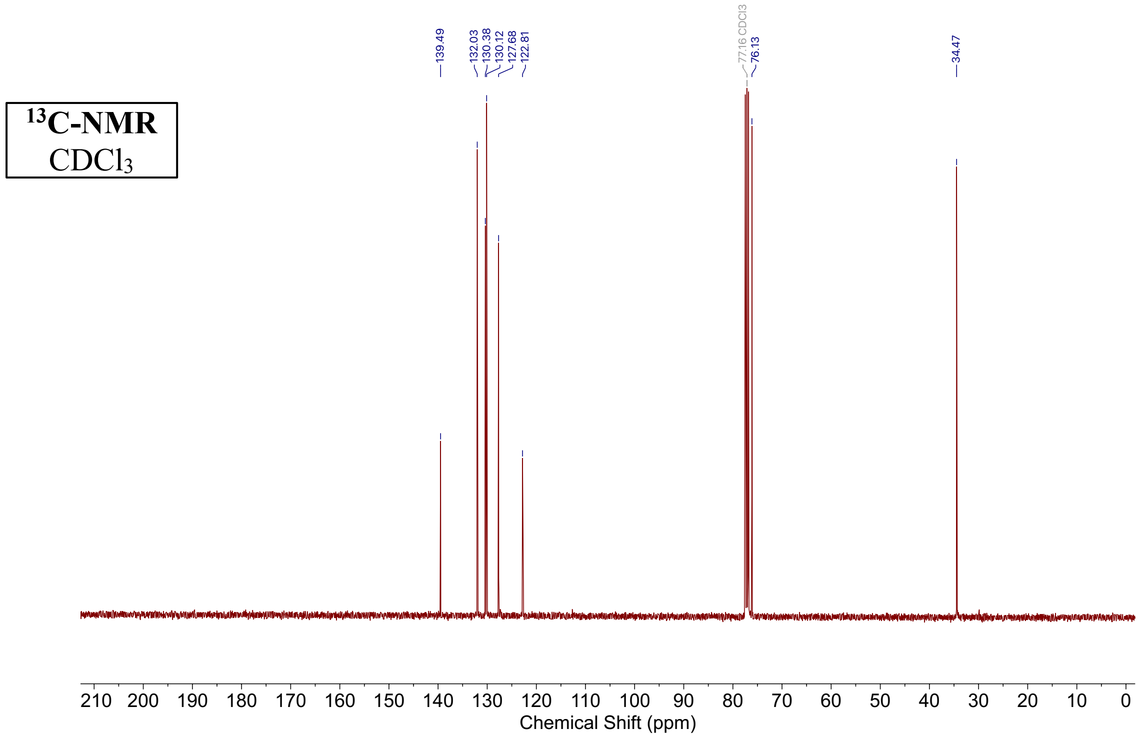

***N,N*-dichloro-2-(3-chlorophenyl)ethan-1-amine (9)**

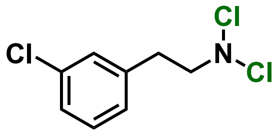

^{1}H-NMR
$CDCl_3$

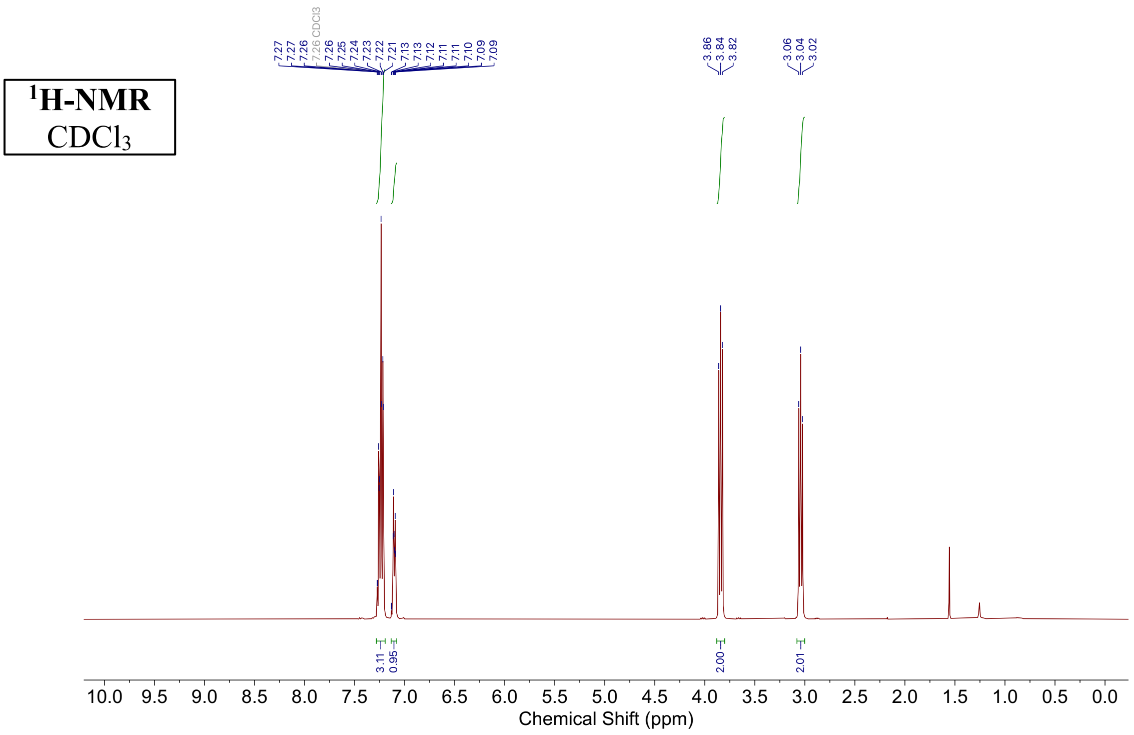

^{13}C-NMR
$CDCl_3$

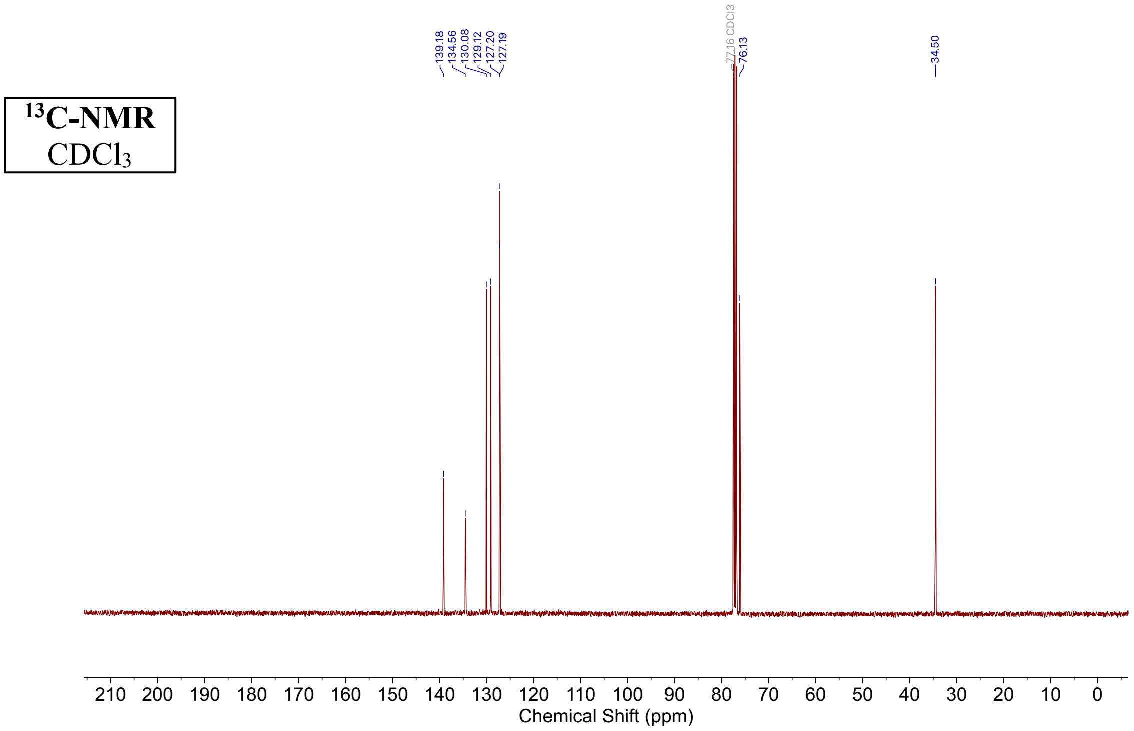

***N,N*-dichloro-2-(3-fluorophenyl)ethan-1-amine (10)**

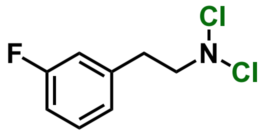

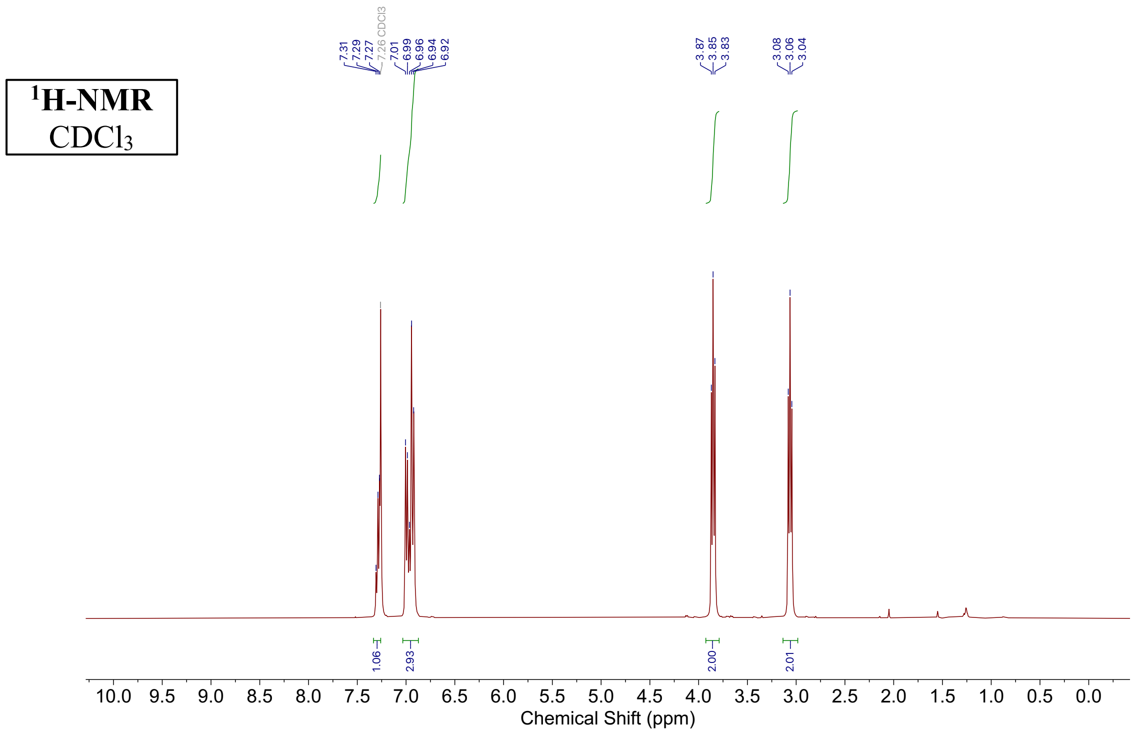

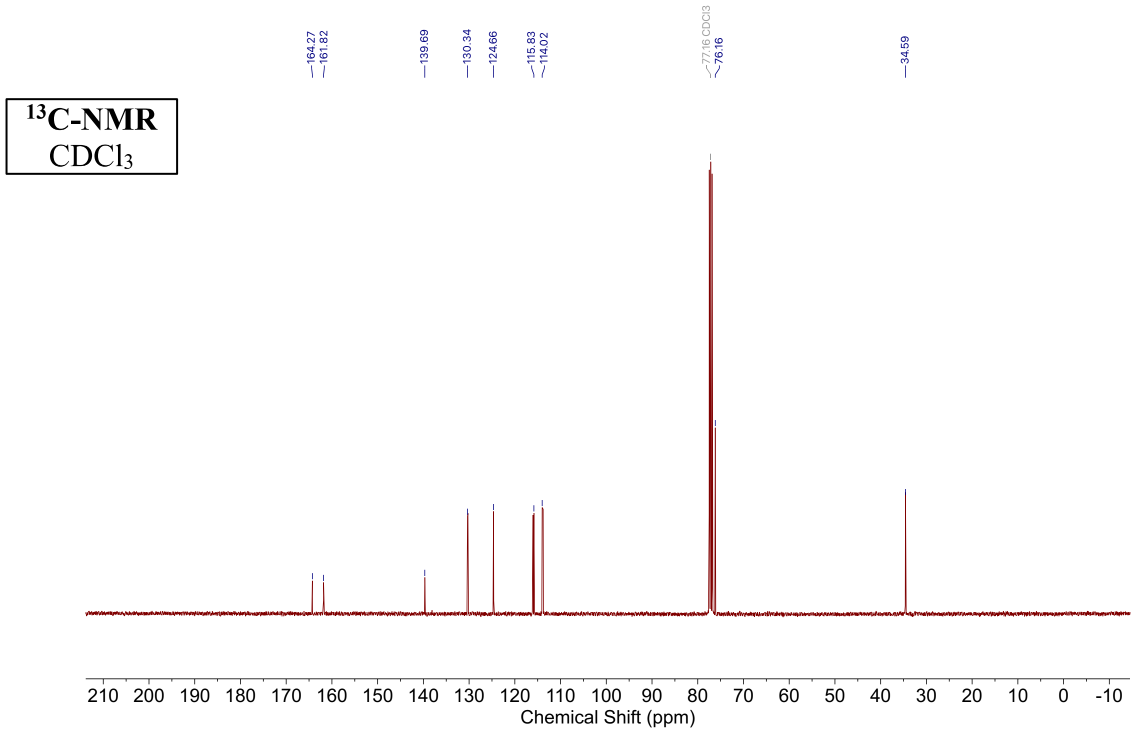

***N,N*-dichloro-2-(2-methoxyphenyl)ethan-1-amine (11)**

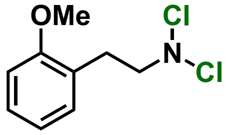

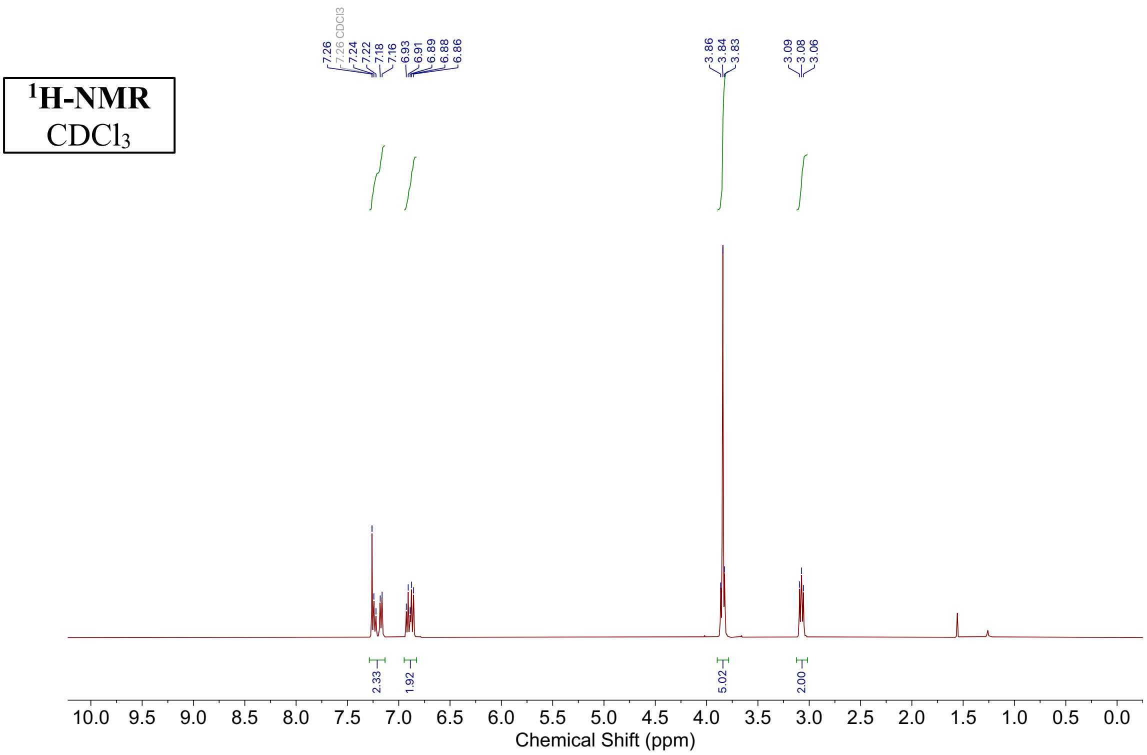

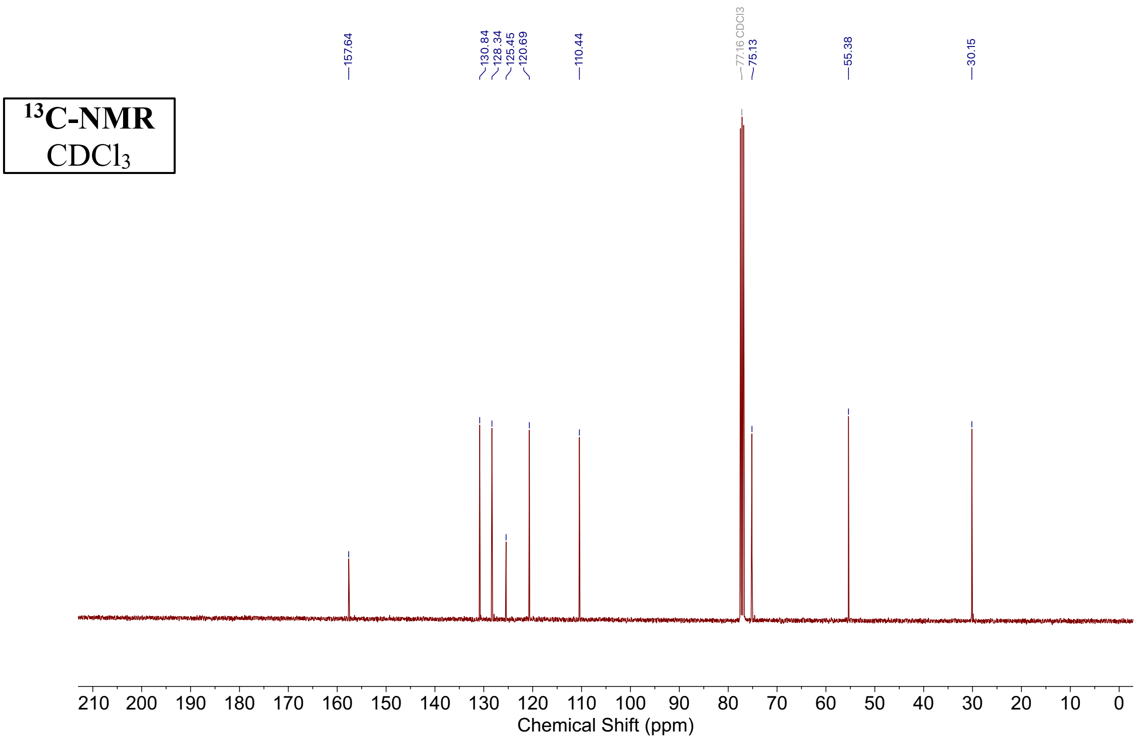

2-(2-bromophenyl)-*N*,*N*-dichloroethan-1-amine (12)

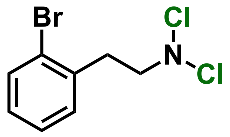

^{1}H-NMR
$CDCl_3$

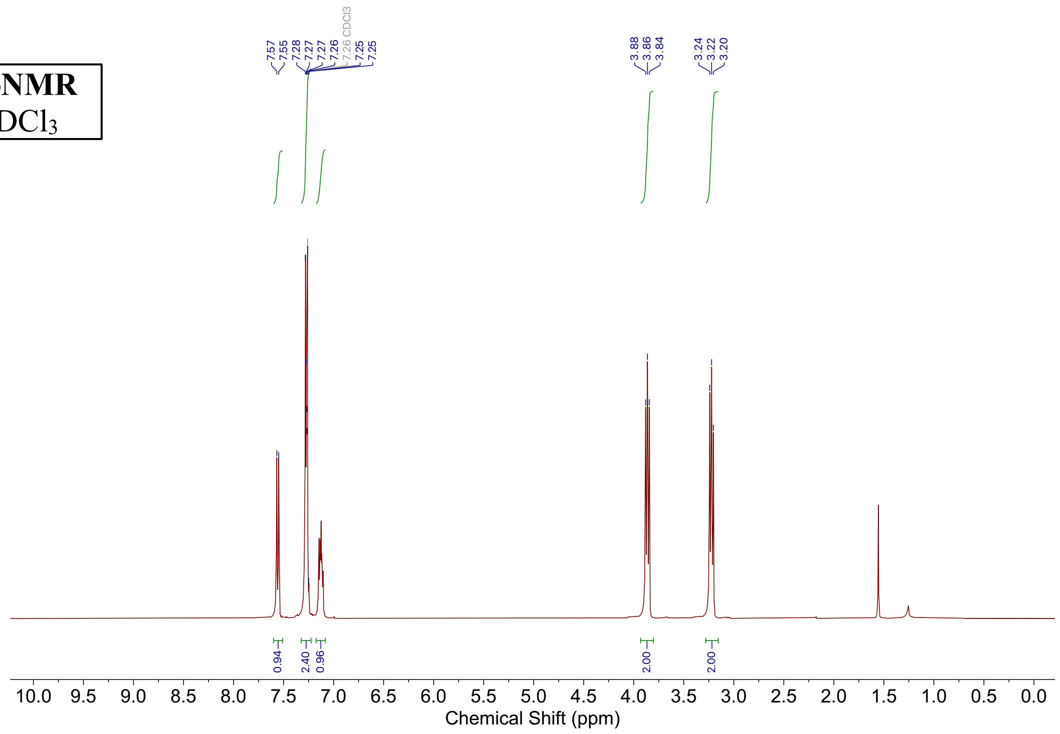

^{13}C-NMR
$CDCl_3$

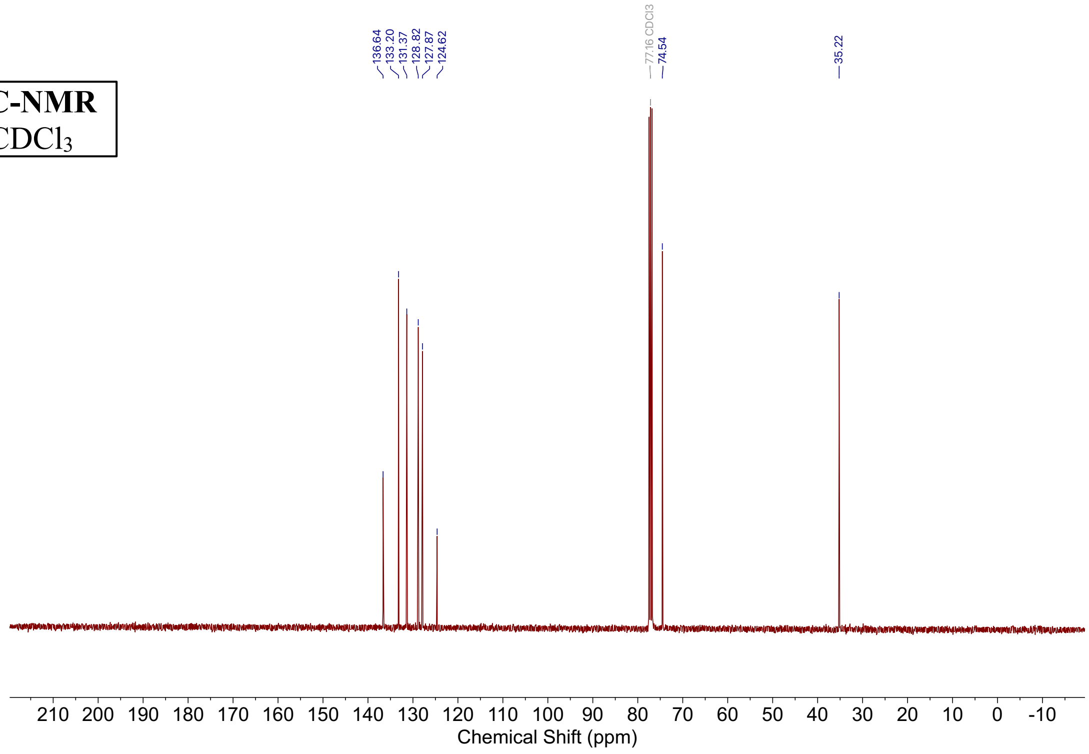

***N,N*-dichloro-2-(2-chlorophenyl)ethan-1-amine (13)**

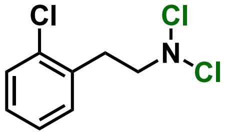

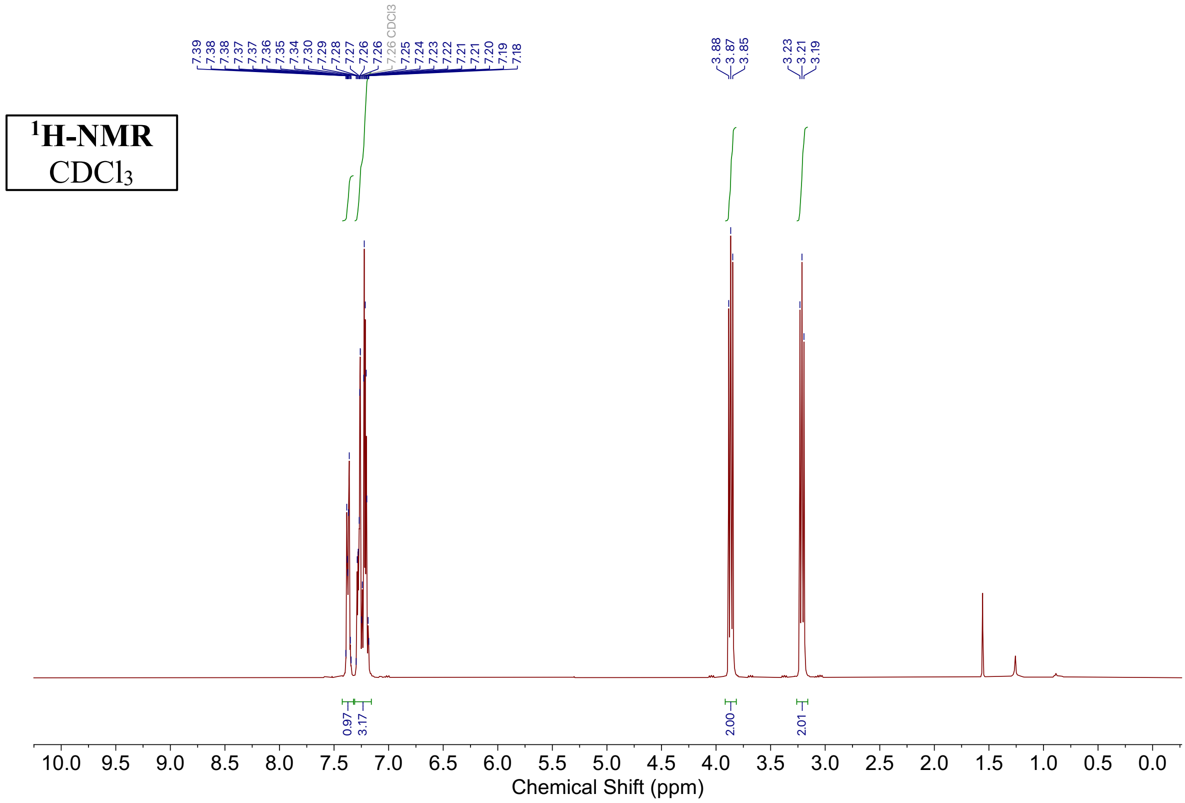

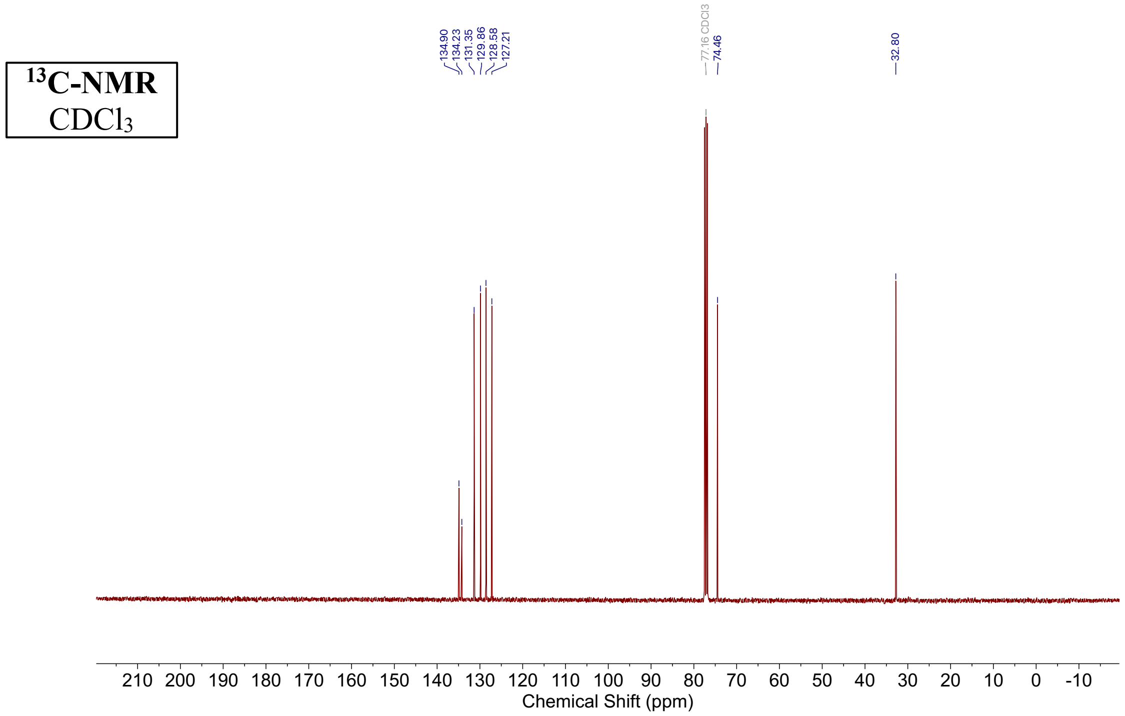

N,N-dichloro-4-phenylbutan-1-amine (14)

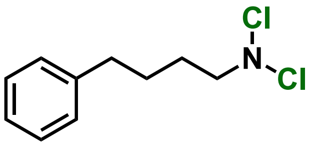

^{1}H-NMR
CDCl$_3$

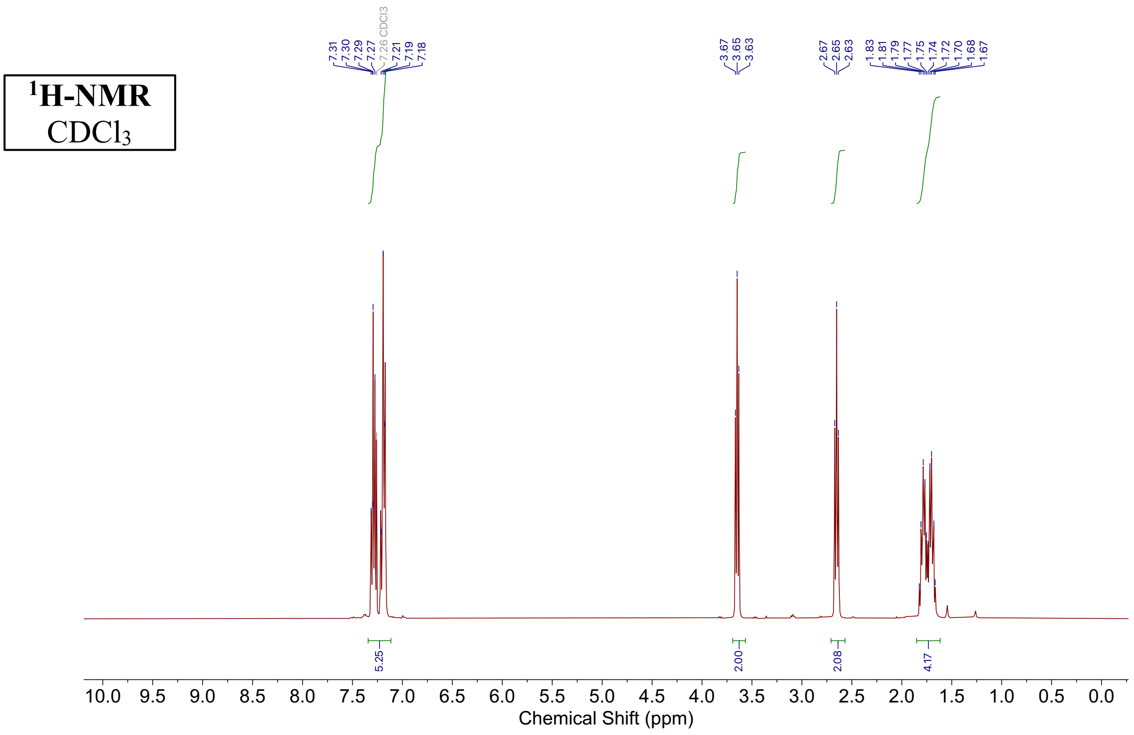

^{13}C-NMR
CDCl$_3$

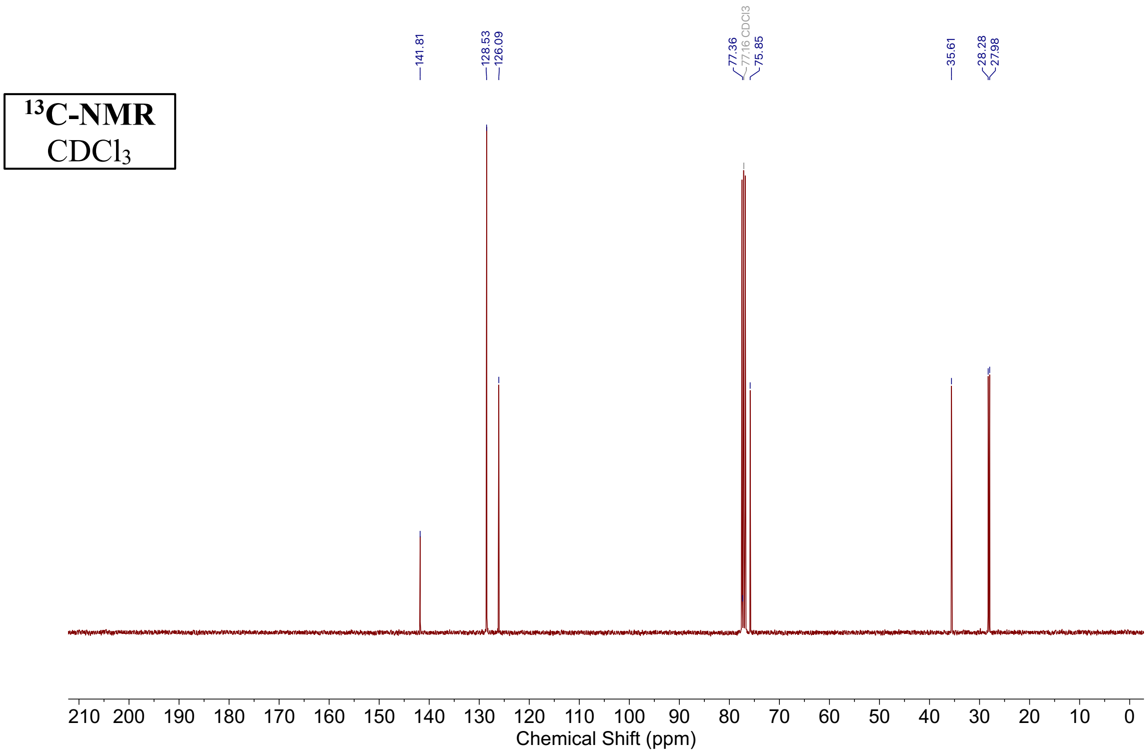

2-(dichloroamino)ethan-1-ol (15)

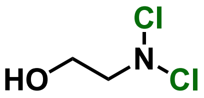

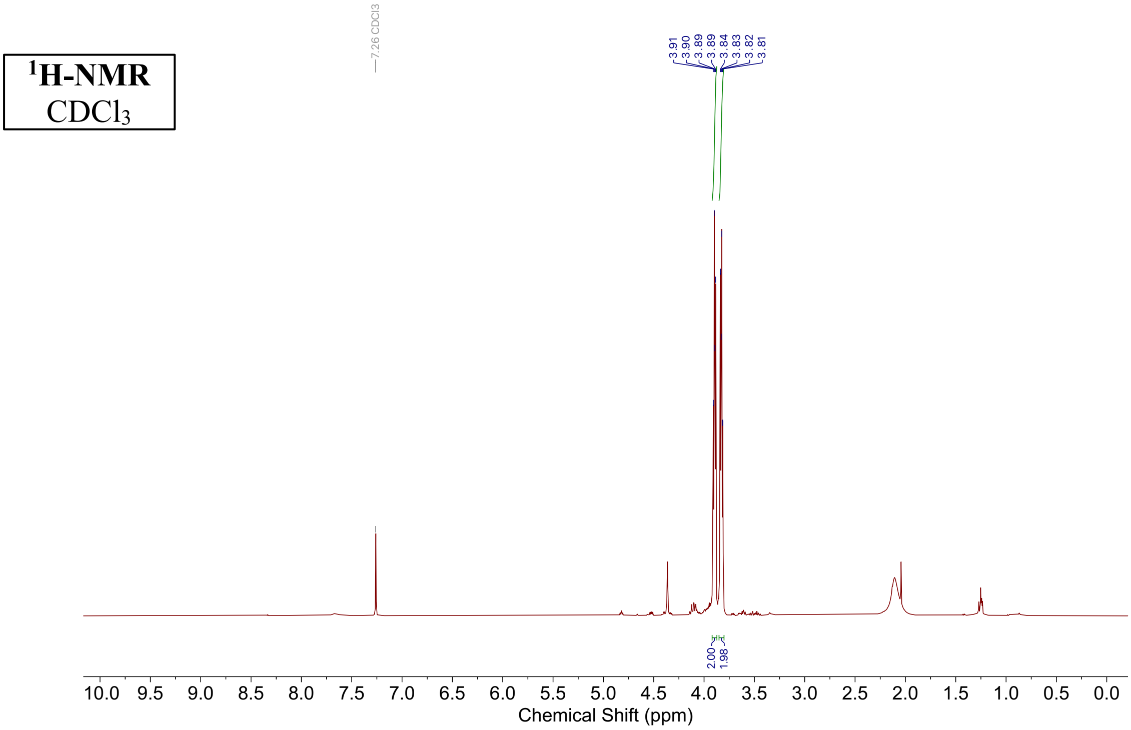

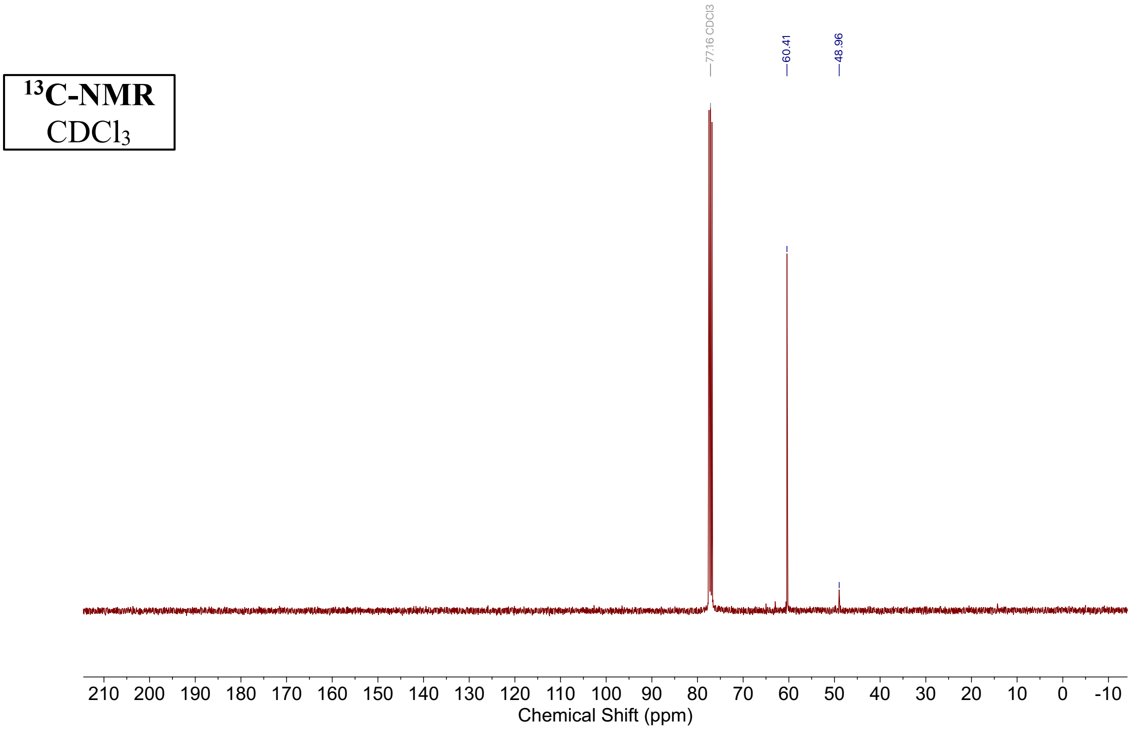

3-cyanopropanoic acid (16)

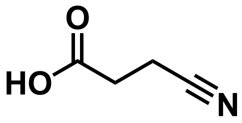

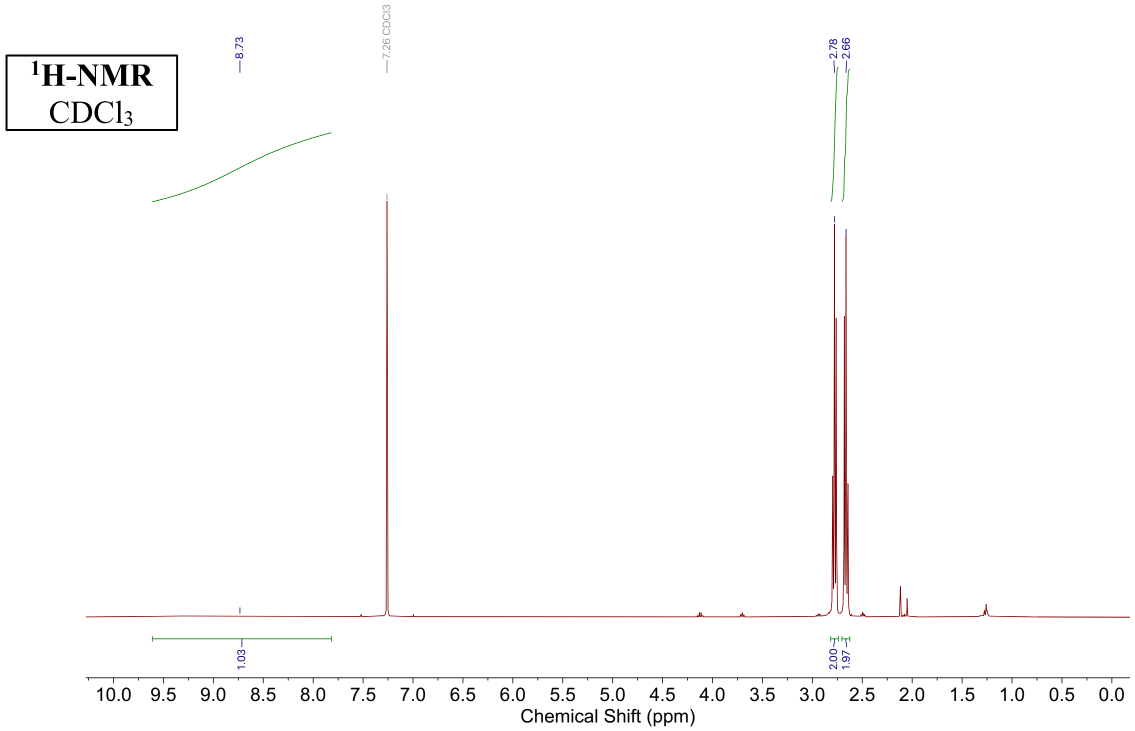

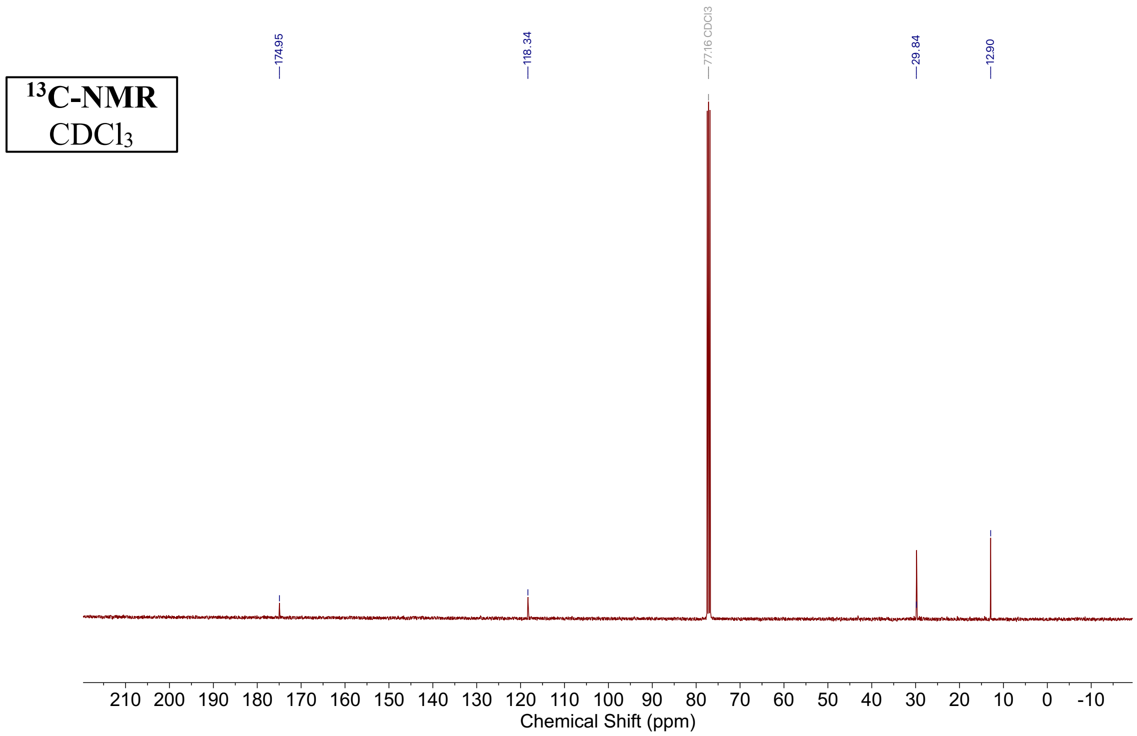

_N_-chloro-_N_-methyl-2-phenylethan-1-amine (17)

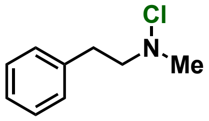

^{1}H-NMR
$CDCl_3$

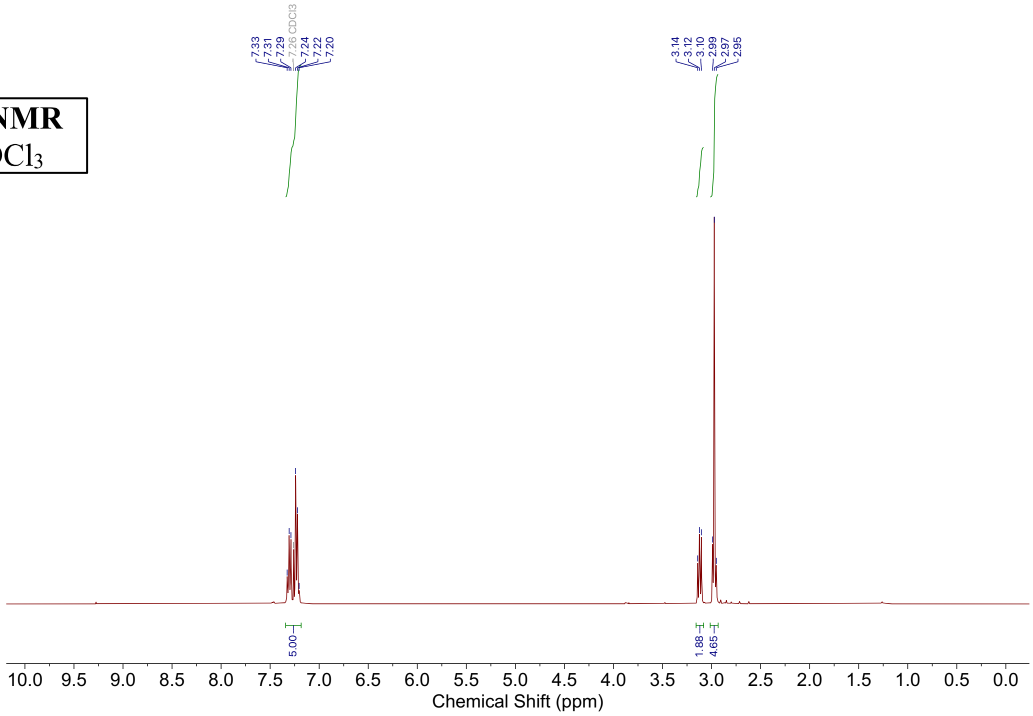

^{13}C-NMR
$CDCl_3$

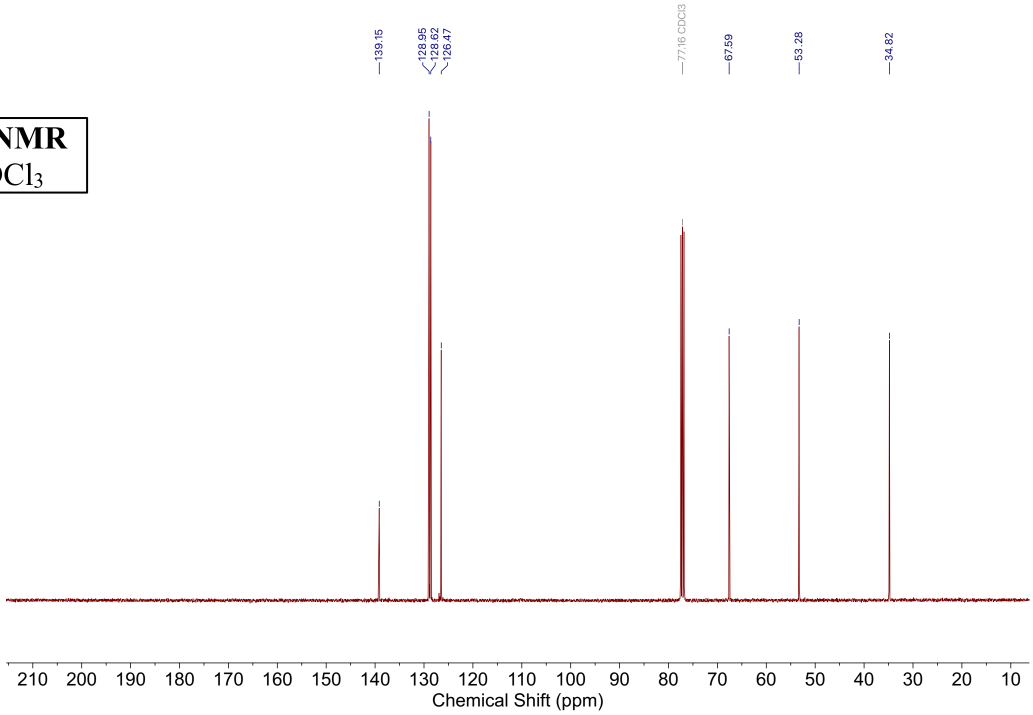

***N*-benzyl-*N*-chloro-1-phenylmethanamine (18)**

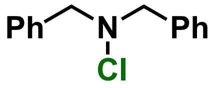

^{1}H-NMR
$CDCl_3$

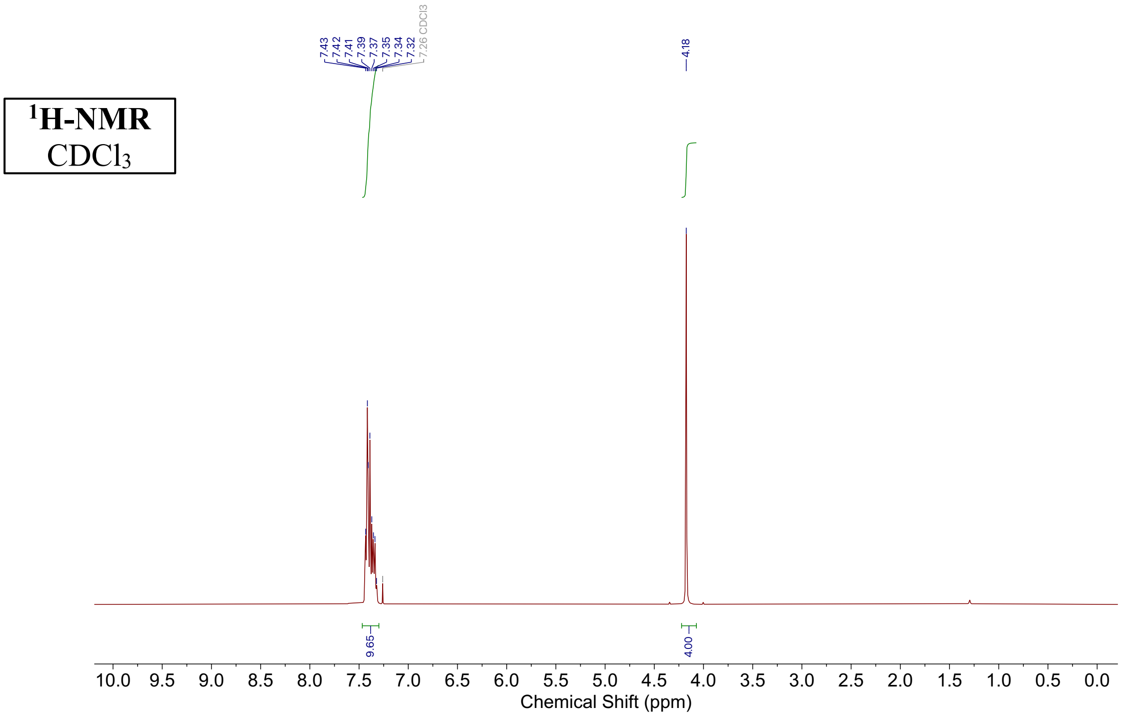

^{13}C-NMR
$CDCl_3$

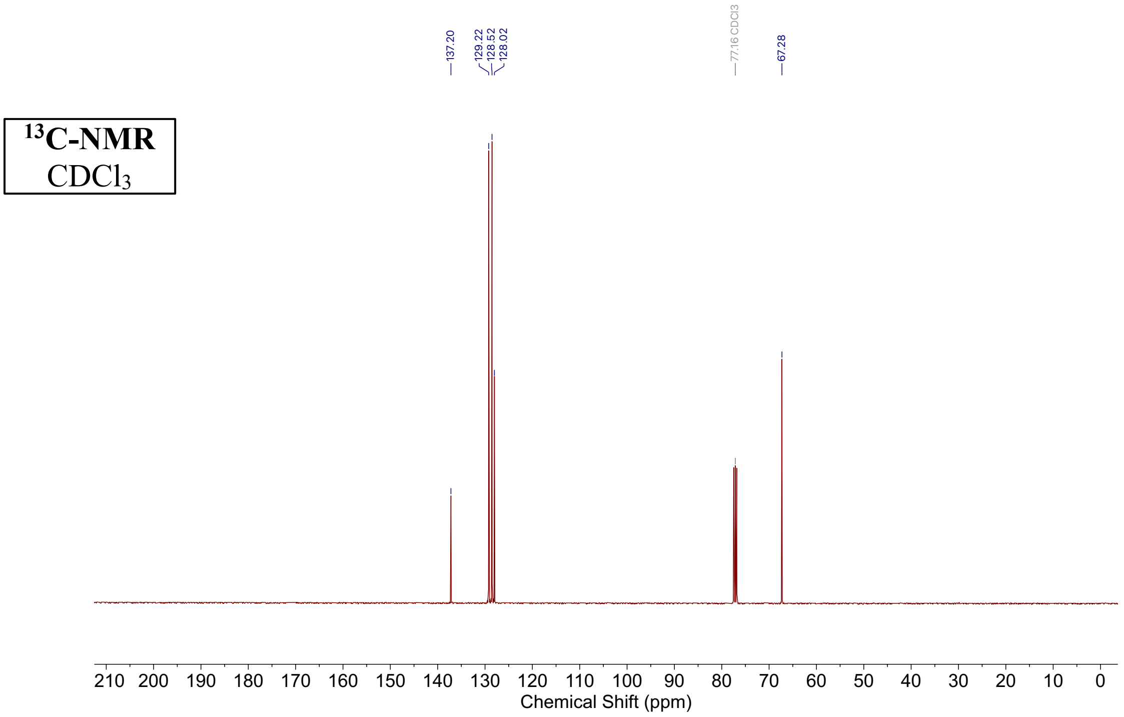

2-(4-(1-chloropiperidin-3-yl)phenyl)-2H-indazole-7-carboxamide (19)

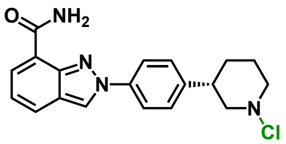

19

^{1}H-NMR
DMSO

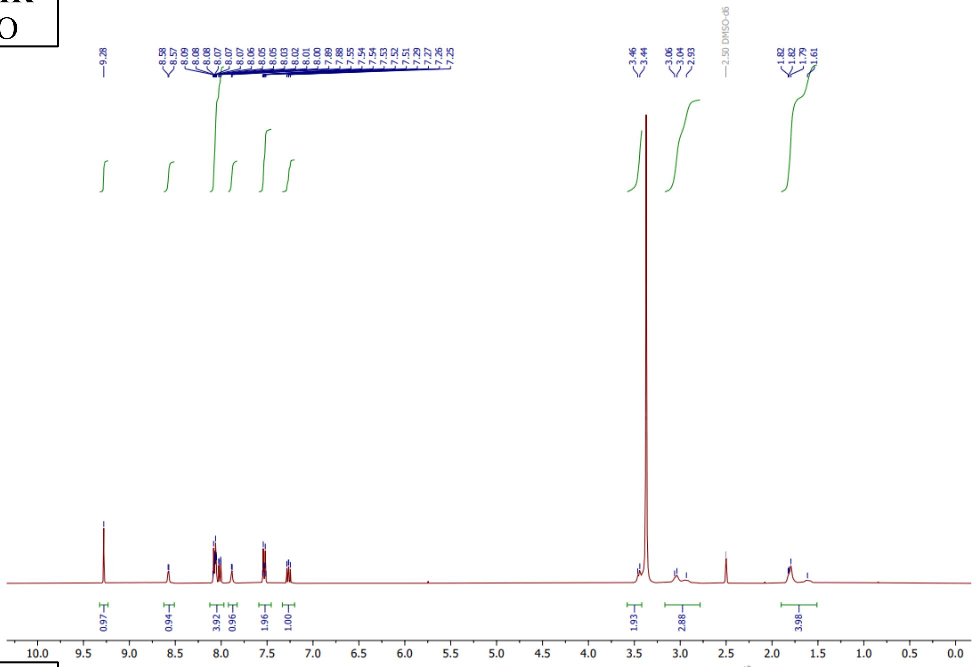

^{13}C-NMR
DMSO

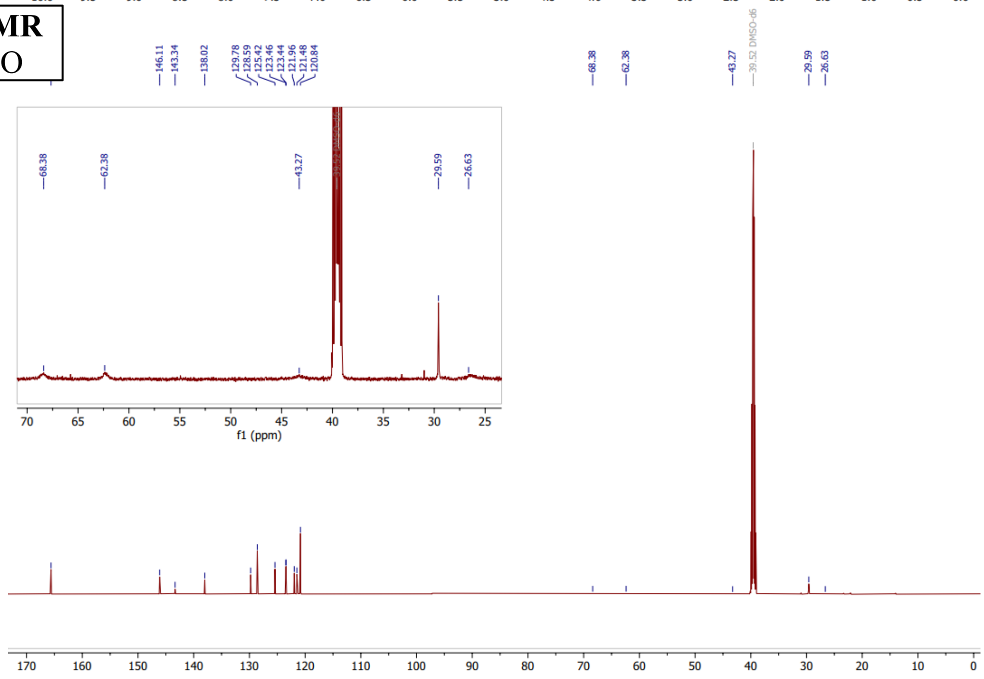

(*E*)-*N*-chloro-1-phenylmethanimine (20)

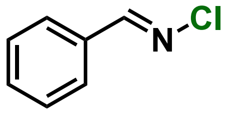

^{1}H-NMR
$CDCl_3$

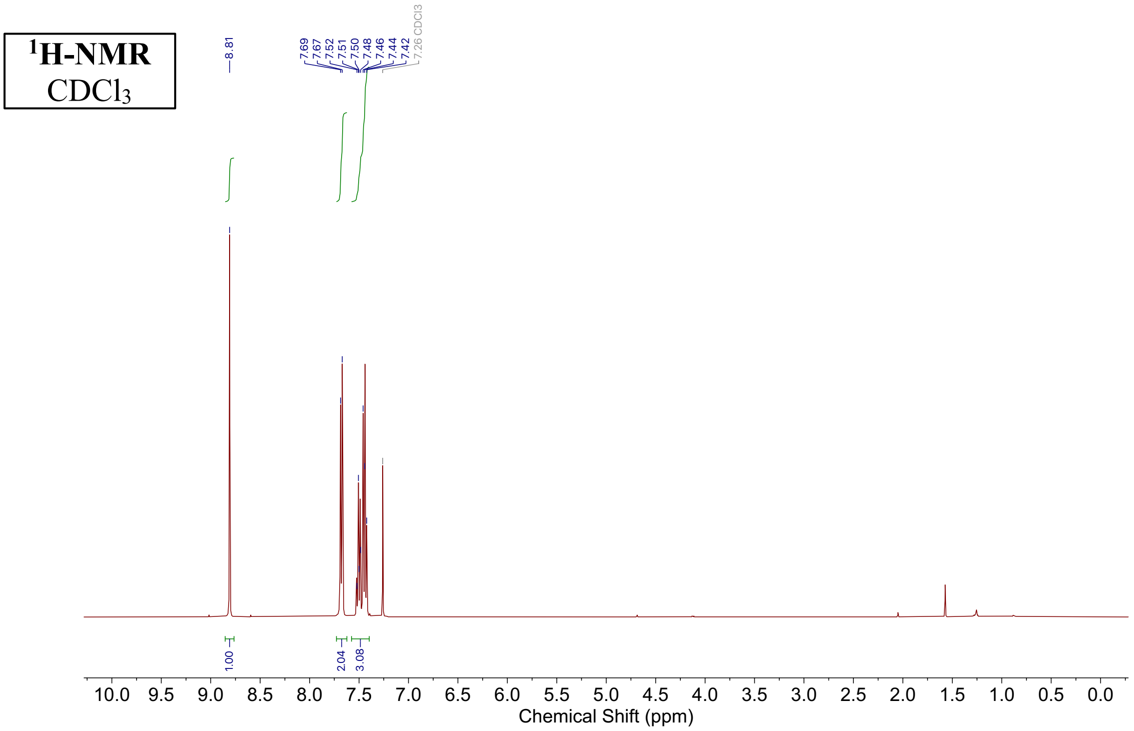

^{13}C-NMR
$CDCl_3$

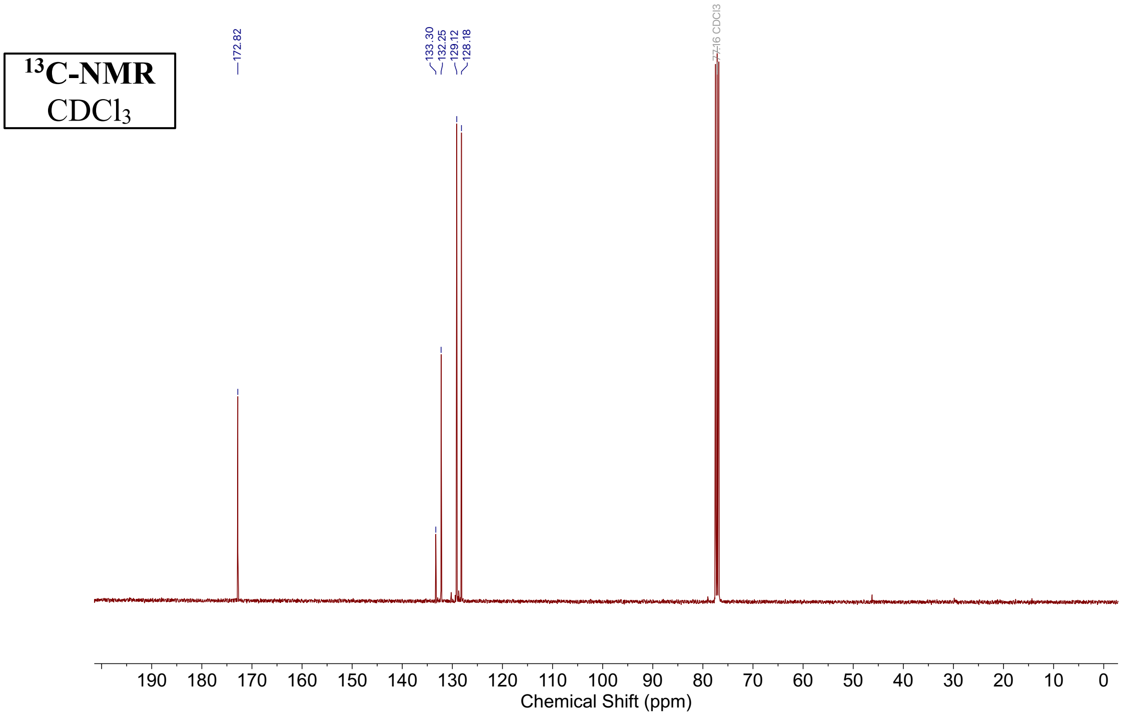

(*E*)-*N*-chloro-1-(4-chlorophenyl)methanimine (21)

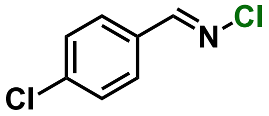

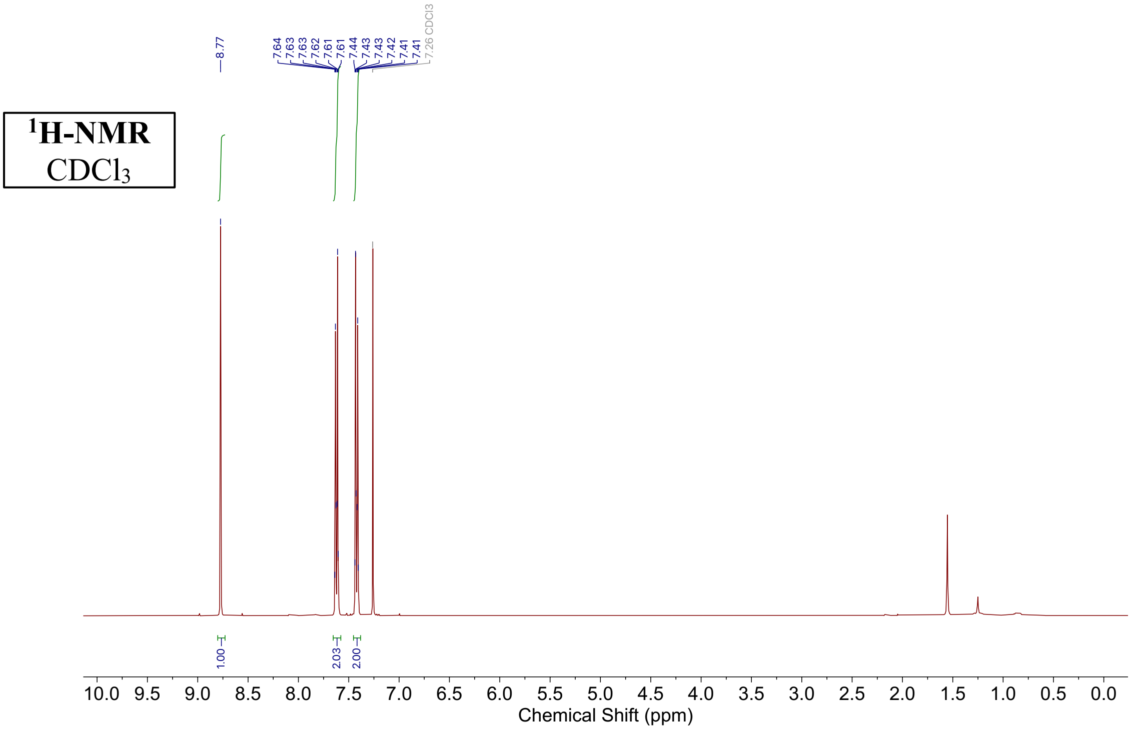

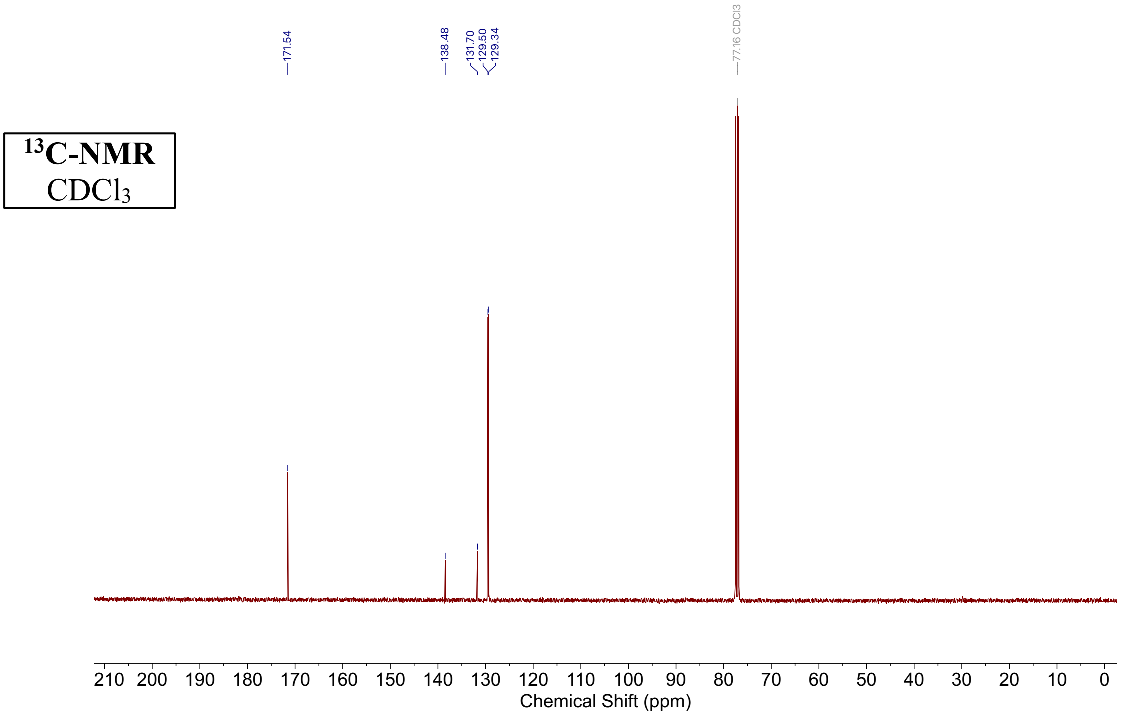

(E)-1-(4-bromophenyl)-N-chloromethanimine (22)

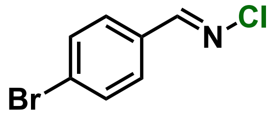

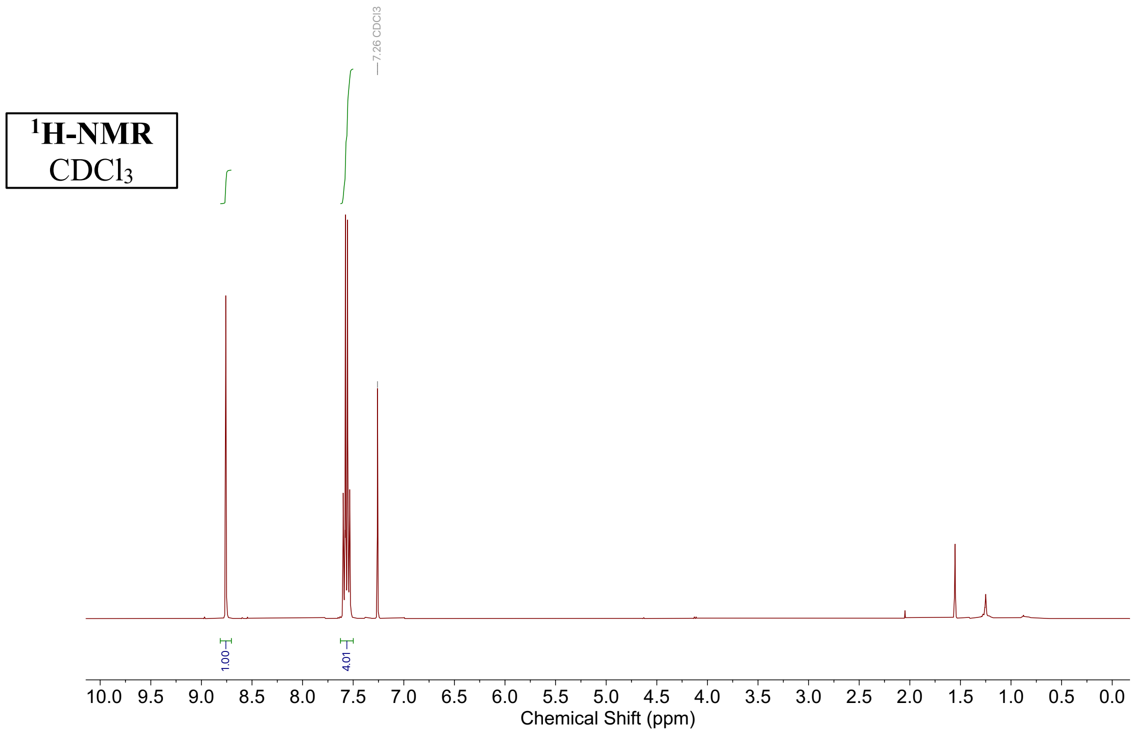

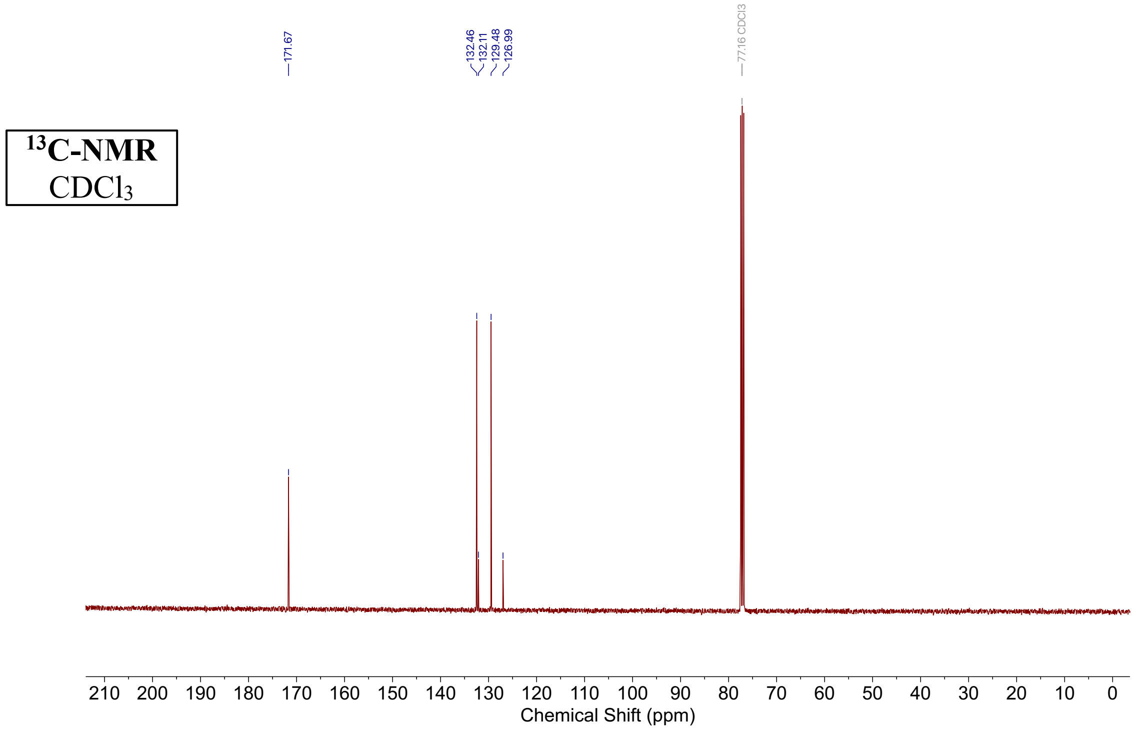

(*E*)-*N*-chloro-1-(4-fluorophenyl)methanimine (23)

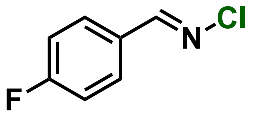

^{1}H-NMR
CDCl$_3$

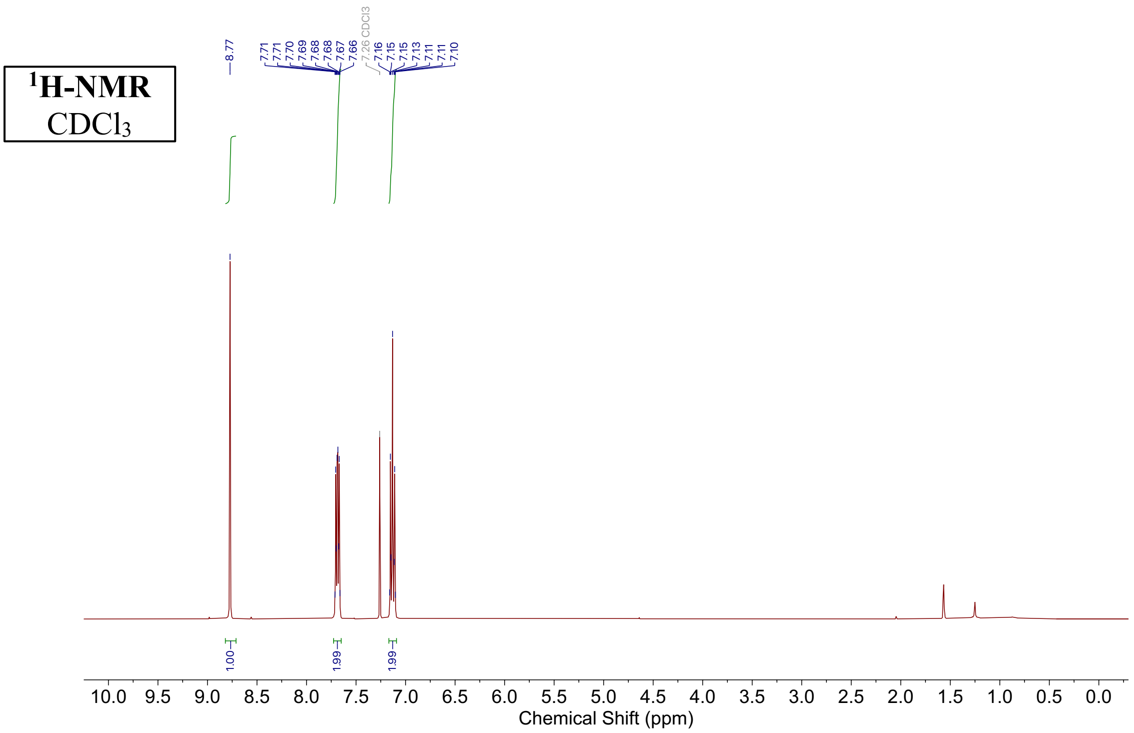

^{13}C-NMR
CDCl$_3$

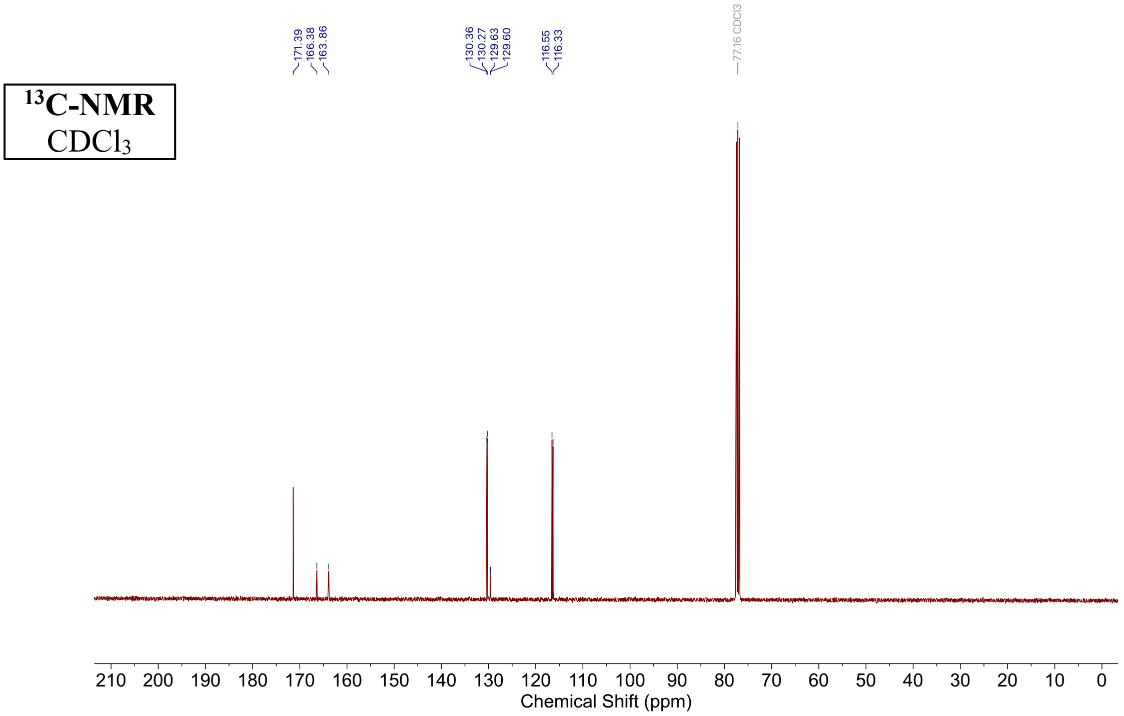

***N*,*N*-dichloro-1-phenylmethanamine (24)**

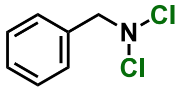

^{1}H-NMR
$CDCl_3$

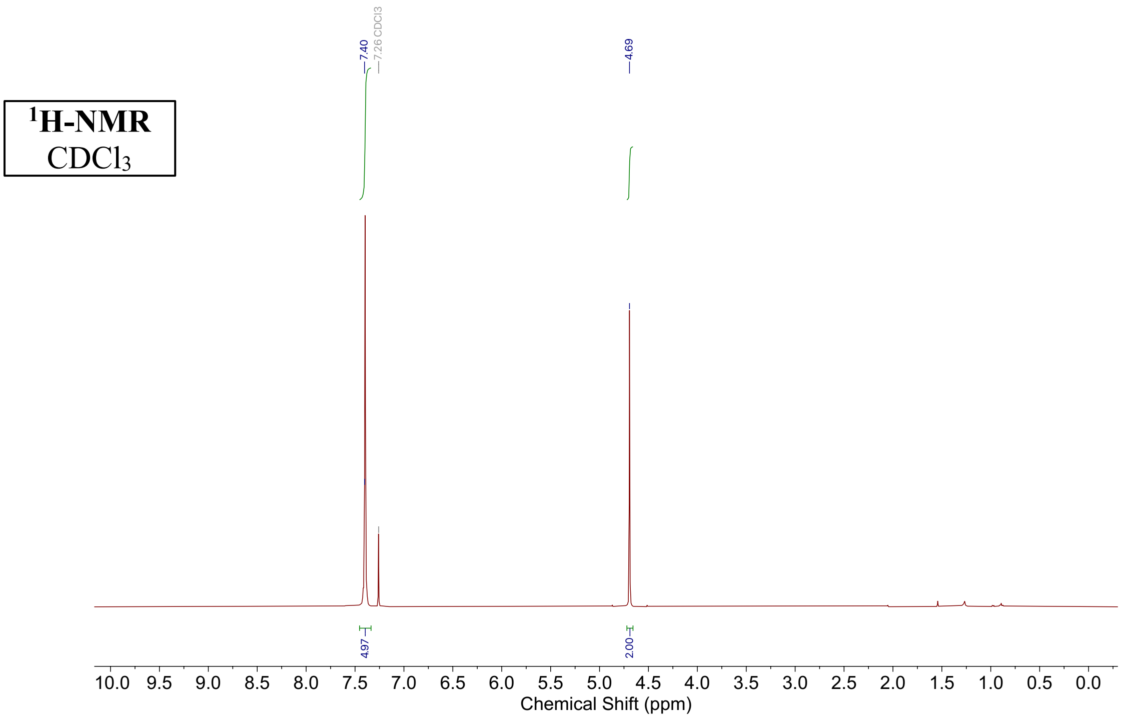

^{13}C-NMR
$CDCl_3$

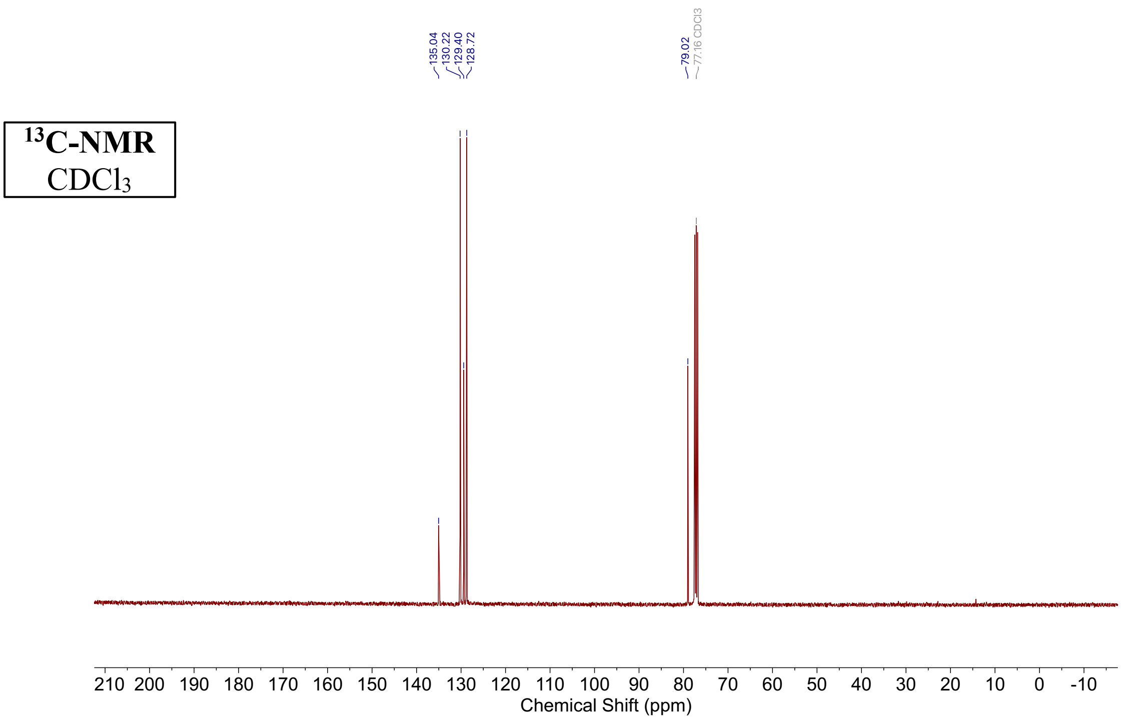

2-phenylacetonitrile (26)

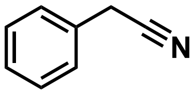

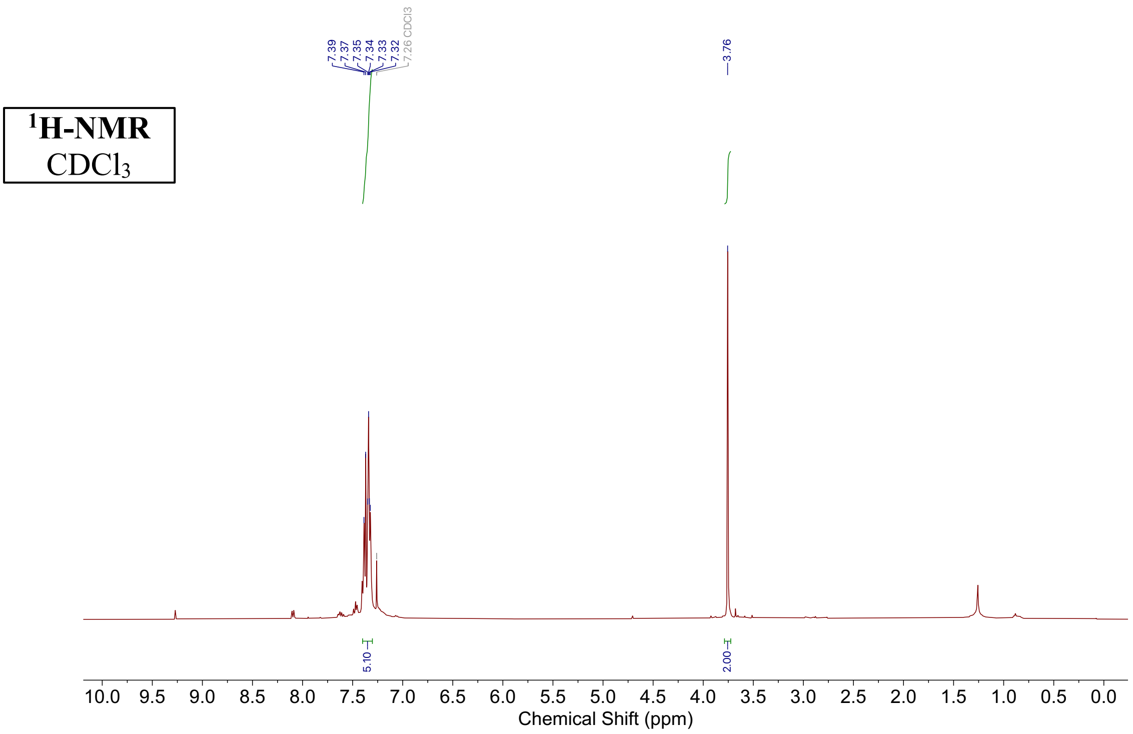

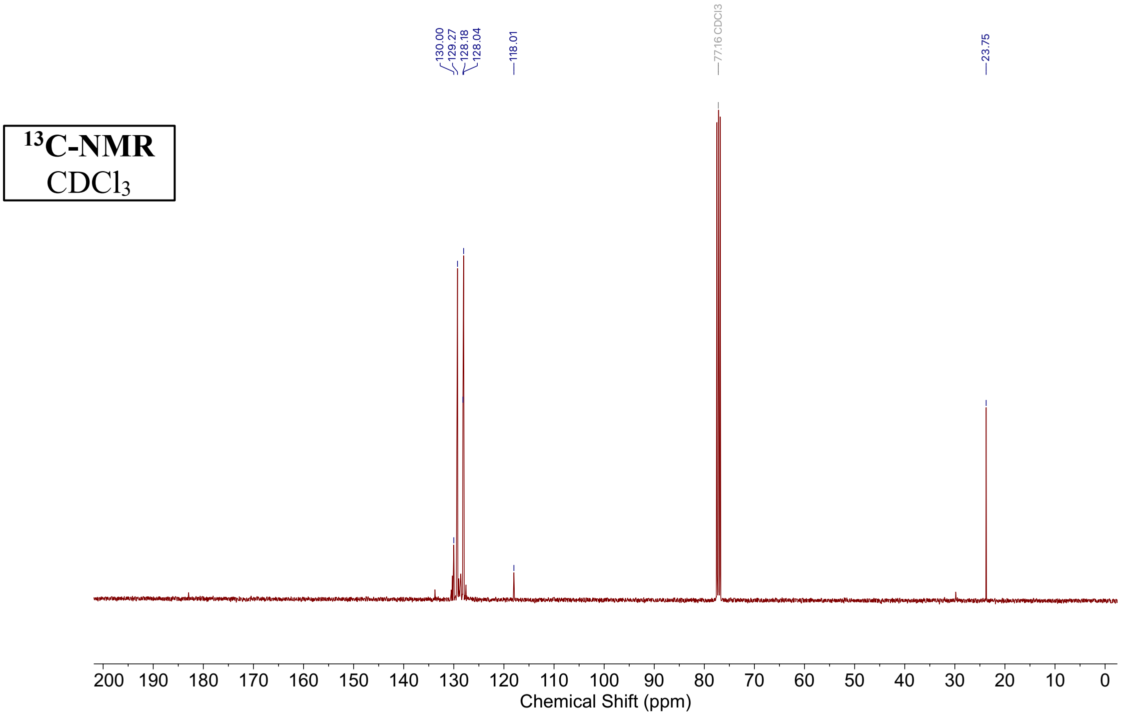

benzonitrile (27)

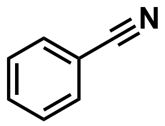

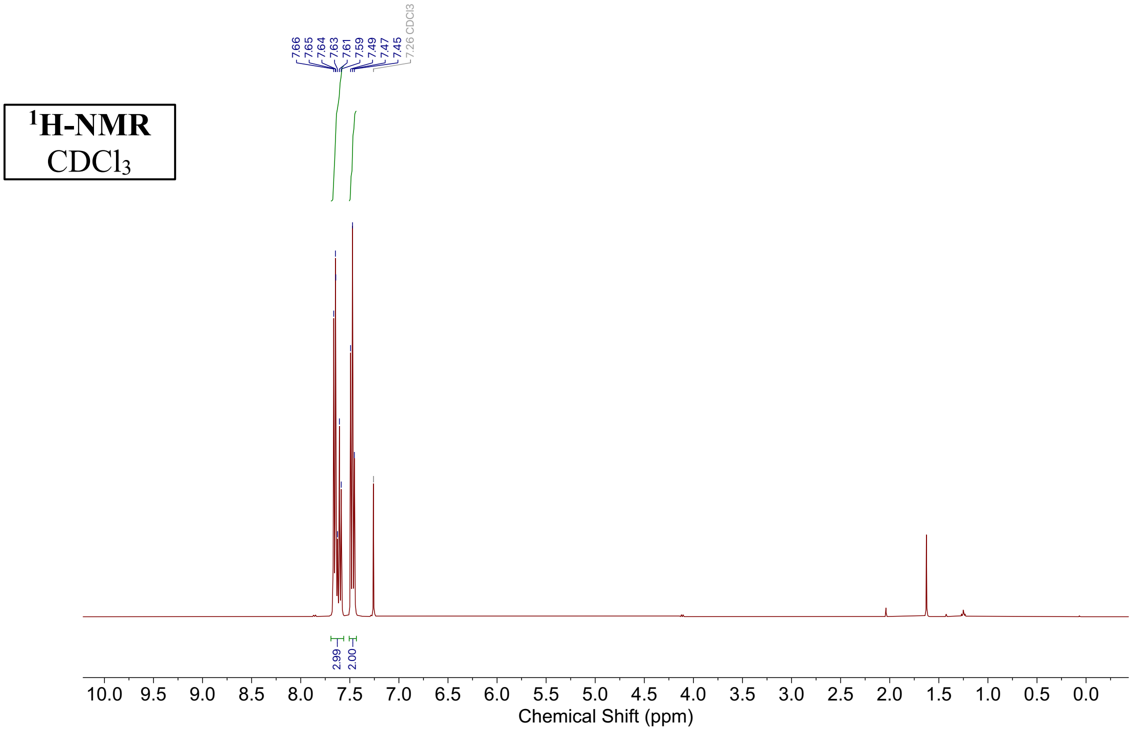

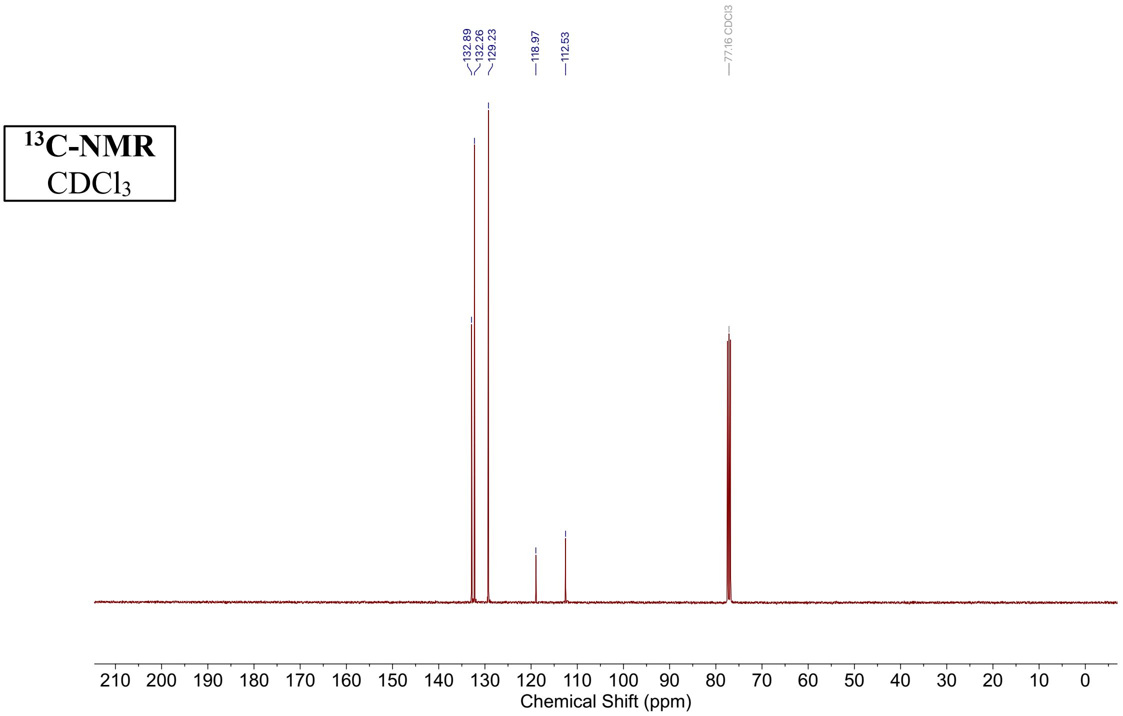

***N*,*N*-dibenzylbenzamide (29)**

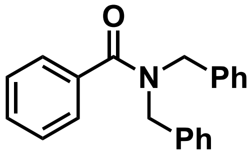

^{1}H-NMR
CDCl$_3$

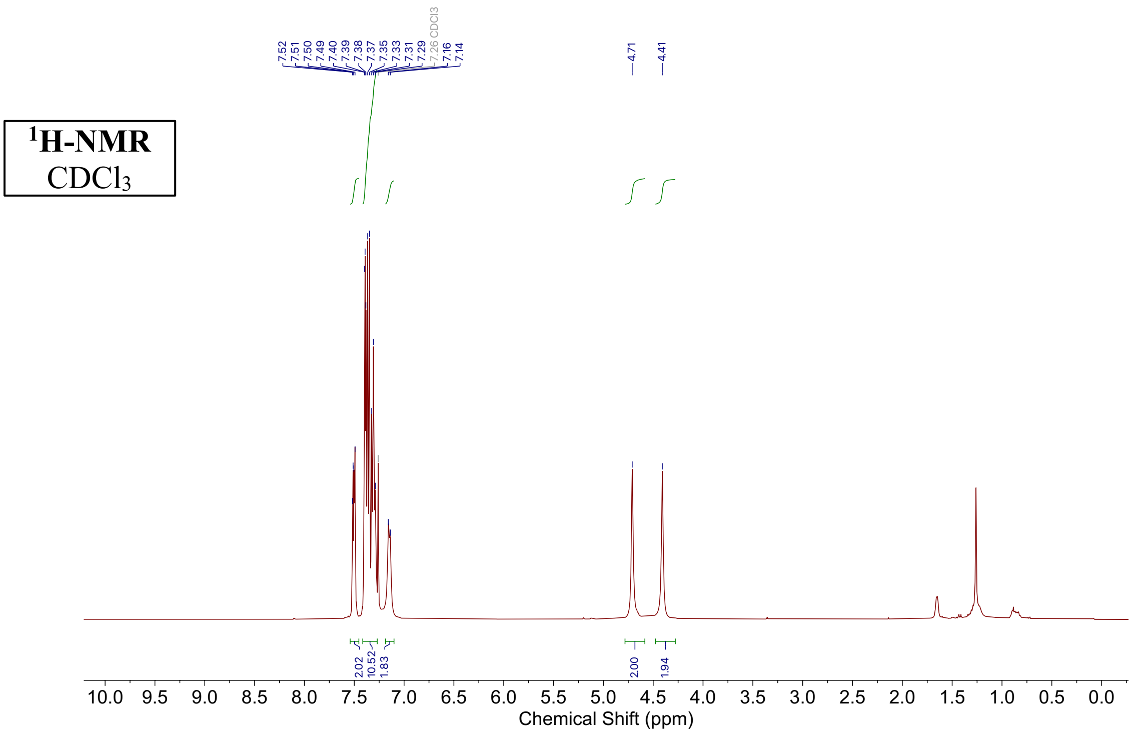

^{13}C-NMR
CDCl$_3$

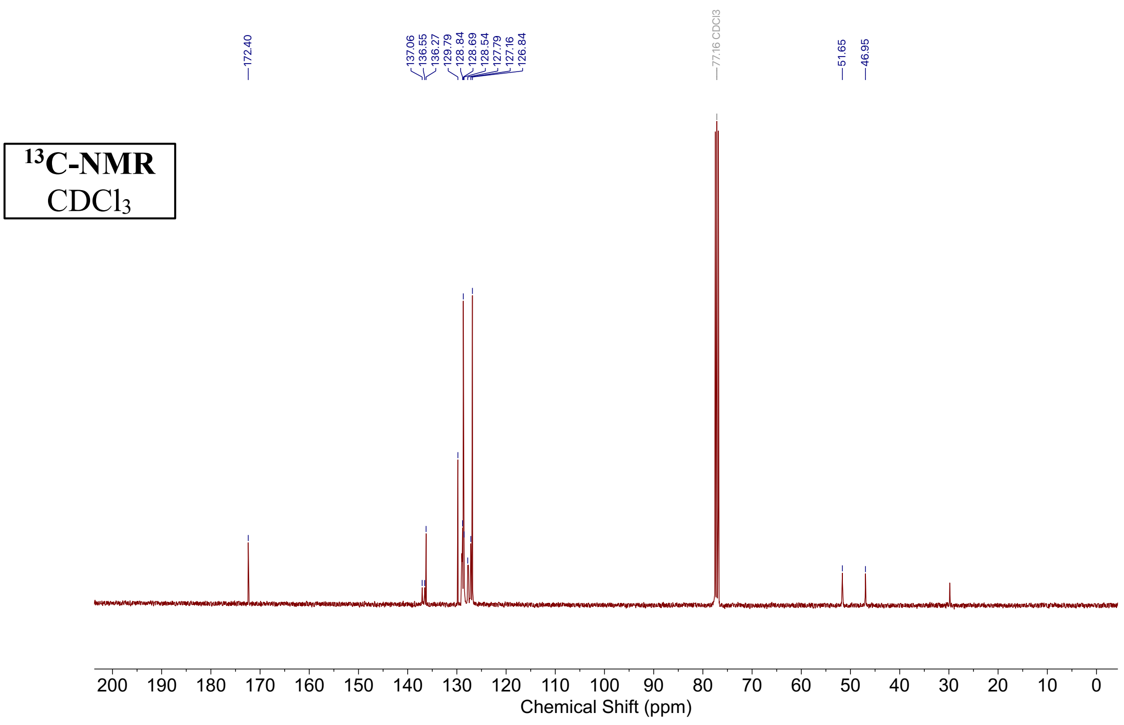

(*E*)-*N*-chloro-1-(naphthalen-2-yl)methanimine (31)

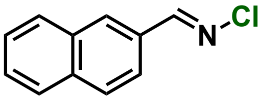

^{1}H-NMR
$CDCl_3$

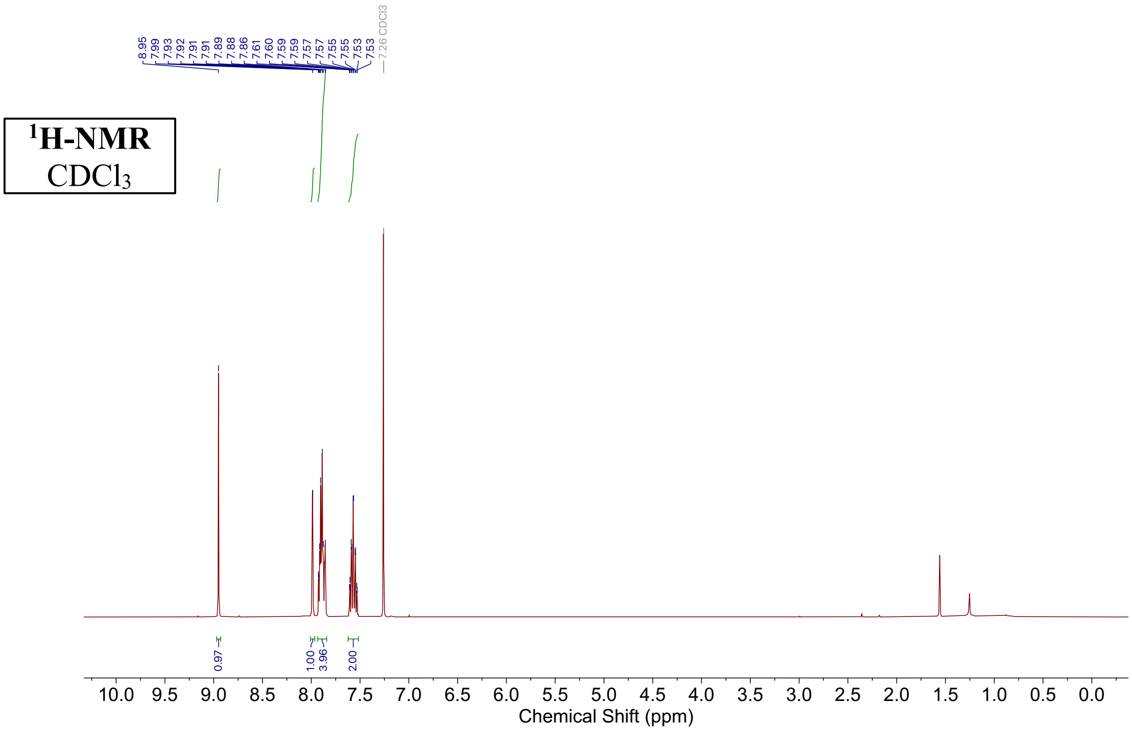

^{13}C-NMR
$CDCl_3$

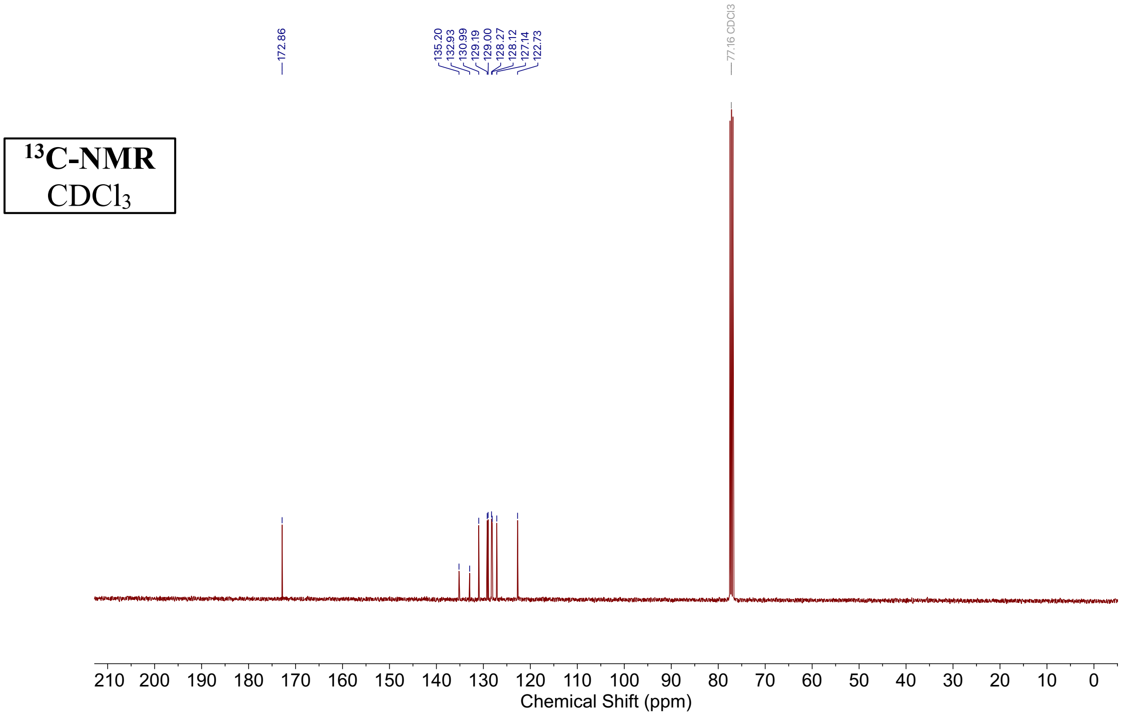

2-naphthaldehyde (32)

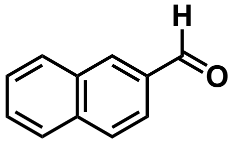

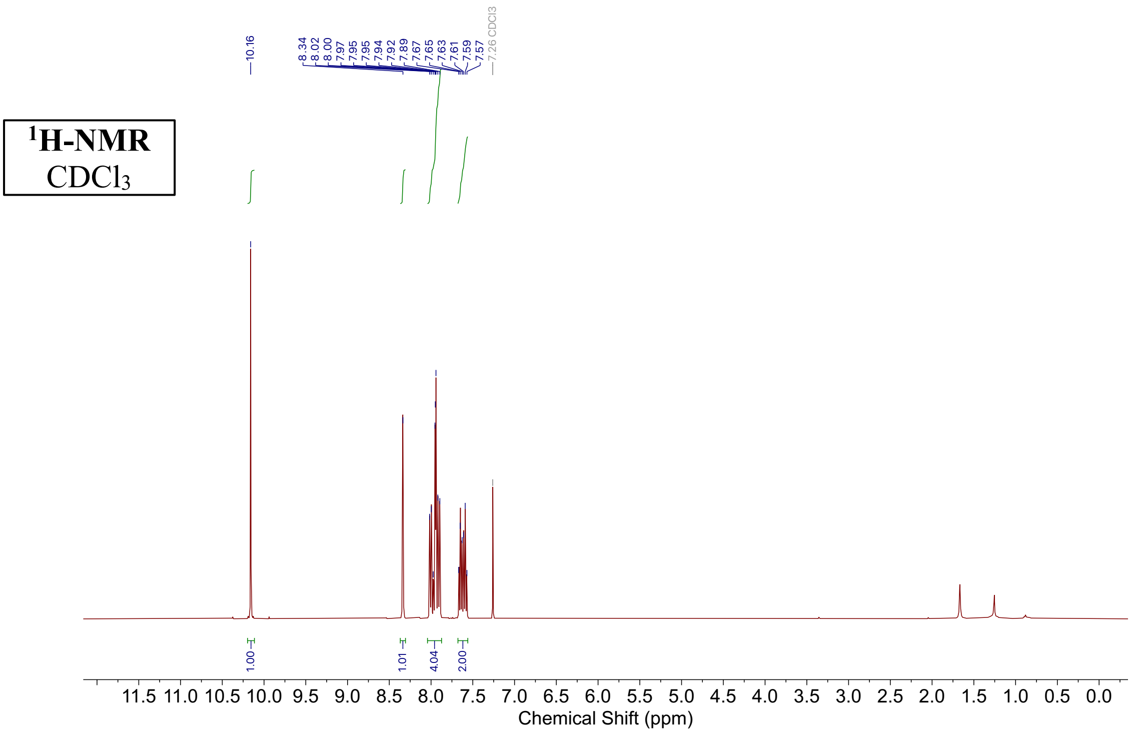

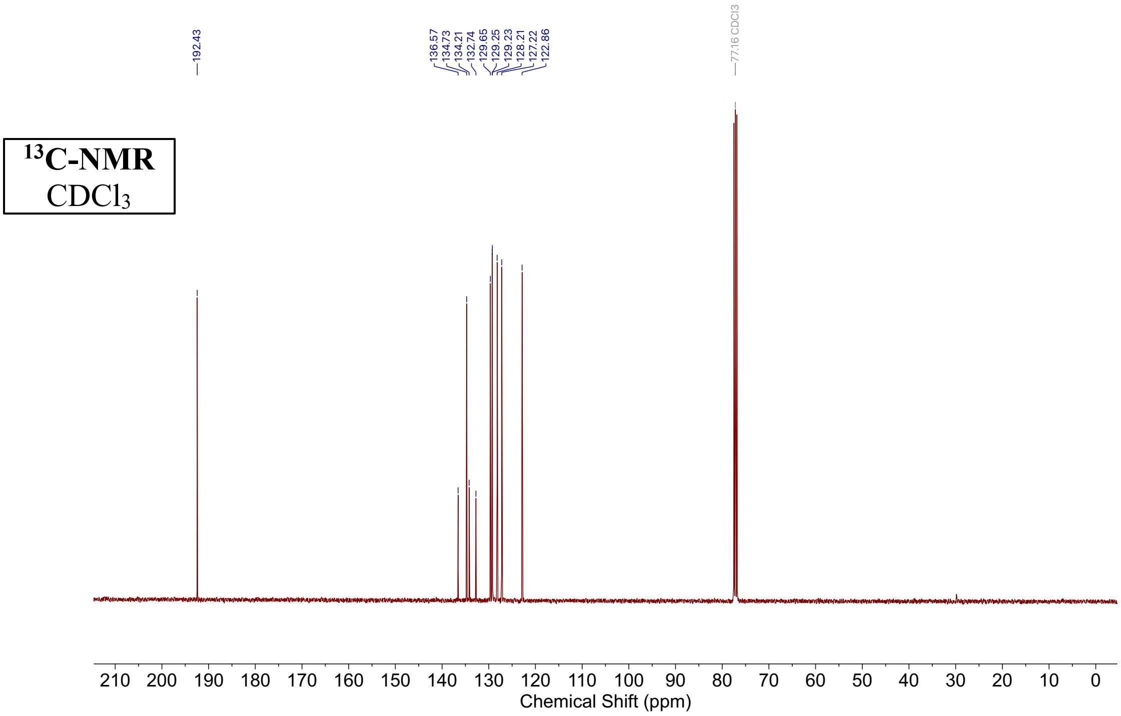

cb-e6-013

EMORY UNIVERSITY | X-ray Crystallography Center

R_1=8.11%

Submitted by: **Carter Brzezinski, Biegasiewicz Lab**

Data collected by: **John Bacsa**

Crystal Data and Experimental

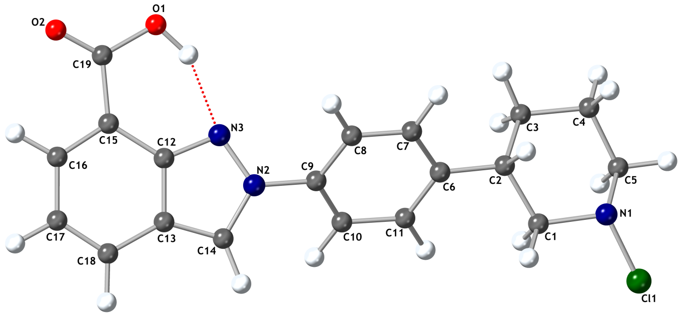

Experimental. Single colorless plate-shaped crystals of **cb-e6-013** were chosen from the sample as supplied. A suitable crystal with dimensions 0.41 × 0.10 × 0.03 mm³ was selected and mounted on a loop with paratone on a XtaLAB Synergy-S diffractometer. The crystal was kept at a constant *T* = 100.0(1) K during data collection. The structure was solved with the ShelXT (Sheldrick, 2015) solution program and Olex2 1.5-alpha (Dolomanov et al., 2009). The structure was refined with **ShelXL** 2018/3 (Sheldrick, 2015) using full matrix least squares minimisation on $\boldsymbol{F^2}$.

Crystal Data. $C_{19}H_{18}ClN_3O_2$, M_r = 355.81, triclinic, *P*1 (No. 1), a = 8.3963(3) Å, b = 14.9579(6) Å, c = 15.7502(7) Å, α = 116.071(4)°, β = 105.166(3)°, γ = 91.545(3)°, *V* = 1691.78(13) Å³, *T* = 100.00(10) K, *Z* = 4, *Z'* = 4, μ(Cu K$_\alpha$) = 2.148, 24708 reflections measured, 9191 unique (R_{int} = 0.0815) which were used in all calculations. The final wR_2 was 0.2230 (all data) and R_1 was 0.0811 (I≥2 σ(I)).

**Compound**	**cb-e6-013**
Formula	$C_{19}H_{18}ClN_3O_2$
$D_{calc.}$/ g cm^{-3}	1.397
μ/mm^{-1}	2.148
Formula Weight	355.81
Colour	colourless
Shape	plate-shaped
Size/mm³	0.41×0.10×0.03
T/K	100.00(10)
Crystal System	triclinic
Flack Parameter	-0.03(3)
Hooft Parameter	-0.04(2)
Space Group	*P*1
a/Å	8.3963(3)
b/Å	14.9579(6)
c/Å	15.7502(7)
α/°	116.071(4)
β/°	105.166(3)
γ/°	91.545(3)
V/Å³	1691.78(13)
Z	4
Z'	4
Wavelength/Å	1.54184
Radiation type	Cu K$_\alpha$
Θ_{min}/°	3.280
Θ_{max}/°	77.368
Measured Refl's.	24708
Indep't Refl's	9191
Refl's I≥2 σ(I)	7042
R_{int}	0.0815
Parameters	921
Restraints	922
Largest Peak	1.705
Deepest Hole	-0.702
GooF	1.026
wR_2 (all data)	0.2230
wR_2	0.2016
R_1 (all data)	0.1069
R_1	0.0811

Structure Quality Indicators

**Reflections:**	d min (Cu\a) 2θ=154.7° 0.79	I/σ(I) CIF 10.7	Rint CIF 8.15%	Full 135.4° 95% to 154.7° 99.7	
**Refinement:**	Shift CIF 0.000	Max Peak CIF 1.7	Min Peak CIF -0.7	GooF CIF 1.026	Flack CIF -.03(3)

A colourless plate-shaped-shaped crystal with dimensions 0.41 × 0.10 × 0.03 mm^3 was mounted on a loop with paratone. Data were collected using a XtaLAB Synergy, Dualflex, HyPix diffractometer operating at T = 100.0(1) K.

Data were measured using ω scans using Cu K$_\alpha$ radiation. The diffraction pattern was indexed and the total number of runs and images was based on the strategy calculation from the program CrysAlisPro system (CCD 44.57a 64-bit (release 20-06-2024)). The maximum resolution that was achieved was Θ = 77.368° (0.83 Å). The unit cell was refined using CrysAlisPro 1.171.44.57a (Rigaku OD, 2024) on 5911 reflections, 24% of the observed reflections.

The crystal has no rotational symmetry, and because the structure is chiral, the space group is limited to P1.

Data reduction, scaling and absorption corrections were performed using CrysAlisPro 1.171.44.57a (Rigaku OD, 2024). The final completeness is 99.70 % out to 77.368° in Θ. A numerical absorption correction based on gaussian integration over a multifaceted crystal model was performed using CrysAlisPro 1.171.42.74a (Rigaku Oxford Diffraction, 2022). An empirical absorption correction using spherical harmonics, implemented in SCALE3 ABSPACK scaling algorithm was also applied. The absorption coefficient μ of this material is 2.148 mm^{-1} at this wavelength (λ = 1.54184Å) and the minimum and maximum transmissions are 0.357 and 1.000.

The structure was solved and the space group P1 (# 1) determined by the ShelXT (Sheldrick, 2015) structure solution program using dual methods and refined by full matrix least squares minimisation on $\boldsymbol{F^2}$ using version 2018/3 of **ShelXL** 2018/3 (Sheldrick, 2015). All non-hydrogen atoms were refined anisotropically. Hydrogen atom positions were calculated geometrically and refined using the riding model.

The unit cell contains four whole molecules (Z'= 4) which includes a disordered molecule. The moiety formula is $C_{19}H_{18}ClN_3O_2$

The Flack parameter was refined to -0.03(3). Determination of absolute structure using Bayesian statistics on Bijvoet differences using the Olex2 results in -0.04(2). The chiral atom in this structure is C2(S), The Flack parameter is a crucial parameter for determining the chirality of the crystal under study. Ideally, its value should be close to 0. A value of 1 indicates that the stereochemistry is incorrect and the model should be inverted. A value of 0.5 signifies that the crystal is a racemic mixture of both enantiomers.

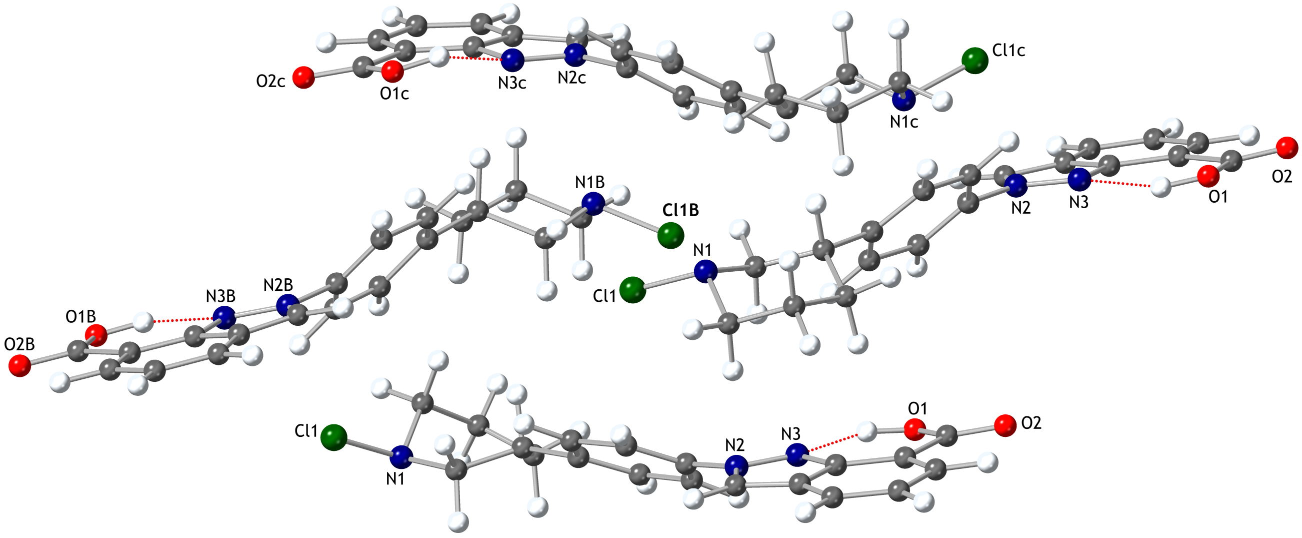

Figure S12: The unit cell (and the asymmetric unit) contains four chemically identical molecules.

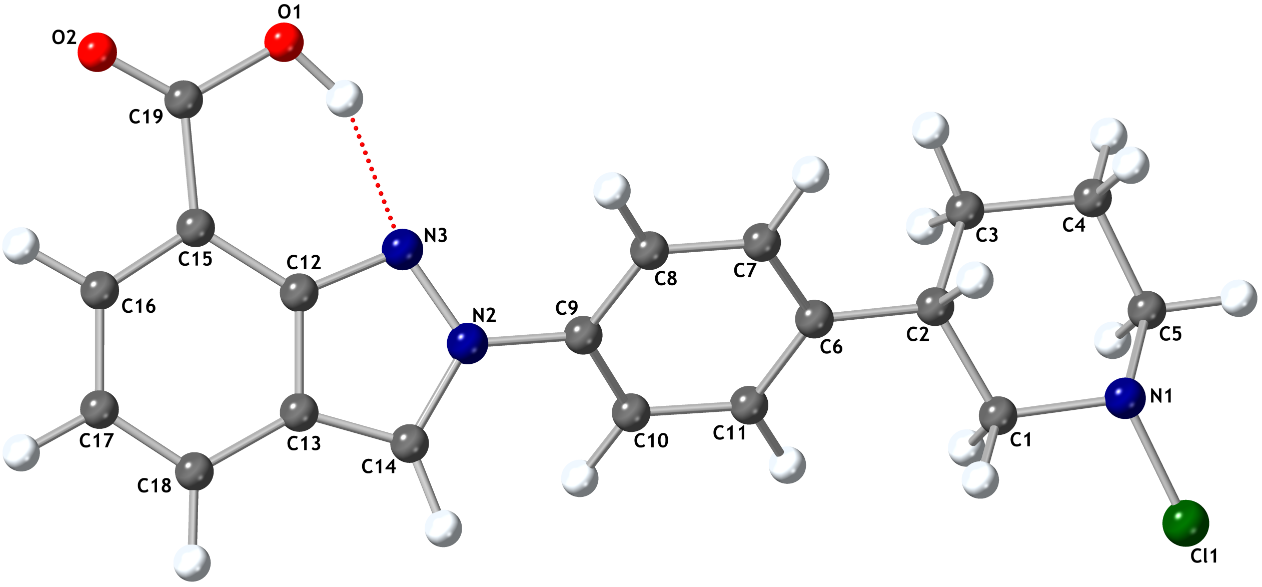

Figure S13: Molecular drawing of one of the four crystallographically independent molecules in the asymmetric unit the structure with the C, Cl, O, N and H atoms shown as spheres. There is one chiral atom in this molecule, C2(S),

Data Plots: Diffraction Data

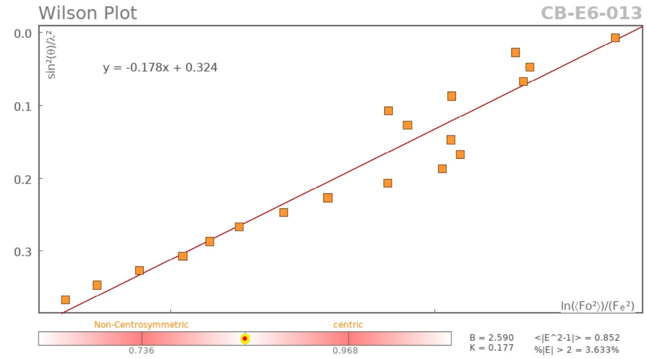

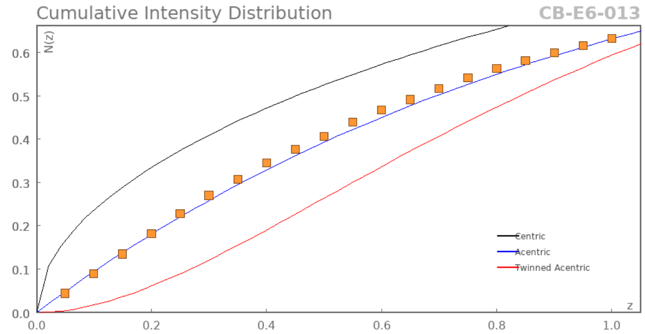

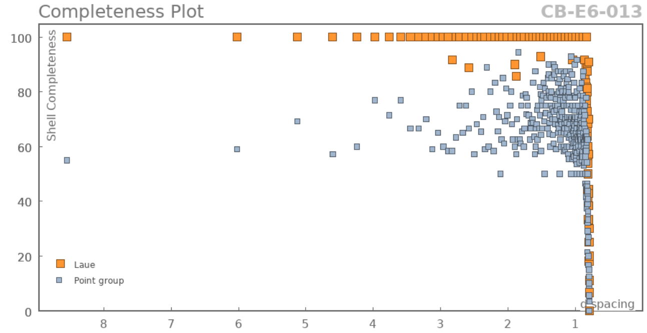

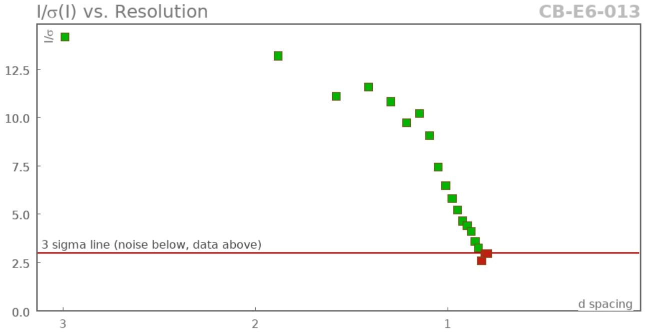

Data Plots: Refinement and Data

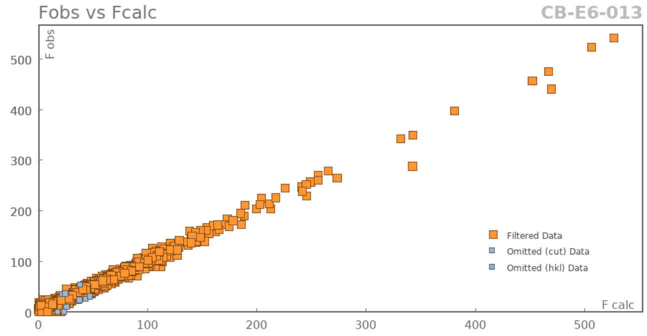

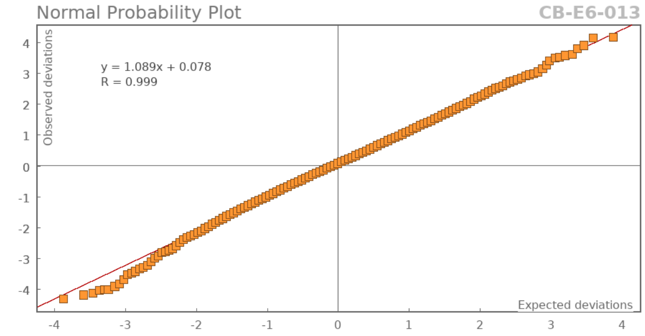

Images of the Crystal on the Diffractometer

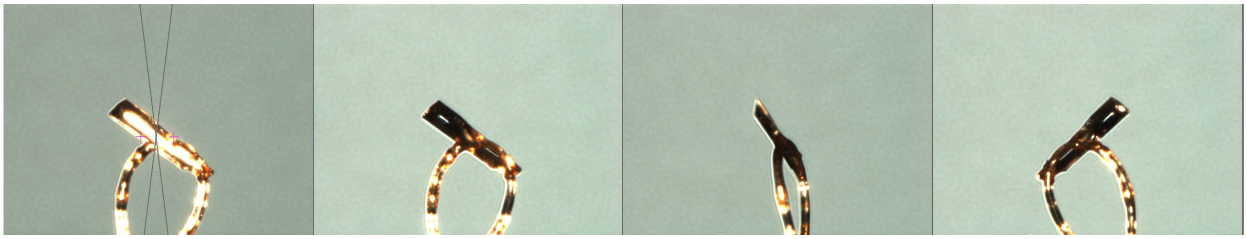

Table S1: Fractional Atomic Coordinates (×10[4]) and Equivalent Isotropic Displacement Parameters (Å[2]×10[3]) for **cb-e6-013**. U_{eq} is defined as 1/3 of the trace of the orthogonalised U_{ij}.

Atom	x	y	z	U_{eq}
Cl1B	8283(3)	118(2)	3295.6(19)	44.2(4)
Cl2	-788(3)	-4293(2)	-1392(2)	43.2(5)
O1	12283(8)	-3865(5)	-5087(5)	42.6(7)
O1B	10225(8)	4959(5)	12163(5)	42.6(7)
O2	13351(8)	-4710(5)	-6341(5)	45.0(17)
O2B	9154(8)	5840(5)	13407(4)	35.4(13)
O3	4143(7)	780(5)	-1516(4)	30.2(12)
O4	6002(8)	-591(5)	7403(5)	42.6(7)
O8	8346(8)	45(5)	8718(4)	33.0(12)
O10	6500(8)	1409(5)	-178(5)	42.6(7)
N1B	9414(9)	139(6)	4408(5)	35.3(14)
N2	15476(8)	-2542(5)	-2175(5)	22.6(13)
N2B	6980(8)	3374(5)	9266(5)	24.7(11)
N3	14482(9)	-3017(5)	-3148(5)	26.3(14)
N3B	7967(8)	3928(5)	10217(5)	23.7(11)
N9	6071(8)	2450(5)	1674(5)	23.3(11)
N11	6411(8)	-1467(5)	5536(5)	26.1(12)
N16	6018(8)	2992(5)	2625(5)	24.6(11)
N65	-929(9)	-3326(6)	-262(5)	34.2(15)
N69	6444(9)	-1899(5)	4580(5)	27.2(12)
C1B	8842(11)	905(6)	5212(6)	30.5(15)
C2B	9801(11)	943(7)	6207(6)	34.2(15)
C3B	11673(11)	1192(7)	6422(7)	36.8(17)
C4B	12200(12)	456(8)	5536(7)	40.5(18)
C5B	11190(11)	432(7)	4582(6)	35.2(16)
C6B	9089(10)	1630(6)	7019(6)	25.9(13)
C7B	9911(10)	2582(6)	7772(6)	28.7(14)
C8B	9229(10)	3161(6)	8513(6)	26.9(13)
C9B	7694(10)	2789(6)	8498(6)	24.4(12)
C10B	6818(10)	1854(6)	7753(6)	27.1(14)
C11B	7518(10)	1297(6)	7018(6)	29.1(15)
C12	15590(10)	-3384(6)	-3654(6)	26.3(16)
C12B	6899(10)	4348(5)	10730(6)	23.5(12)
C13	17256(10)	-3146(6)	-3018(6)	25.2(15)
C13B	5229(10)	4058(6)	10085(6)	29.5(14)
C14	17133(10)	-2590(6)	-2060(6)	24.2(15)
C14B	5350(11)	3419(6)	9154(6)	33.5(16)
C15	15253(10)	-3935(6)	-4707(6)	29.4(17)
C15B	7240(10)	4982(6)	11771(6)	26.0(12)
C16	16596(10)	-4215(6)	-5041(6)	27.1(16)
C16B	5909(11)	5313(6)	12098(6)	28.9(14)
C17	18248(11)	-3986(6)	-4411(6)	31.0(17)
C17B	4228(11)	5054(7)	11453(7)	35.6(17)
C18	18614(10)	-3457(6)	-3395(6)	27.8(16)
C18B	3884(12)	4416(7)	10457(7)	41(2)
C20	4837(10)	1284(6)	-613(6)	25.0(12)
C21	4490(10)	2346(6)	1119(5)	22.4(12)
C23	-1231(10)	-2656(7)	1685(7)	38.3(19)
C25	7669(10)	-452(6)	7807(6)	26.4(12)
C29	11389(11)	-1311(6)	6883(7)	31.6(14)
C31	10758(11)	-1740(6)	5871(6)	29.5(14)
C32	457(11)	-3342(7)	537(7)	36.4(17)
C33	2017(10)	-2367(6)	2329(6)	27.7(13)
C37	9029(10)	-1774(6)	5465(6)	26.2(13)
C38	1739(10)	2791(6)	1264(6)	29.4(14)

Atom	x	y	z	U_{eq}
C39	-2537(11)	-3583(7)	-155(7)	40.7(19)
C42	13548(11)	-4195(7)	-5441(6)	35(2)
C42B	8943(11)	5285(6)	12504(6)	29.8(14)
C43	5062(10)	-1984(6)	2986(6)	30.5(14)
C46	437(10)	-2496(6)	1515(6)	30.8(15)
C55	3815(10)	1817(5)	50(5)	21.4(12)
C56	1113(10)	2295(6)	260(6)	29.4(14)
C57	4503(10)	3230(6)	2679(6)	25.7(13)
C60	3455(10)	2821(6)	1716(6)	26.3(13)
C61	2141(10)	1809(6)	-345(6)	25.1(13)
C62	1973(10)	-2443(6)	3170(6)	32.2(15)
C63	7989(10)	-1391(5)	6068(6)	24.0(12)
C64	-2662(11)	-2765(8)	820(8)	45(2)
C65	10356(10)	-902(6)	7504(6)	28.0(14)
C66	8682(10)	-926(5)	7123(6)	24.8(12)
C67	3583(10)	-2133(6)	2247(6)	30.2(14)
C68	4965(10)	-2062(6)	3818(6)	26.5(12)
C70	3431(10)	-2300(7)	3903(6)	32.5(15)
C76	7989(10)	-2110(6)	4495(6)	28.6(14)
Cl1_1	14261(3)	1196(2)	3831.0(19)	44.2(4)
N1_1	12810(8)	459(4)	2665(4)	32.3(13)
C1_1	13752(7)	140(5)	1937(4)	28.7(14)
C2_1	12530(7)	-543(4)	913(4)	28.2(13)
C3_1	11126(8)	-3(6)	631(4)	35.3(17)
C4_1	10249(9)	420(6)	1453(6)	37.3(17)
C5_1	11568(9)	1083(6)	2466(5)	35.6(15)
C6_1	13359(8)	-1051(5)	114(5)	23.2(13)
C7_1	12538(9)	-1965(5)	-696(5)	29.8(15)
C8_1	13221(9)	-2460(5)	-1460(5)	29.7(15)
C9_1	14756(9)	-2033(5)	-1408(5)	22.2(13)
C10_1	15587(9)	-1112(5)	-618(5)	24.8(14)
C11_1	14897(9)	-631(5)	152(5)	28.0(15)
Cl1_2	13274(7)	5076(4)	8656(3)	62.4(15)
N1_2	13602(9)	4864(6)	7538(5)	41.1(15)
C1_2	11960(9)	4416(8)	6768(5)	38.9(15)
C2_2	12156(8)	4233(7)	5773(5)	38.4(14)
C3_2	13479(11)	3573(9)	5506(5)	43.3(16)
C4_2	15153(9)	4008(10)	6370(7)	44.2(15)
C5_2	14815(10)	4153(9)	7337(6)	43.2(15)
C6_2	10511(9)	3878(9)	4945(6)	30.8(18)
C7_2	10533(12)	3715(13)	4011(7)	31(2)
C8_2	9058(15)	3430(20)	3235(8)	29.6(17)
C9_2	7555(12)	3220(30)	3381(10)	23.5(15)
C10_2	7501(11)	3360(20)	4301(10)	31.6(18)
C11_2	8993(11)	3654(13)	5074(7)	28.2(18)
Cl1_3	16287(5)	3185(4)	6338(3)	44.2(4)
N1_3	14478(9)	3550(6)	6649(6)	44.2(15)
C1_3	13266(8)	3494(8)	5749(5)	43.3(16)
C2_3	11641(7)	3770(6)	5979(5)	38.4(14)
C3_3	11915(11)	4815(7)	6858(7)	38.9(15)
C4_3	13307(12)	4886(9)	7773(6)	41.1(15)
C5_3	14900(10)	4594(7)	7471(6)	43.2(15)
C6_3	10193(9)	3593(9)	5086(6)	30.8(18)
C7_3	8579(9)	3480(15)	5139(8)	28.2(18)
C8_3	7195(10)	3290(20)	4339(11)	31.6(18)
C9_3	7430(13)	3250(30)	3481(11)	23.5(15)
C10_3	9026(15)	3380(20)	3414(10)	29.6(17)
C11_3	10405(11)	3534(15)	4214(8)	31(2)

Table S2: Anisotropic Displacement Parameters (×10[4]) for **cb-e6-013**. The anisotropic displacement factor exponent takes the form: $-2\pi^2[h^2a^{*2} \times U_{11} + ... + 2hka^* \times b^* \times U_{12}]$

Atom	U_{11}	U_{22}	U_{33}	U_{23}	U_{13}	U_{12}
Cl1B	38.4(7)	52.0(8)	27.7(6)	6.8(6)	9.8(5)	-1.6(6)
Cl2	41.6(13)	43.6(11)	37.9(11)	19.0(9)	1.7(9)	3.4(9)
O1	28.1(9)	52.8(17)	32.7(12)	8.9(12)	6.3(8)	9.0(9)
O1B	28.1(9)	52.8(17)	32.7(12)	8.9(12)	6.3(8)	9.0(9)
O2	27(3)	55(4)	22(3)	-4(3)	-1(3)	17(3)
O2B	30(3)	35(3)	26.6(16)	5.3(16)	3.0(14)	9(2)
O3	24(2)	37(3)	21.6(14)	6.2(14)	8.3(10)	7(2)
O4	28.1(9)	52.8(17)	32.7(12)	8.9(12)	6.3(8)	9.0(9)
O8	26(3)	40(3)	23.4(14)	5.8(15)	7.9(11)	7(2)
O10	28.1(9)	52.8(17)	32.7(12)	8.9(12)	6.3(8)	9.0(9)
N1B	33(2)	36(3)	28(2)	3.4(18)	15.3(18)	2.5(19)
N2	16(3)	27(3)	16(3)	5(2)	-1(2)	0(2)
N2B	19.6(19)	27(2)	20.3(16)	7.2(14)	1.1(13)	2.9(16)
N3	24(3)	27(3)	18(3)	3(2)	4(3)	3(3)
N3B	18.0(19)	26(2)	20.2(16)	7.1(15)	1.8(14)	2.0(16)
N9	20.3(19)	26(3)	17.2(14)	5.9(15)	3.3(12)	4.1(17)
N11	20.5(19)	28(3)	24.2(16)	5.4(16)	9.5(12)	4.0(17)
N16	22.2(17)	27(3)	17.7(14)	5.6(15)	4.4(11)	2.9(16)
N65	34(3)	40(4)	29(3)	16(3)	12(3)	8(3)
N69	23.4(17)	27(3)	24.5(16)	4.9(16)	10.3(11)	3.0(16)
C1B	30(3)	33(3)	23.9(18)	7.2(19)	13(2)	5(3)
C2B	35(3)	39(3)	25.6(18)	10.2(18)	13.5(19)	9(2)
C3B	35(3)	47(4)	31(3)	18(3)	15(2)	11(2)
C4B	36(3)	52(4)	33(3)	16(2)	16.5(18)	11(3)
C5B	32(2)	40(4)	31(3)	12(3)	15.2(18)	5(2)
C6B	26(2)	30(2)	20.5(19)	10.8(17)	7.7(18)	5.0(17)
C7B	30(3)	31(2)	22(2)	10.3(18)	7.4(19)	3.6(19)
C8B	26(2)	31(3)	19(2)	9.2(19)	3.5(19)	3.0(18)
C9B	25(2)	25(2)	21.7(18)	10.9(16)	4.3(17)	6.5(17)
C10B	28(3)	28(2)	23(2)	8.1(17)	9.8(19)	2.6(19)
C11B	28(2)	31(3)	23(2)	7(2)	10(2)	3(2)
C12	20(4)	27(4)	26(4)	9(3)	2(3)	7(3)
C12B	19(2)	22(3)	23.8(15)	6.9(15)	4.4(13)	0.4(16)
C13	23(4)	25(3)	23(4)	7(3)	4(3)	7(3)
C13B	20(2)	26(3)	28(2)	3.8(19)	2.2(13)	1.7(17)
C14	17(3)	25(3)	21(4)	7(3)	-1(3)	3(3)
C14B	19.7(19)	35(3)	28(2)	2(2)	1.4(13)	3.6(17)
C15	20(4)	29(4)	22(4)	0(3)	1(3)	5(3)
C15B	23.4(16)	24(3)	23.9(15)	6.8(15)	5.2(12)	0.8(16)
C16	27(4)	21(3)	22(4)	-1(3)	9(3)	5(3)
C16B	24.4(18)	26(3)	29(3)	7(2)	7.7(17)	0.8(18)
C17	27(4)	32(4)	25(4)	7(3)	5(3)	6(3)
C17B	25.2(19)	33(4)	33(2)	4(2)	5.9(18)	3(2)
C18	21(4)	25(4)	34(4)	12(3)	5(3)	5(3)
C18B	23(2)	44(4)	34(2)	2(2)	4.9(19)	5(2)
C20	26.9(10)	24(3)	21.5(14)	8.0(15)	8.3(9)	5.3(12)
C21	19.8(19)	27(3)	17.7(14)	8.5(15)	3.9(11)	3.3(17)
C23	25(4)	47(5)	47(5)	26(4)	9(3)	2(4)
C25	27.1(10)	25(3)	23.1(14)	7.5(15)	8.1(9)	6.6(12)
C29	27(2)	30(3)	33(2)	8(2)	11.9(15)	3(2)
C31	24.5(18)	27(3)	33(2)	8(2)	12.1(15)	6.7(19)
C32	27(4)	42(4)	36(4)	17(3)	5(3)	4(3)
C33	23(2)	29(3)	27(2)	9(2)	8.9(16)	2.6(19)

Atom	U_{11}	U_{22}	U_{33}	U_{23}	U_{13}	U_{12}
C37	24.2(18)	24(3)	28.8(18)	7.9(18)	12.7(14)	5.8(18)
C38	21.8(18)	34(4)	28(2)	13(2)	5.1(15)	4.0(19)
C39	26(4)	49(5)	49(4)	31(4)	1(4)	-2(4)
C42	28(4)	39(4)	20(4)	-1(3)	6(3)	18(4)
C42B	25.1(14)	26(3)	26.6(16)	5.5(16)	3.0(12)	1.8(14)
C43	22(2)	38(4)	30.6(19)	14(2)	10.7(16)	5(2)
C46	24(2)	32(4)	34(2)	16(3)	5(2)	-3(2)
C55	20.8(17)	24(3)	17.7(14)	8.8(15)	3.6(12)	4.8(16)
C56	21(2)	35(3)	28(2)	13(2)	5.2(15)	4(2)
C57	22.4(17)	29(3)	19.8(18)	6.7(19)	5.1(13)	3.5(17)
C60	21.6(18)	32(3)	19.8(17)	7.2(17)	5.5(13)	3.9(18)
C61	20.8(17)	31(3)	25(2)	16(2)	3.7(13)	5.1(17)
C62	25(2)	38(4)	28(2)	11(2)	6.7(17)	-3(3)
C63	20.3(19)	21(3)	25.3(14)	4.9(15)	8.9(12)	2.6(17)
C64	27(4)	64(6)	57(4)	39(4)	13(4)	14(4)
C65	23.7(17)	26(3)	28(2)	7(2)	8.1(14)	2.2(18)
C66	23.5(17)	20(3)	25.3(14)	5.0(15)	8.1(12)	0.8(17)
C67	23(2)	36(4)	32(2)	15(3)	9.7(15)	4.0(19)
C68	24.1(17)	23(3)	26.0(17)	6(2)	9.0(13)	1.0(17)
C70	24.3(18)	41(4)	26(3)	10(3)	7.8(15)	-1.9(18)
C76	24.6(18)	30(3)	28.9(19)	9(2)	12.7(14)	5.0(18)
Cl1_1	38.4(7)	52.0(8)	27.7(6)	6.8(6)	9.8(5)	-1.6(6)
N1_1	35(2)	31(2)	28(2)	11(2)	12(2)	4(2)
C1_1	29(3)	31(3)	24.3(18)	12.0(18)	7.0(19)	6(2)
C2_1	27(2)	33(3)	23.8(18)	12.2(17)	8.0(18)	5(2)
C3_1	34(3)	46(4)	31(3)	20(3)	13(2)	15(3)
C4_1	41(3)	43(4)	36(3)	21(2)	19(2)	15(3)
C5_1	40(3)	43(3)	34(3)	21(2)	21(2)	12(2)
C6_1	20(2)	26(2)	19.5(18)	9.4(17)	2.5(17)	0.5(17)
C7_1	24(3)	30(2)	24(2)	5.7(18)	3.9(19)	-3(2)
C8_1	25(2)	29(3)	24(2)	5(2)	4(2)	-3(2)
C9_1	21(2)	21(2)	18(2)	6.3(19)	1.1(18)	1.7(18)
C10_1	23(3)	24(2)	20(2)	3.8(19)	5.3(19)	-1.5(19)
C11_1	24(2)	27(3)	21(2)	2(2)	7(2)	-3.7(19)
Cl1_2	48(3)	55(2)	87(3)	38(2)	14.6(19)	13(2)
N1_2	40(2)	44(2)	37.7(18)	17.1(14)	11.0(13)	4.6(15)
C1_2	39(2)	40(2)	34.4(17)	13.7(15)	12.7(15)	6.3(18)
C2_2	37.5(18)	40(2)	33.9(17)	13.6(15)	11.9(13)	5.5(15)
C3_2	42.0(18)	47(3)	41(2)	17.7(17)	17.1(15)	10.5(17)
C4_2	43.6(19)	48(2)	42(2)	20.5(14)	15.4(12)	5.5(12)
C5_2	42(2)	47(2)	42(2)	20.9(15)	14.1(15)	7.6(17)
C6_2	34(2)	28(5)	28.9(19)	9(2)	15.7(16)	8(2)
C7_2	23(2)	38(5)	28(2)	10(2)	10.7(17)	4(3)
C8_2	23.1(19)	34(4)	28(3)	9(3)	10.7(16)	4(2)
C9_2	20.7(19)	28(3)	16.6(18)	5(2)	5.8(16)	3(2)
C10_2	33(2)	39(4)	18.9(18)	9(2)	9.5(18)	4(3)
C11_2	32(2)	31(5)	17(2)	5(3)	10.4(17)	6(2)
Cl1_3	38.4(7)	52.0(8)	27.7(6)	6.8(6)	9.8(5)	-1.6(6)
N1_3	43.6(19)	48(2)	42(2)	20.5(14)	15.4(12)	5.5(12)
C1_3	42.0(18)	47(3)	41(2)	17.7(17)	17.1(15)	10.5(17)
C2_3	37.5(18)	40(2)	33.9(17)	13.6(15)	11.9(13)	5.5(15)
C3_3	39(2)	40(2)	34.4(17)	13.7(15)	12.7(15)	6.3(18)
C4_3	40(2)	44(2)	37.7(18)	17.1(14)	11.0(13)	4.6(15)
C5_3	42(2)	47(2)	42(2)	20.9(15)	14.1(15)	7.6(17)
C6_3	34(2)	28(5)	28.9(19)	9(2)	15.7(16)	8(2)
C7_3	32(2)	31(5)	17(2)	5(3)	10.4(17)	6(2)
C8_3	33(2)	39(4)	18.9(18)	9(2)	9.5(18)	4(3)
C9_3	20.7(19)	28(3)	16.6(18)	5(2)	5.8(16)	3(2)

Atom	U_{11}	U_{22}	U_{33}	U_{23}	U_{13}	U_{12}
C10_3	23.1(19)	34(4)	28(3)	9(3)	10.7(16)	4(2)
C11_3	23(2)	38(5)	28(2)	10(2)	10.7(17)	4(3)

Table S3: Bond Lengths in Å for **cb-e6-013**.

Atom	Atom	Length/Å
Cl1B	N1B	1.747(8)
Cl2	N65	1.770(7)
O1	C42	1.334(10)
O1B	C42B	1.336(11)
O2	C42	1.241(10)
O2B	C42B	1.251(10)
O3	C20	1.238(9)
O4	C25	1.345(10)
O8	C25	1.245(10)
O10	C20	1.351(10)
N1B	C1B	1.490(10)
N1B	C5B	1.461(11)
N2	N3	1.375(9)
N2	C14	1.364(10)
N2	C9_1	1.416(8)
N2B	N3B	1.356(9)
N2B	C9B	1.437(10)
N2B	C14B	1.340(11)
N3	C12	1.353(10)
N3B	C12B	1.347(10)
N9	N16	1.367(8)
N9	C21	1.346(10)
N11	N69	1.361(9)
N11	C63	1.341(10)
N16	C57	1.344(10)
N16	C9_2	1.419(10)
N16	C9_3	1.434(10)
N65	C32	1.484(11)
N65	C39	1.465(11)
N69	C68	1.410(11)
N69	C76	1.370(10)
C1B	C2B	1.538(12)
C2B	C3B	1.517(12)
C2B	C6B	1.526(10)
C3B	C4B	1.534(12)
C4B	C5B	1.503(13)
C6B	C7B	1.394(11)
C6B	C11B	1.397(11)
C7B	C8B	1.389(11)
C8B	C9B	1.379(11)
C9B	C10B	1.387(10)
C10B	C11B	1.384(10)
C12	C13	1.419(11)
C12	C15	1.432(11)
C12B	C13B	1.422(11)
C12B	C15B	1.429(11)
C13	C14	1.400(11)
C13	C18	1.418(11)
C13B	C14B	1.387(12)
C13B	C18B	1.418(12)
C15	C16	1.365(11)
C15	C42	1.498(11)
C15B	C16B	1.366(12)
C15B	C42B	1.491(12)
C16	C17	1.407(12)
C16B	C17B	1.431(12)
C17	C18	1.377(11)
C17B	C18B	1.373(12)
C20	C55	1.495(10)
C21	C55	1.445(10)
C21	C60	1.421(11)
C23	C46	1.525(11)
C23	C64	1.505(13)
C25	C66	1.492(10)
C29	C31	1.369(12)
C29	C65	1.425(11)
C31	C37	1.414(12)
C32	C46	1.509(12)
C33	C46	1.523(11)
C33	C62	1.389(11)
C33	C67	1.403(11)
C37	C63	1.406(10)
C37	C76	1.397(12)
C38	C56	1.357(12)
C38	C60	1.420(11)
C39	C64	1.518(15)
C43	C67	1.396(11)
C43	C68	1.387(11)
C55	C61	1.377(11)
C56	C61	1.427(11)
C57	C60	1.380(11)
C62	C70	1.381(12)
C63	C66	1.428(11)
C65	C66	1.369(11)
C68	C70	1.382(11)
Cl1_1	N1_1	1.746(6)
N1_1	C1_1	1.478(8)
N1_1	C5_1	1.481(9)
C1_1	C2_1	1.526(3)
C2_1	C3_1	1.526(3)
C2_1	C6_1	1.513(8)
C3_1	C4_1	1.557(8)
C4_1	C5_1	1.543(10)
C6_1	C7_1	1.389(8)
C6_1	C11_1	1.396(9)
C7_1	C8_1	1.388(9)
C8_1	C9_1	1.389(9)
C9_1	C10_1	1.386(8)
C10_1	C11_1	1.395(9)
Cl1_2	N1_2	1.746(6)

Atom	Atom	Length/Å
N1_2	C1_2	1.477(8)
N1_2	C5_2	1.483(9)
C1_2	C2_2	1.524(3)
C2_2	C3_2	1.525(3)
C2_2	C6_2	1.516(8)
C3_2	C4_2	1.557(8)
C4_2	C5_2	1.545(10)
C6_2	C7_2	1.388(9)
C6_2	C11_2	1.397(9)
C7_2	C8_2	1.388(9)
C8_2	C9_2	1.389(9)
C9_2	C10_2	1.387(8)
C10_2	C11_2	1.395(9)
Cl1_3	N1_3	1.745(6)

Atom	Atom	Length/Å
N1_3	C1_3	1.478(8)
N1_3	C5_3	1.482(9)
C1_3	C2_3	1.524(2)
C2_3	C3_3	1.525(2)
C2_3	C6_3	1.512(8)
C3_3	C4_3	1.560(8)
C4_3	C5_3	1.546(10)
C6_3	C7_3	1.390(9)
C6_3	C11_3	1.396(9)
C7_3	C8_3	1.387(9)
C8_3	C9_3	1.389(9)
C9_3	C10_3	1.386(8)
C10_3	C11_3	1.395(9)

Table S4: Bond Angles in ° for **cb-e6-013**.

Atom	Atom	Atom	Angle/°
C1B	N1B	Cl1B	107.3(6)
C5B	N1B	Cl1B	108.4(5)
C5B	N1B	C1B	110.1(6)
N3	N2	C9_1	119.9(6)
C14	N2	N3	113.8(6)
C14	N2	C9_1	126.2(6)
N3B	N2B	C9B	120.5(6)
C14B	N2B	N3B	113.3(6)
C14B	N2B	C9B	126.2(7)
C12	N3	N2	103.1(6)
C12B	N3B	N2B	104.7(6)
C21	N9	N16	103.3(6)
C63	N11	N69	104.2(6)
N9	N16	C9_2	115.5(10)
N9	N16	C9_3	123.1(10)
C57	N16	N9	113.6(6)
C57	N16	C9_2	130.9(10)
C57	N16	C9_3	123.3(10)
C32	N65	Cl2	106.3(5)
C39	N65	Cl2	107.6(6)
C39	N65	C32	109.6(7)
N11	N69	C68	118.8(6)
N11	N69	C76	113.0(7)
C76	N69	C68	128.1(7)
N1B	C1B	C2B	109.1(7)
C3B	C2B	C1B	110.7(7)
C3B	C2B	C6B	115.5(7)
C6B	C2B	C1B	109.8(7)
C2B	C3B	C4B	109.6(7)
C5B	C4B	C3B	111.7(8)
N1B	C5B	C4B	109.7(7)
C7B	C6B	C2B	124.0(7)
C7B	C6B	C11B	117.2(7)
C11B	C6B	C2B	118.7(7)
C8B	C7B	C6B	121.5(7)
C9B	C8B	C7B	119.2(7)
C8B	C9B	N2B	119.9(7)
C8B	C9B	C10B	121.3(7)
C10B	C9B	N2B	118.9(7)

Atom	Atom	Atom	Angle/°
C11B	C10B	C9B	118.4(7)
C10B	C11B	C6B	122.3(7)
N3	C12	C13	112.5(7)
N3	C12	C15	127.6(7)
C13	C12	C15	119.9(7)
N3B	C12B	C13B	110.4(7)
N3B	C12B	C15B	129.2(7)
C13B	C12B	C15B	120.3(7)
C14	C13	C12	104.7(7)
C14	C13	C18	133.6(7)
C18	C13	C12	121.7(7)
C14B	C13B	C12B	105.0(7)
C14B	C13B	C18B	134.4(8)
C18B	C13B	C12B	120.7(7)
N2	C14	C13	105.9(6)
N2B	C14B	C13B	106.6(7)
C12	C15	C42	123.9(7)
C16	C15	C12	116.6(7)
C16	C15	C42	119.5(7)
C12B	C15B	C42B	123.8(7)
C16B	C15B	C12B	117.1(7)
C16B	C15B	C42B	119.2(7)
C15	C16	C17	123.6(7)
C15B	C16B	C17B	123.2(8)
C18	C17	C16	121.3(8)
C18B	C17B	C16B	120.0(8)
C17	C18	C13	116.9(7)
C17B	C18B	C13B	118.7(8)
O3	C20	O10	123.7(7)
O3	C20	C55	119.4(7)
O10	C20	C55	116.9(6)
N9	C21	C55	127.5(7)
N9	C21	C60	111.6(6)
C60	C21	C55	120.8(7)
C64	C23	C46	110.5(7)
O4	C25	C66	117.6(7)
O8	C25	O4	121.4(7)
O8	C25	C66	121.0(7)
C31	C29	C65	121.5(8)

Atom	Atom	Atom	Angle/°
C29	C31	C37	117.3(7)
N65	C32	C46	108.2(7)
C62	C33	C46	122.5(7)
C62	C33	C67	118.2(7)
C67	C33	C46	119.3(7)
C63	C37	C31	121.8(7)
C76	C37	C31	133.0(7)
C76	C37	C63	105.1(7)
C56	C38	C60	118.8(8)
N65	C39	C64	108.1(7)
O1	C42	C15	117.6(7)
O2	C42	O1	122.4(8)
O2	C42	C15	120.0(7)
O1B	C42B	C15B	118.0(7)
O2B	C42B	O1B	121.5(8)
O2B	C42B	C15B	120.5(8)
C68	C43	C67	118.8(7)
C32	C46	C23	109.3(7)
C32	C46	C33	109.8(7)
C33	C46	C23	117.0(7)
C21	C55	C20	123.0(7)
C61	C55	C20	120.5(7)
C61	C55	C21	116.4(7)
C38	C56	C61	121.4(8)
N16	C57	C60	106.7(7)
C38	C60	C21	120.0(7)
C57	C60	C21	104.7(7)
C57	C60	C38	135.3(7)
C55	C61	C56	122.4(7)
C70	C62	C33	120.9(7)
N11	C63	C37	112.1(7)
N11	C63	C66	127.8(7)
C37	C63	C66	120.0(7)
C23	C64	C39	111.5(8)
C66	C65	C29	122.0(8)
C63	C66	C25	122.7(7)
C65	C66	C25	119.8(7)
C65	C66	C63	117.4(7)
C43	C67	C33	121.3(7)
C43	C68	N69	119.7(7)
C70	C68	N69	119.9(7)
C70	C68	C43	120.4(8)
C62	C70	C68	120.4(8)
N69	C76	C37	105.5(7)
C1_1	N1_1	Cl1_1	106.9(4)
C1_1	N1_1	C5_1	111.0(5)
C5_1	N1_1	Cl1_1	108.4(4)
N1_1	C1_1	C2_1	108.3(5)
C1_1	C2_1	C3_1	111.3(4)
C6_1	C2_1	C1_1	114.1(5)
C6_1	C2_1	C3_1	113.6(5)
C2_1	C3_1	C4_1	110.2(4)
C5_1	C4_1	C3_1	109.5(5)
N1_1	C5_1	C4_1	108.4(5)
C7_1	C6_1	C2_1	118.4(5)
C7_1	C6_1	C11_1	118.8(5)
C11_1	C6_1	C2_1	122.7(5)
C8_1	C7_1	C6_1	121.1(6)
C7_1	C8_1	C9_1	119.3(6)
C8_1	C9_1	N2	119.6(6)
C10_1	C9_1	N2	119.5(6)
C10_1	C9_1	C8_1	120.9(5)
C9_1	C10_1	C11_1	119.2(6)
C10_1	C11_1	C6_1	120.7(6)
C1_2	N1_2	Cl1_2	106.4(4)
C1_2	N1_2	C5_2	110.9(5)
C5_2	N1_2	Cl1_2	108.4(5)
N1_2	C1_2	C2_2	108.9(5)
C1_2	C2_2	C3_2	112.0(4)
C6_2	C2_2	C1_2	113.4(5)
C6_2	C2_2	C3_2	113.4(5)
C2_2	C3_2	C4_2	110.6(5)
C5_2	C4_2	C3_2	109.1(5)
N1_2	C5_2	C4_2	107.9(5)
C7_2	C6_2	C2_2	118.5(6)
C7_2	C6_2	C11_2	118.9(6)
C11_2	C6_2	C2_2	122.5(6)
C6_2	C7_2	C8_2	120.9(6)
C7_2	C8_2	C9_2	119.3(6)
C8_2	C9_2	N16	120.5(10)
C10_2	C9_2	N16	118.2(9)
C10_2	C9_2	C8_2	120.9(6)
C9_2	C10_2	C11_2	119.1(6)
C10_2	C11_2	C6_2	120.6(6)
C1_3	N1_3	Cl1_3	106.7(4)
C1_3	N1_3	C5_3	111.0(5)
C5_3	N1_3	Cl1_3	108.7(5)
N1_3	C1_3	C2_3	108.4(5)
C1_3	C2_3	C3_3	111.7(4)
C6_3	C2_3	C1_3	114.5(5)
C6_3	C2_3	C3_3	113.9(5)
C2_3	C3_3	C4_3	110.0(5)
C5_3	C4_3	C3_3	108.8(5)
N1_3	C5_3	C4_3	108.0(5)
C7_3	C6_3	C2_3	118.2(6)
C7_3	C6_3	C11_3	118.8(6)
C11_3	C6_3	C2_3	123.0(6)
C8_3	C7_3	C6_3	121.1(6)
C7_3	C8_3	C9_3	119.3(6)
C8_3	C9_3	N16	120.0(10)
C10_3	C9_3	N16	119.0(10)
C10_3	C9_3	C8_3	120.9(6)
C9_3	C10_3	C11_3	119.2(6)
C10_3	C11_3	C6_3	120.8(6)

Table S5: Torsion Angles in ° for **cb-e6-013**.

Atom	Atom	Atom	Atom	Angle/°
Cl1B	N1B	C1B	C2B	179.6(6)
Cl1B	N1B	C5B	C4B	-180.0(6)
Cl2	N65	C32	C46	177.4(5)
Cl2	N65	C39	C64	179.2(6)
O3	C20	C55	C21	174.3(7)
O3	C20	C55	C61	-4.6(11)
O4	C25	C66	C63	8.7(11)
O4	C25	C66	C65	-172.8(7)
O8	C25	C66	C63	-171.9(7)
O8	C25	C66	C65	6.6(11)
O10	C20	C55	C21	-7.4(10)
O10	C20	C55	C61	173.7(7)
N1B	C1B	C2B	C3B	58.0(9)
N1B	C1B	C2B	C6B	-173.3(6)
N2	N3	C12	C13	-0.2(8)
N2	N3	C12	C15	-178.7(8)
N2	C9_1	C10_1	C11_1	-177.9(7)
N2B	N3B	C12B	C13B	-0.7(8)
N2B	N3B	C12B	C15B	177.9(7)
N2B	C9B	C10B	C11B	-180.0(7)
N3	N2	C14	C13	-0.9(9)
N3	N2	C9_1	C8_1	40.3(10)
N3	N2	C9_1	C10_1	-139.1(7)
N3	C12	C13	C14	-0.3(9)
N3	C12	C13	C18	-179.8(7)
N3	C12	C15	C16	179.1(8)
N3	C12	C15	C42	0.4(14)
N3B	N2B	C9B	C8B	-37.3(10)
N3B	N2B	C9B	C10B	142.5(7)
N3B	N2B	C14B	C13B	1.1(10)
N3B	C12B	C13B	C14B	1.3(9)
N3B	C12B	C13B	C18B	-179.4(8)
N3B	C12B	C15B	C16B	179.5(7)
N3B	C12B	C15B	C42B	-1.1(13)
N9	N16	C57	C60	0.7(9)
N9	N16	C9_2	C8_2	35(3)
N9	N16	C9_2	C10_2	-152(2)
N9	N16	C9_3	C8_3	-146(2)
N9	N16	C9_3	C10_3	30(4)
N9	C21	C55	C20	0.3(12)
N9	C21	C55	C61	179.3(7)
N9	C21	C60	C38	-179.8(7)
N9	C21	C60	C57	0.3(9)
N11	N69	C68	C43	146.3(7)
N11	N69	C68	C70	-33.8(10)
N11	N69	C76	C37	-1.1(9)
N11	C63	C66	C25	1.2(12)
N11	C63	C66	C65	-177.3(7)
N16	N9	C21	C55	-178.8(7)
N16	N9	C21	C60	0.1(8)
N16	C57	C60	C21	-0.6(8)
N16	C57	C60	C38	179.6(9)
N16	C9_2	C10_2	C11_2	-176(3)
N16	C9_3	C10_3	C11_3	-174(3)
N65	C32	C46	C23	60.5(9)
N65	C32	C46	C33	-169.9(6)

Atom	Atom	Atom	Atom	Angle/˚
N65	C39	C64	C23	-57.8(10)
N69	N11	C63	C37	-0.3(8)
N69	N11	C63	C66	175.2(7)
N69	C68	C70	C62	178.9(7)
C1B	N1B	C5B	C4B	62.9(9)
C1B	C2B	C3B	C4B	-53.2(10)
C1B	C2B	C6B	C7B	-107.8(9)
C1B	C2B	C6B	C11B	72.1(9)
C2B	C3B	C4B	C5B	53.6(10)
C2B	C6B	C7B	C8B	-177.6(7)
C2B	C6B	C11B	C10B	177.0(8)
C3B	C2B	C6B	C7B	18.2(12)
C3B	C2B	C6B	C11B	-161.9(8)
C3B	C4B	C5B	N1B	-58.4(10)
C5B	N1B	C1B	C2B	-62.6(9)
C6B	C2B	C3B	C4B	-178.8(7)
C6B	C7B	C8B	C9B	-0.7(12)
C7B	C6B	C11B	C10B	-3.1(12)
C7B	C8B	C9B	N2B	179.3(7)
C7B	C8B	C9B	C10B	-0.5(11)
C8B	C9B	C10B	C11B	-0.1(12)
C9B	N2B	N3B	C12B	-178.4(6)
C9B	N2B	C14B	C13B	179.1(7)
C9B	C10B	C11B	C6B	2.0(12)
C11B	C6B	C7B	C8B	2.5(12)
C12	C13	C14	N2	0.7(8)
C12	C13	C18	C17	1.1(11)
C12	C15	C16	C17	-0.2(12)
C12	C15	C42	O1	2.1(13)
C12	C15	C42	O2	-177.2(9)
C12B	C13B	C14B	N2B	-1.4(9)
C12B	C13B	C18B	C17B	0.4(14)
C12B	C15B	C16B	C17B	0.1(12)
C12B	C15B	C42B	O1B	1.3(12)
C12B	C15B	C42B	O2B	-179.8(7)
C13	C12	C15	C16	0.7(11)
C13	C12	C15	C42	-178.0(8)
C13B	C12B	C15B	C16B	-2.1(11)
C13B	C12B	C15B	C42B	177.3(7)
C14	N2	N3	C12	0.7(8)
C14	N2	C9_1	C8_1	-142.2(8)
C14	N2	C9_1	C10_1	38.4(11)
C14	C13	C18	C17	-178.2(8)
C14B	N2B	N3B	C12B	-0.3(9)
C14B	N2B	C9B	C8B	144.8(8)
C14B	N2B	C9B	C10B	-35.4(11)
C14B	C13B	C18B	C17B	179.4(10)
C15	C12	C13	C14	178.3(7)
C15	C12	C13	C18	-1.2(11)
C15	C16	C17	C18	0.2(13)
C15B	C12B	C13B	C14B	-177.4(7)
C15B	C12B	C13B	C18B	1.9(12)
C15B	C16B	C17B	C18B	2.2(13)
C16	C15	C42	O1	-176.7(8)
C16	C15	C42	O2	4.1(13)
C16	C17	C18	C13	-0.6(11)
C16B	C15B	C42B	O1B	-179.3(8)
C16B	C15B	C42B	O2B	-0.4(12)

Atom	Atom	Atom	Atom	Angle/°
C16B	C17B	C18B	C13B	-2.4(14)
C18	C13	C14	N2	-179.9(8)
C18B	C13B	C14B	N2B	179.4(10)
C20	C55	C61	C56	179.1(7)
C21	N9	N16	C57	-0.5(8)
C21	N9	N16	C9_2	-179.4(16)
C21	N9	N16	C9_3	177.2(18)
C21	C55	C61	C56	0.0(11)
C29	C31	C37	C63	-0.5(11)
C29	C31	C37	C76	177.1(8)
C29	C65	C66	C25	-177.7(7)
C29	C65	C66	C63	0.9(11)
C31	C29	C65	C66	0.6(12)
C31	C37	C63	N11	177.8(7)
C31	C37	C63	C66	1.9(11)
C31	C37	C76	N69	-177.1(8)
C32	N65	C39	C64	64.0(9)
C33	C62	C70	C68	0.9(13)
C37	C63	C66	C25	176.4(7)
C37	C63	C66	C65	-2.1(10)
C38	C56	C61	C55	-0.2(12)
C39	N65	C32	C46	-66.6(9)
C42	C15	C16	C17	178.6(8)
C42B	C15B	C16B	C17B	-179.3(8)
C43	C68	C70	C62	-1.3(12)
C46	C23	C64	C39	53.7(10)
C46	C33	C62	C70	-178.5(8)
C46	C33	C67	C43	178.5(7)
C55	C21	C60	C38	-0.9(11)
C55	C21	C60	C57	179.2(7)
C56	C38	C60	C21	0.7(12)
C56	C38	C60	C57	-179.5(9)
C57	N16	C9_2	C8_2	-143.7(18)
C57	N16	C9_2	C10_2	29(3)
C57	N16	C9_3	C8_3	32(4)
C57	N16	C9_3	C10_3	-153(2)
C60	C21	C55	C20	-178.5(7)
C60	C21	C55	C61	0.5(10)
C60	C38	C56	C61	-0.1(12)
C62	C33	C46	C23	3.4(11)
C62	C33	C46	C32	-121.9(8)
C62	C33	C67	C43	0.3(12)
C63	N11	N69	C68	-178.2(6)
C63	N11	N69	C76	0.8(8)
C63	C37	C76	N69	0.8(8)
C64	C23	C46	C32	-54.8(10)
C64	C23	C46	C33	179.7(7)
C65	C29	C31	C37	-0.8(12)
C67	C33	C46	C23	-174.7(8)
C67	C33	C46	C32	60.0(9)
C67	C33	C62	C70	-0.4(12)
C67	C43	C68	N69	-179.0(7)
C67	C43	C68	C70	1.1(12)
C68	N69	C76	C37	177.9(7)
C68	C43	C67	C33	-0.6(12)
C76	N69	C68	C43	-32.6(12)
C76	N69	C68	C70	147.3(8)
C76	C37	C63	N11	-0.3(9)

Atom	Atom	Atom	Atom	Angle/˚
C76	C37	C63	C66	-176.2(7)
Cl1_1	N1_1	C1_1	C2_1	177.6(4)
Cl1_1	N1_1	C5_1	C4_1	-177.9(4)
N1_1	C1_1	C2_1	C3_1	58.4(7)
N1_1	C1_1	C2_1	C6_1	-171.5(5)
C1_1	N1_1	C5_1	C4_1	64.9(7)
C1_1	C2_1	C3_1	C4_1	-54.3(7)
C1_1	C2_1	C6_1	C7_1	153.2(6)
C1_1	C2_1	C6_1	C11_1	-27.7(9)
C2_1	C3_1	C4_1	C5_1	53.9(7)
C2_1	C6_1	C7_1	C8_1	179.0(7)
C2_1	C6_1	C11_1	C10_1	-177.9(7)
C3_1	C2_1	C6_1	C7_1	-77.8(8)
C3_1	C2_1	C6_1	C11_1	101.2(8)
C3_1	C4_1	C5_1	N1_1	-58.4(7)
C5_1	N1_1	C1_1	C2_1	-64.3(7)
C6_1	C2_1	C3_1	C4_1	175.4(6)
C6_1	C7_1	C8_1	C9_1	0.4(12)
C7_1	C6_1	C11_1	C10_1	1.1(12)
C7_1	C8_1	C9_1	N2	178.9(7)
C7_1	C8_1	C9_1	C10_1	-1.7(12)
C8_1	C9_1	C10_1	C11_1	2.7(12)
C9_1	N2	N3	C12	178.5(6)
C9_1	N2	C14	C13	-178.5(7)
C9_1	C10_1	C11_1	C6_1	-2.4(12)
C11_1	C6_1	C7_1	C8_1	-0.1(12)
Cl1_2	N1_2	C1_2	C2_2	178.8(6)
Cl1_2	N1_2	C5_2	C4_2	-177.5(6)
N1_2	C1_2	C2_2	C3_2	56.2(8)
N1_2	C1_2	C2_2	C6_2	-173.8(7)
C1_2	N1_2	C5_2	C4_2	66.1(8)
C1_2	C2_2	C3_2	C4_2	-52.5(9)
C1_2	C2_2	C6_2	C7_2	178.2(12)
C1_2	C2_2	C6_2	C11_2	-5.7(14)
C2_2	C3_2	C4_2	C5_2	53.9(8)
C2_2	C6_2	C7_2	C8_2	-177.7(16)
C2_2	C6_2	C11_2	C10_2	178.2(17)
C3_2	C2_2	C6_2	C7_2	-52.6(13)
C3_2	C2_2	C6_2	C11_2	123.6(13)
C3_2	C4_2	C5_2	N1_2	-59.8(8)
C5_2	N1_2	C1_2	C2_2	-63.5(8)
C6_2	C2_2	C3_2	C4_2	177.5(7)
C6_2	C7_2	C8_2	C9_2	-5(3)
C7_2	C6_2	C11_2	C10_2	-6(3)
C7_2	C8_2	C9_2	N16	176(3)
C7_2	C8_2	C9_2	C10_2	3(4)
C8_2	C9_2	C10_2	C11_2	-3(4)
C9_2	N16	C57	C60	179.4(19)
C9_2	C10_2	C11_2	C6_2	4(3)
C11_2	C6_2	C7_2	C8_2	6(3)
Cl1_3	N1_3	C1_3	C2_3	177.9(6)
Cl1_3	N1_3	C5_3	C4_3	-177.1(6)
N1_3	C1_3	C2_3	C3_3	57.5(8)
N1_3	C1_3	C2_3	C6_3	-171.1(7)
C1_3	N1_3	C5_3	C4_3	65.8(8)
C1_3	C2_3	C3_3	C4_3	-54.2(8)
C1_3	C2_3	C6_3	C7_3	157.9(12)
C1_3	C2_3	C6_3	C11_3	-21.9(15)

Atom	Atom	Atom	Atom	Angle/°
C2_3	C3_3	C4_3	C5_3	54.7(8)
C2_3	C6_3	C7_3	C8_3	-178.5(19)
C2_3	C6_3	C11_3	C10_3	-179.5(17)
C3_3	C2_3	C6_3	C7_3	-71.7(13)
C3_3	C2_3	C6_3	C11_3	108.5(14)
C3_3	C4_3	C5_3	N1_3	-59.8(8)
C5_3	N1_3	C1_3	C2_3	-63.7(8)
C6_3	C2_3	C3_3	C4_3	174.1(7)
C6_3	C7_3	C8_3	C9_3	-2(4)
C7_3	C6_3	C11_3	C10_3	1(3)
C7_3	C8_3	C9_3	N16	176(3)
C7_3	C8_3	C9_3	C10_3	1(4)
C8_3	C9_3	C10_3	C11_3	1(4)
C9_3	N16	C57	C60	-177.0(18)
C9_3	C10_3	C11_3	C6_3	-2(3)
C11_3	C6_3	C7_3	C8_3	1(3)

Table S6: Hydrogen Fractional Atomic Coordinates (×10⁴) and Equivalent Isotropic Displacement Parameters (Å²×10³) for **cb-e6-013**. U_{eq} is defined as 1/3 of the trace of the orthogonalised U_{ij}.

Atom	x	y	z	U_{eq}
H1	12734.43	-3362.92	-4369.12	64
H1B	9809.44	4548.93	11424.41	64
H4	5691.56	-1053.6	6674.55	64
H10	6751.03	1762.9	559.49	64
H1BA	7493.28	703.9	5057.07	37
H1BB	9075.52	1648.69	5248.37	37
H2B	9553.91	178.67	6122.08	41
H3BA	12341.74	1133.07	7090.67	44
H3BB	11999.06	1968.45	6557.44	44
H4BA	13530.66	683.95	5665.29	49
H4BB	12037	-304.07	5471.83	49
H5BA	11403.1	1179.49	4624.69	42
H5BB	11584	-110.81	3963.67	42
H7B	11102.75	2876.22	7778.14	34
H8B	9890.45	3891.4	9093.68	32
H10B	5620.84	1568.41	7747.77	33
H11B	6826.4	580.76	6423.78	35
H14	18142.67	-2265.95	-1372.74	29
H14B	4332.75	3033.32	8473.44	40
H16	16379.68	-4633.22	-5830.03	33
H16B	6135.92	5792.77	12879.99	35
H17	19245.39	-4229.99	-4729.59	37
H17B	3232.17	5359.28	11748.9	43
H18	19875.78	-3286.1	-2909.45	33
H18B	2616.57	4193.01	9968.76	49
H23A	-1331.51	-3338.64	1779.69	46
H23B	-1291.95	-2012.19	2366.75	46
H29	12698.38	-1281.22	7217.9	38
H31	11545.23	-2039.83	5403.44	35
H32A	1659.53	-3241.92	409.55	44
H32B	294.09	-4069.74	543.69	44
H38	947.02	3155.5	1711.37	35
H39A	-2614.68	-4317.1	-155.78	49
H39B	-3569.29	-3628.65	-775.21	49
H43	6257.49	-1812.49	2911.99	37

Atom	x	y	z	U_{eq}
H46	486.55	-1800.6	1439.88	37
H56	-189.02	2268.02	-88.84	35
H57	4170.18	3657.04	3345.5	31
H61	1588.04	1422.25	-1139.28	30
H62	784.55	-2617.16	3251.33	39
H64A	-2661.19	-2043.33	793.76	54
H64B	-3847.75	-2950.42	924.24	54
H65	10908.46	-564.64	8297.65	34
H67	3643.94	-2067.35	1601.39	36
H70	3372.29	-2373.1	4545.39	39
H76	8331.61	-2460.47	3821.15	34
H1A_1	14733.17	-268.06	2130.8	34
H1B_1	14347.63	803.74	1937.92	34
H2_1	11925.62	-1155.55	984.61	34
H3A_1	10209.03	-532.65	-77.14	42
H3B_1	11642.98	617.76	537.29	42
H4A_1	9325.47	870.5	1282.38	45
H4B_1	9590.45	-205.78	1481.3	45
H5A_1	12191.24	1729.21	2451.68	43
H5B_1	10964.19	1371.67	3053.19	43
H7_1	11350.61	-2296.71	-732.26	36
H8_1	12567.74	-3166.66	-2087.31	36
H10_1	16752.23	-770.31	-598.2	30
H11_1	15558.55	71.46	782.32	34
H1A_2	11054.18	4930.25	6956.48	47
H1B_2	11492.5	3700.82	6726.19	47
H2_2	12647.88	4973.81	5875.7	46
H3A_2	13686.85	3534.15	4831.23	52
H3B_2	13035.05	2804.14	5353.21	52
H4A_2	16055.41	3487.86	6200.22	53
H4B_2	15687.15	4734.93	6461.35	53
H5A_2	14299.69	3425.99	7253.05	52
H5B_2	15984.37	4459.38	7955.43	52
H7_2	11733.98	3817.24	3882.42	37
H8_2	9079.65	3355.75	2515.09	36
H10_2	6300.82	3231.5	4420.02	38
H11_2	8974.38	3711.71	5790.77	34
H1A_3	13037.28	2727.24	5133.02	52
H1B_3	13766.25	4019.11	5531.83	52
H2_3	11274.58	3243.29	6233.81	46
H3A_3	10744.77	4960.15	7043.97	47
H3B_3	12286.51	5389.96	6659.62	47
H4A_3	13578.24	5657.32	8384.93	49
H4B_3	12881.34	4372.19	8026.6	49
H5A_3	15340.33	5114.24	7228.55	52
H5B_3	15898.31	4639.2	8105.32	52
H7_3	8399.69	3539.03	5814.32	34
H8_3	5949.69	3183.27	4382.02	38
H10_3	9198.85	3358.57	2752.14	36
H11_3	11648.9	3611.62	4158.64	37

Table S7: Hydrogen Bond information for **cb-e6-013**.

D	H	A	d(D-H)/Å	d(H-A)/Å	d(D-A)/Å	D-H-A/deg
O1	H1	N3	1.00	1.93	2.804(9)	144.3
O1B	H1B	N3B	1.00	1.92	2.831(9)	149.4
O4	H4	N11	1.00	1.89	2.766(9)	144.4
O10	H10	N9	1.00	1.85	2.767(9)	150.3

Table S8: Atomic Occupancies for all atoms that are not fully occupied in **cb-e6-013**.

Atom	Occupancy	Atom	Occupancy	Atom	Occupancy	Atom	Occupancy
Cl1_2	0.518(4)	C5_2	0.518(4)	Cl1_3	0.482(4)	C5_3	0.482(4)
N1_2	0.518(4)	H5A_2	0.518(4)	N1_3	0.482(4)	H5A_3	0.482(4)
C1_2	0.518(4)	H5B_2	0.518(4)	C1_3	0.482(4)	H5B_3	0.482(4)
H1A_2	0.518(4)	C6_2	0.518(4)	H1A_3	0.482(4)	C6_3	0.482(4)
H1B_2	0.518(4)	C7_2	0.518(4)	H1B_3	0.482(4)	C7_3	0.482(4)
C2_2	0.518(4)	H7_2	0.518(4)	C2_3	0.482(4)	H7_3	0.482(4)
H2_2	0.518(4)	C8_2	0.518(4)	H2_3	0.482(4)	C8_3	0.482(4)
C3_2	0.518(4)	H8_2	0.518(4)	C3_3	0.482(4)	H8_3	0.482(4)
H3A_2	0.518(4)	C9_2	0.518(4)	H3A_3	0.482(4)	C9_3	0.482(4)
H3B_2	0.518(4)	C10_2	0.518(4)	H3B_3	0.482(4)	C10_3	0.482(4)
C4_2	0.518(4)	H10_2	0.518(4)	C4_3	0.482(4)	H10_3	0.482(4)
H4A_2	0.518(4)	C11_2	0.518(4)	H4A_3	0.482(4)	C11_3	0.482(4)
H4B_2	0.518(4)	H11_2	0.518(4)	H4B_3	0.482(4)	H11_3	0.482(4)

Citations

CrysAlisPro (Rigaku, V1.171.44.57a, 2024)

CrysAlisPro (ROD), Rigaku Oxford Diffraction, Poland (?).

O.V. Dolomanov and L.J. Bourhis and R.J. Gildea and J.A.K. Howard and H. Puschmann, Olex2: A complete structure solution, refinement and analysis program, *J. Appl. Cryst.*, (2009), **42**, 339-341.

Sheldrick, G.M., Crystal structure refinement with ShelXL, *Acta Cryst.*, (2015), **C71**, 3-8.
